# Comprehensive Analysis Based on Genes Associated With Cuproptosis, Ferroptosis, and Pyroptosis for the Prediction of Diagnosis and Therapies in Coronary Artery Disease

**DOI:** 10.1155/cdr/9106621

**Published:** 2025-03-15

**Authors:** Yongyi Zhang, Zhehan Guo, Renkui Lai, Xu Zou, Liuling Ma, Tianjin Cai, Jingyi Huang, Wenxiang Huang, Bingcheng Zou, Jinming Zhou, Jinxin Li

**Affiliations:** ^1^Department of Cardiovascular Medicine, The Second Affiliated Hospital of Guangzhou University of Chinese Medicine, Guangdong Provincial Hospital of Chinese Medicine, Guangzhou, Guangdong Province, China; ^2^Department of Cardiovascular Medicine, The First Affiliated Hospital of Jinan University, Guangzhou, Guangdong Province, China; ^3^Schoole of Life Science, Sun Yat-sen University, Guangzhou, Guangdong Province, China

**Keywords:** coronary artery disease, cuproptosis, diagnosis, ferroptosis and pyroptosis, quercetin, STK17B

## Abstract

Coronary artery disease (CAD) is a complex condition influenced by genetic factors, lifestyle, and other risk factors that contribute to increased mortality. This study is aimed at evaluating the diagnostic potential of genes associated with cuproptosis, ferroptosis, and pyroptosis (CFP) using network modularization and machine learning methods. CAD-related datasets GSE42148, GSE20680, and GSE20681 were sourced from the GEO database, and genes related to CFP genes were gathered from MsigDB and FerrDb datasets and literature. To identify diagnostic genes linked to these pathways, weighted gene coexpression network analysis (WGCNA) was used to isolate CAD-related modules. The diagnostic accuracy of key genes in these modules was then assessed using LASSO, SVM, and random forest models. Immunity and drug sensitivity correlation analyses were subsequently performed to investigate possible underlying mechanisms. The function of a potential gene, STK17B, was analyzed through western blot and transwell assays. Two CAD-related modules with strong correlations were identified and validated. The SVM model outperformed LASSO and random forest models, demonstrating superior discriminative power (AUC = 0.997 in the blue module and AUC = 1.000 in the turquoise module), with nine key genes identified: CTDSP2, DHRS7, NLRP1, MARCKS, PELI1, RILPL2, JUNB, STK17B, and SLC40A1. Knockdown of STK17B inhibited cell migration and invasion in human umbilical vein endothelial cells. In summary, our findings suggest that CFP genes hold potential as diagnostic biomarkers and therapeutic targets, with STK17B playing a role in CAD progression.

## 1. Introduction

Coronary artery disease (CAD) is the leading cause of sudden cardiac death in adults, contributing significantly to health-care costs and societal burden [1, 2]. CAD is associated with a high risk of angina, ischemic events, and heart failure, though some patients may remain asymptomatic [3, 4]. Despite advancements in CAD research driven by computer technology, a widely accepted circulating biomarker for CAD detection is still lacking, underscoring the need for a highly sensitive and convenient diagnostic marker.

Cuproptosis, an independent and copper-dependent form of cell death, has recently gained attention for its association with disease processes, including CAD, as evidenced by bioinformatic analyses [5–7]. In myocardial infarction, four cuproptosis-related genes—Dbt, Dlat, Ube2d1, and Ube2d3—were identified as potential diagnostic markers through random forest and the least absolute shrinkage and selection operator (LASSO) analysis [8]. However, research on the role of cuproptosis-related genes in diagnosing CAD remains limited, with only one study exploring cuproptosis biomarkers for CAD [6]. Although cuproptosis is involved in aseptic inflammation and the immune microenvironment [9, 10], its molecular mechanisms in CAD are still unclear, necessitating further exploration for improved diagnostic markers to guide clinical treatment. Ferroptosis, characterized by dysregulated iron metabolism, GPX4 inactivation, and reactive oxygen species production, is crucial in the onset and progression of cell death [11, 12]. Recent studies indicate that ferroptosis plays a significant role in the death of cardiac and vascular cells in various cardiovascular diseases [13, 14]. In atherosclerosis, iron-induced free radicals contribute to low-density-lipoprotein oxidation, a factor in plaque instability and adverse cardiovascular events [15]. Additionally, ferroptosis has been implicated in diabetic cardiomyopathy in animal models [16], and ferroptosis-related genes have been used to identify molecular subtypes of CAD to enable personalized therapeutic approaches [17]. Key genes associated with ferroptosis pathways have been identified in epicardial adipose tissue from CAD patients [18], and the diagnostic potency of 10 ferroptosis-related genes was recently developed to offer new insights into CAD mechanisms [14]. Ferroptosis has also been linked to human umbilical vein endothelial cells (HUVECs) proliferation and migration in CAD [19]. Pyroptosis is a unique form of programmed cell death, distinct from apoptosis due to its caspase-mediated, rapid plasma membrane disruption [20, 21]. Extensive research has shown pyroptosis's involvement in cardiac fibrosis, hypertrophy, and cardiomyocyte death [22]. Inflammation-induced pyroptosis is also reduced in doxorubicin-induced cardiomyopathy [23], and pyroptosis and ferroptosis mediated by MLK3 signaling in cardiomyocytes contribute to myocardial fibrosis in chronic heart failure [24]. J. Gao and Z. Gao demonstrated that USP14 promotes endothelial cell pyroptosis in coronary heart disease [25], while LOXL1-AS1 accumulation enhances pyroptosis in human coronary artery endothelial cells [26]. To date, however, no studies have examined the combined effects of cuproptosis, ferroptosis, and pyroptosis (CFP) in CAD.

In this study, we aim to identify CAD-related modules and genes influencing CAD progression using bioinformatics-based approaches, providing a theoretical foundation for the clinical discovery of novel therapeutic targets.

## 2. Materials and Methods

### 2.1. Data Collection and Cell Culture

RNA expression profiles for CAD and normal samples were obtained from the GEO database (GSE42148, GSE20680, and GSE20681). Through a comprehensive analysis of the source literature, the list of cuproptosis-related genes referred to the previous literature [27, 28]. The ferroptosis-related genes were downloaded through the FerrDb database (http://www.datjar.com) and literature [28]. We searched the MSigDB database [29] (keywords: REACTOME_PYROPTOSIS) to identify pyroptosis-associated markers, suppressive genes, and driver genes. After removing the duplicates, a total of 145 genes associated with CFP genes were collected in later analyses, including 14 cuproptosis-related, 86 ferroptosis-related, and 45 pyroptosis-related genes (Table S1).

HUVECs were acquired from Cell Applications, Inc. (San Diego, United States). All cells were cultured in HyClone DMEM/F12 500 mL medium (Thermo Fisher, United States), supplemented with 10% fetal bovine serum (FBS) (Beyotime Biotechnology, Jiangsu, China) and 1% penicillin/streptomycin (Beyotime Biotechnology, Jiangsu, China), at 37°C in a humidified incubator with 5% CO_2_.

### 2.2. Differential Expression Analysis

Differential analyses were conducted between CAD and normal samples from GSE42148 and GSE20681. Differentially expressed genes (DEGs) were identified based on criteria of |log_2_(fold change)| > 1.5 and *p* < 0.05. Results were visualized using the R package “pheatmap.”

### 2.3. Weighted Gene Coexpression Network Analysis (WGCNA)

WGCNA was applied to identify modules most relevant to CAD. Using the “WGCNA” package in R, the optimal soft threshold was determined to convert the correlation matrix into an adjacency matrix. Genes with similar expression patterns were grouped into gene modules through average linkage hierarchical clustering.

### 2.4. Biomarker Screening by Machine Learning

A support vector machine (SVM) model was cross-validated to calculate regression features using the “e1071” R package, with classification performance evaluated by the area under the receiver operating characteristic (ROC) curve using “pROC” R package. Random forest classification was performed using the “randomForest” package, where variables were ranked by importance to identify key predictors. Additionally, the LASSO regression was used for simultaneous variable selection and parameter estimation. The Connectivity Map (CMap) (https://clue.io) and DGIdb (http://dgidb.genome.wustl.edu/) databases were utilized to identify candidate small molecules for potential therapeutic development.

### 2.5. Immune Infiltration Analysis

Differences in immune infiltration subtypes between CAD and normal samples were assessed. The xCell algorithm was applied using the “xCell” R package to estimate a total of 64 immune and stromal cell types.

Spearman's correlations were calculated to explore the relationships between immune cells and novel genes, with visualizations produced using the “PerformanceAnalytics” and “sankeyD3” packages in R.

### 2.6. Transwell Assay

Stable HUVECs with serine/threonine kinase 17b (STK17B) knockdown were created via transfection with specific shRNA expression plasmids. Briefly, negative control (NC group, 5⁣′-TTCTCCGAACGTGTCACGT-3⁣′) and STK17B-targeting shRNAs (STK17B-shRNA group, 5⁣′-GGACAGGATTGTCGAGCAGA-3⁣′) were synthesized by Invitrogen (Thermo Fisher Scientific Inc.). These shRNA sequences were inserted into BR-V-108 linearized vectors and then transformed into *E. coli.* Additionally, pcDNA3.1-STK17B (STK17B-OE group) and empty vectors (vector group) were sourced from Sangon Biotech, China. Plasmid and shRNA transfections were performed with Lipofectamine 3000 (Invitrogen, United States) according to the manufacturer's protocol. After 48 h, cells were harvested for subsequent analyses, including transwell assays and western blotting.

The transwell assay was conducted as previously described [30, 31]. In brief, 100 *μ*L serum-free medium was added to the upper chamber of a 24-well plate, which was then incubated for 1 h at 37°C. The lower chamber was filled with 600 *μ*L medium containing 20% FBS as a chemoattractant. A 100-*μ*L suspension containing 1 × 10^5^ cells was added to each upper chamber, and the plates were cultured at 37°C with 5% CO_2_ for 8 h. After incubation, 4% paraformaldehyde fixative solution (Beyotime Biotechnology, Jiangsu, China) was added into the lower chamber for 1 h. A cotton swab was used to remove nonmigratory or noninvasive cells, and cells were then stained with 1% crystal violet solution (APPLYGEN, Beijing, China) for 0.5 h. The migrating or invading cells were counted in five randomly selected fields under a microscope and photographed for analysis.

### 2.7. Statistical Analysis

**Figure 1:**
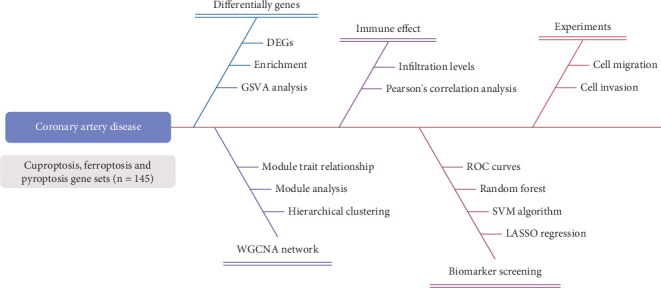
The workflow of cuproptosis-, ferroptosis-, and pyroptosis-related genes identification. DEG, differentially expressed genes; GSVA, gene set variation analysis; WGCNA, weighted gene coexpression network analysis; ROC, receiver operating characteristic; SVM, support vector machine.

Statistical analysis was conducted using Statistical Package for the Social Sciences (SPSS) software (Version 24.0). Differences between the two groups were evaluated using an unpaired, two-tailed Student's *t*-test, while one-way analysis of variance (ANOVA) was applied to assess differences among more than two groups [32]. A *p* value less than 0.05 was considered statistically significant. The study flowchart is presented in Figure 1.

## 3. Results

### 3.1. CFP-Related DEGs

**Figure 2:**
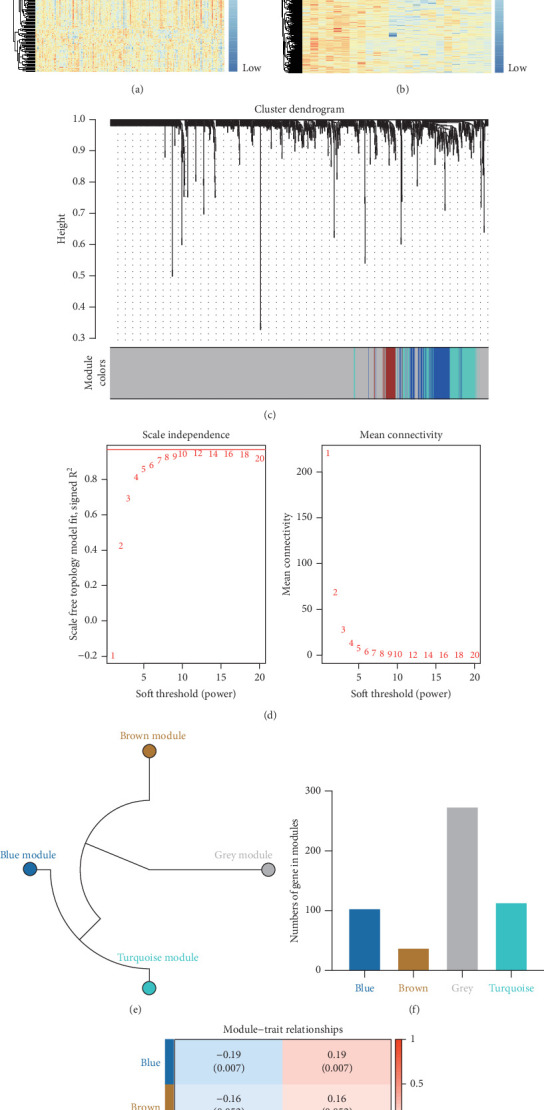
Identification of CAD-associated modules with WGCNA analysis. (a). The DEGs in the GSE20681 database. (b) The DEGs in the GSE42148 database. (c) Cluster dendrogram and module assignment. (d) The identification of soft threshold. (e) The four modules according to the power = 12 as the threshold criteria. (f) The numbers of genes in modules. (g) Module trait relationship and module features of CAD.

To identify potential CAD biomarkers, DEGs were retrieved from the GSE20681 database (99 CAD samples and 99 control samples) and the GSE42148 database (13 CAD samples and 11 control samples). A total of 339 DEGs were identified in GSE20681 using the “limma” R package with thresholds of |log_2_(FC)| > 1.5 and *p* < 0.05. This dataset included 288 upregulated DEGs and 51 downregulated DEGs (Figure 2a, Table S2). From GSE42148, 1808 DEGs were identified, with 995 upregulated and 813 downregulated genes based on the same criteria (Figure 2b, Table S3). After removing duplicates, a set of 2232 unique genes, including all DEGs and 145 CFP genes, was used for WGCNA analysis.

Using the WGCNA algorithm, network clustering analysis grouped these novel genes into four modules, with the optimal soft threshold power set to 12 (Figure 2c,d). The clustering of modules is shown in Figure 2e, and gene counts for each module are presented in Figure 2f. Correlation analysis across all modules identified key modules closely associated with disease occurrence. Results indicated that two modules were linked to CAD progression: the blue module (*p* = 0.007) and the turquoise module (*p* = 0.003) (Figure 2g).

### 3.2. Functional Annotation in Modules

**Figure 3:**
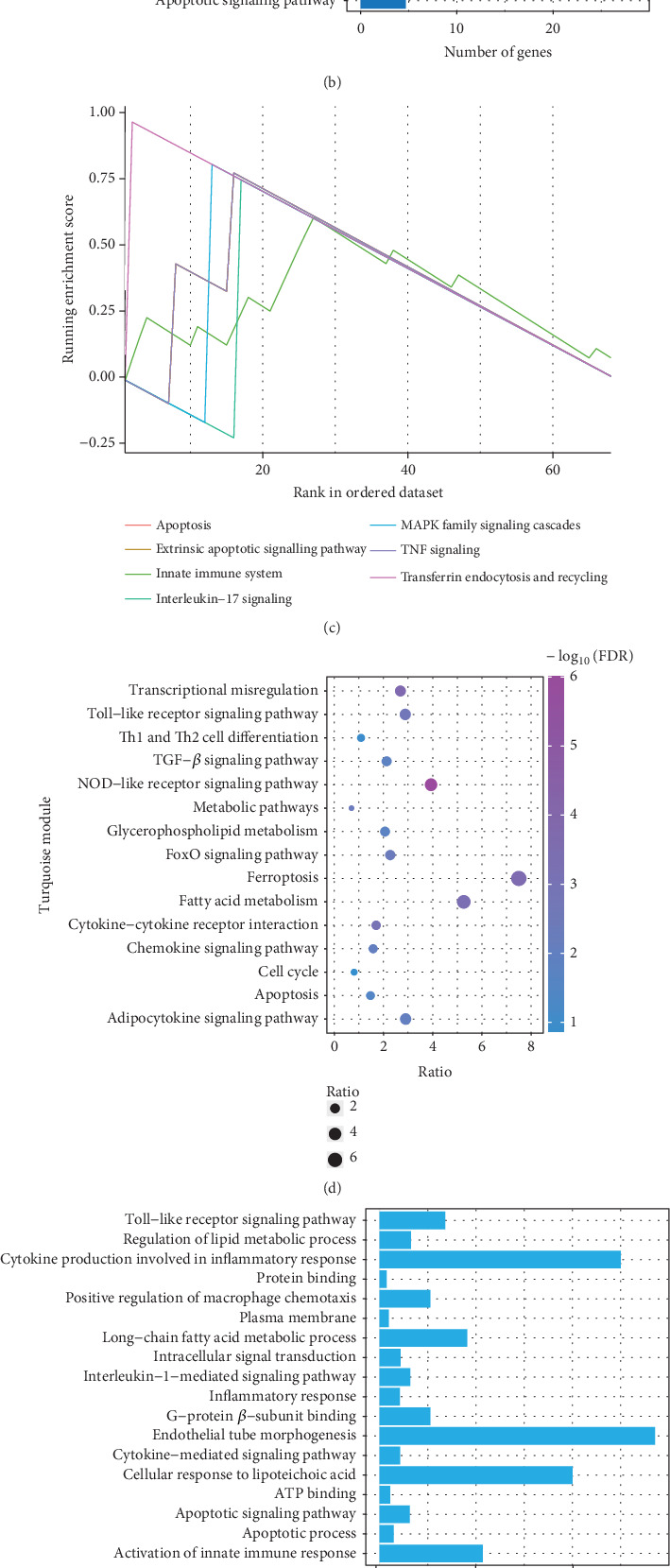
Enrichment analysis in different modules. The (a) KEGG pathways, (b) GO terms, and (c) GSVA signaling in blue modules. The (d) KEGG pathways, (e) GO terms, and (f) GSVA signaling in turquoise modules.

To explore the biological functions and mechanisms of the CAD-associated modules, we performed multifaceted analyses. In the blue module, the Kyoto Encyclopedia of Genes and Genomes (KEGG) analysis showed that dysregulated genes were enriched in pathways related to apoptosis, Th1 and Th2 cell differentiation, transcriptional misregulation, and metabolic pathways (Figure 3a). Gene Ontology (GO) analysis identified terms such as innate immune response, apoptotic signaling, and regulation of smooth muscle cell migration (Figure 3b). Gene set variation analysis (GSVA) further identified pathways like apoptosis, extrinsic apoptotic signalling, and innate immune system (Figure 3c). In the turquoise module, KEGG analysis also indicated pathways associated with apoptosis and immune response, including Th1 and Th2 cell differentiation (Figure 3d). Additionally, turquoise module genes were involved in the cell cycle and fatty acid metabolism. GO terms revealed processes such as apoptotic signaling, activation of innate immune responses, regulation of lipid metabolic metabolism, and positive regulation of macrophage chemotaxis and endothelial tube morphogenesis (Figure 3e). GSVA results confirmed involvement in apoptotic protein cleavage, lipid metabolism, cytokine signaling in the immune system, and cell cycle regulation (Figure 3f).

### 3.3. Characteristic Genes

**Figure 4:**
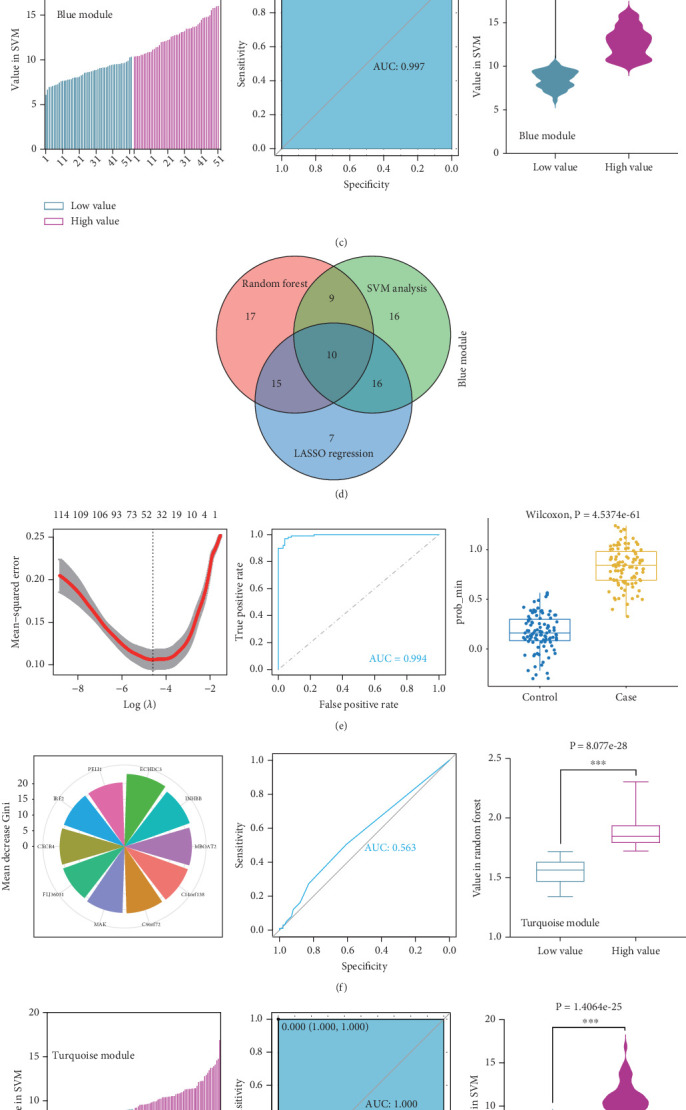
Identification of characteristic genes with machine learning algorithms for CAD. The characteristic genes of blue modules were screened by using (a) LASSO, (b) random forest, and (c) SVM. Venn diagram revealed the intersection of genes through (d) three machine learning algorithms. The characteristic genes of turquoise modules were screened by using (e) LASSO, (f) random forest, and (g) SVM. Venn diagram demonstrated the intersection of genes through (h) three machine learning algorithms.

To identify characteristic genes in CAD-related modules, we used LASSO, random forest, and SVM algorithms on the GEO database. In the blue module, 48 characteristic genes were identified via the LASSO algorithm, achieving an area under the curve (AUC) value of 0.925 (Figure 4a). An additional 51 characteristic genes were selected based on high weight values using the random forest algorithm (Figure 4b), and another 51 were identified using the SVM algorithm (Figure 4c). A total of 10 characteristic genes were defined as overlapping across the three algorithms within the blue module (Figure 4d). In addition, we have performed the validation analysis using GSE20680 datasets, as shown in Figure S1. In the blue module, the AUC value of LASSO in the training set was higher than that in the validation set (0.925 vs. 0.706); the AUC value of random forest in the training set was close to the value in the validation set (0.553 vs. 0.565); the AUC value of SVM in the training set was slightly less than the value in the validation set (0.997 vs. 1.000) (Figure S1A–F). In the turquoise module, 42 characteristic genes were identified by LASSO regression (AUC = 0.994, Figure 4e). Furthermore, 57 characteristic genes were selected with high weight values in both random forest analysis (Figure 4f) and SVM analysis (Figure 4g). In total, eight characteristic genes overlapped across the three algorithms in the turquoise module (Figure 4h). Subsequently, the validation results in the turquoise module showed that the AUC value of LASSO in the training set was also higher than that in the validation set (0.994 vs. 0.879); the AUC value of random forest in the training set was almost close to the value in the validation set (0.563 vs. 0.523); the AUC value of SVM in the training set was consistent with the value in the validation set (1.000 vs. 1.000) (Figure S1G–L).

### 3.4. Infiltrating Immune Cells and Correlation

**Figure 5:**
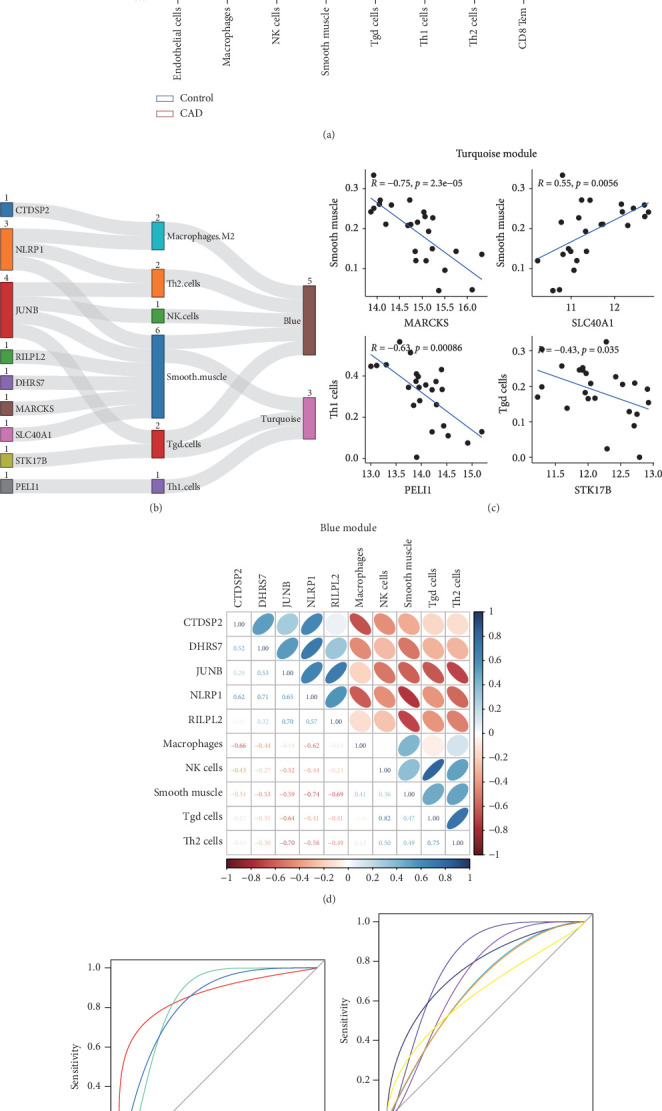
Analysis of immune cell infiltration and its correlation with characteristic genes. (a) Histogram of the differences between CAD and control samples in immune cell infiltration. (b) Analysis of correlation between characteristic genes and immune cells in modules. (c) A total of four genes were significantly related to immune cell in the turquoise module. (d) A total of five genes were significantly associated with immune cell in the blue module. (e) ROC curve showing high classification performance. (f) ROC curve showing low classification performance.

To assess variations in the immune microenvironment in CAD, the enrichment of different immune cells was estimated using the xCell algorithms. The results indicated significantly lower proportions of immune-associated cells in CAD samples, including endothelial cells (*p* = 0.0411), macrophages (*p* = 0.0152), NK (natural killer) cells (*p* = 0.0113), smooth muscle cells (*p* = 0.0326), Tgd (T gamma delta) cells (*p* = 0.0242), Th1 cells (*p* = 0.0233), Th2 cells (*p* = 0.0335), and CD8 Tem (effector memory T cell, *p* = 0.0263) (Figure 5a).

Additionally, Spearman's correlations were performed between characteristic genes and differentially expressed immune cells in the blue and turquoise modules (Figure 5b). As shown in Figure 5c, negative correlations in the turquoise module were observed between MARCKS and smooth muscle (*R* = −0.75, *p* = 2.3*e* − 05); STK17B and Tgd cells (*R* = −0.43, *p* = 0.035); and PELI1 and Th1 cells (*R* = −0.63, *p* = 0.00086). A positive correlation was identified between SLC40A1 and smooth muscle cells (*R* = 0.55, *p* = 0.0056). In the blue module, NLRP1 showed negative correlations with macrophages (*R* = −0.615, *p* = 0.01038); smooth muscle cells (*R* = −0.742, *p* = 0.00065); and Th2 cells (*R* = −0.579, *p* = 0.02068). CTDSP2 was negatively correlated with macrophages (*R* = −0.659, *p* = 0.00447); DHRS7 with smooth muscle cells (*R* = −0.529, *p* = 0.04464); and RILPL2 with smooth muscle cells (*R* = −0.691, *p* = 0.00227). Notably, JunB proto-oncogene (JUNB) showed negative correlations with NK cells (*R* = −0.523, *p* = 0.04689); smooth muscle cells (*R* = −0.593, *p* = 0.01632); Tgd cells (*R* = −0.639, *p* = 0.00660); and Th2 cells (*R* = −0.699, *p* = 0.00216) (Figure 5d).

ROC curves illustrated the diagnostic value of all the nine pivotal genes, as shown in Figure 5e,f. The results indicated high classification performance for CTDSP2 (AUC = 0.8626), STK17B (AUC = 0.8112), and PELI1 (AUC = 0.8169). Additionally, small molecules were identified as potential therapeutic candidates from the CMap and DGIdb databases, including quercetin and dexamethasone (Table S4). Based on these findings, we speculate that STK17B may demonstrate heightened sensitivity to drug response and possess strong diagnostic potential in CAD. Therefore, we focused further on examining the effects of STK17B on cell migration and invasion.

### 3.5. The Effects of STK17B on Cell Migration and Invasion

**Figure 6:**
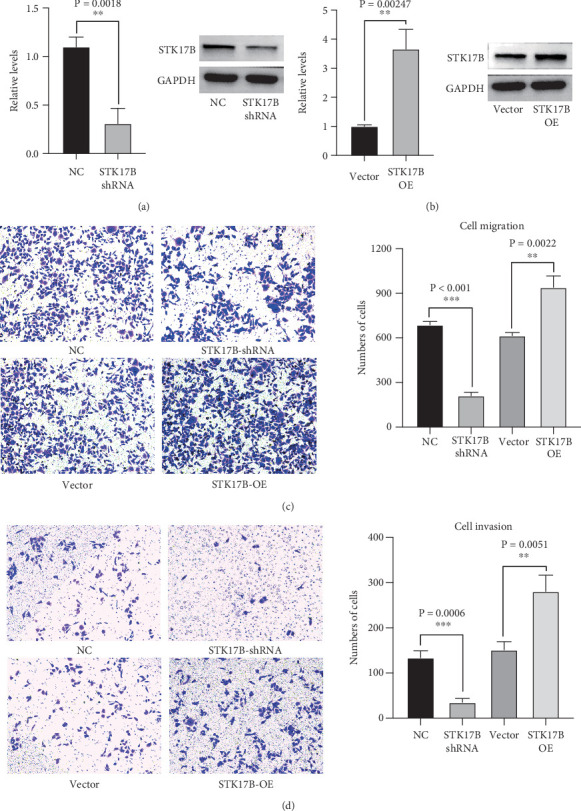
The effects of STK17B on cell migration and invasion. (a) Knockdown efficiency. (b) Overexpression (OE) efficiency. (c) Migration assay analysis. (d) Invasion assay analysis.

To investigate the impact of STK17B on the cell migration and invasion, a transwell assay was conducted in HUVECs. Western blot analysis confirmed that STK17B was successfully knocked down (Figure 6a) and overexpressed in HUVECs (Figure 6b). Results showed that downregulation of STK17B significantly inhibited cell migration and invasion, whereas STK17B overexpression (OE) markedly promoted cell migration (Figure 6c) and invasion abilities (Figure 6d).

## 4. Discussion

CAD is a multifactorial disease influenced by genetic factors, lifestyle, and other risk elements [33]. Previous studies have shown that CAD, the most common type of cardiovascular disease, can lead to heart attacks, posing a significant threat to public health [34, 35]. The high morbidity and mortality rates associated with CAD are largely due to the challenges in achieving early diagnosis [36]. Currently, diagnostic methods for CAD include noninvasive techniques (such as electrocardiograms and treadmill exercise tests) [37, 38] and invasive techniques (like coronary angiography and intravascular ultrasound) [39, 40]. However, each method has limitations. Electrocardiograms often show poor sensitivity and specificity [41]. Although studies have indicated that treadmill exercise tests may have higher diagnostic value for coronary heart disease in elderly patients, the extent and duration of ST depression show no significant difference, and older patients often have lower endurance for such tests [42]. Furthermore, many low-income families cannot afford the costs of invasive examinations [43]. Consequently, there is a pressing need to identify biomarkers for early detection and risk assessment in CAD patients.

Recent advancements in genetic sequencing and technology have opened new avenues for understanding the molecular mechanisms behind CAD development and progression, identifying novel targets for potential pharmacological intervention [17, 44]. Studies have linked processes like CFP to various diseases, including CAD [6, 14, 45]. Liu et al. identified GLS as a cuproptosis-related diagnostic gene in acute myocardial infarction [46]. To date, only one study has explored the potential biomarkers associated with cuproptosis in CAD [6]. A recent study developed a diagnostic signature and examined immune infiltration in ischemic cardiomyopathy based on cuproptosis-related genes [47]. The cuproptosis-related gene Ube2d3 emerged as a central biomarker in myocardial infarction, with findings showing that Ube2d3 promotes hypoxia-induced damage in AC16 cells by inducing cuproptosis [8]. To identify potential biomarkers with high diagnostic value, we examined 14 cuproptosis-associated genes from MsigDB, including GSL and MTF1 (Table S1). Notably, a recent study found that MTF1 promotes myogenesis in response to copper [48].

Additionally, genetic analyses have reported potential biomarkers and therapeutic targets related to ferroptosis in CAD and other heart diseases [17, 44]. In myocardial infarction, studies investigated the molecular roles of ferroptosis-related genes in disease progression [49]. Deficiency or inhibition of Nrf2 exacerbated myocardial infarction–induced cardiomyocyte ferroptosis by reducing xCT and GPX4 expression, both in vivo and in vitro, suggesting that myocardial infarction is accompanied by cardiomyocyte ferroptosis [50]. Inhibition of ferroptosis was shown to suppress calcification in coronary artery vascular smooth muscle cells via the P53/SLC7A11 pathway [51]. Furthermore, polyphenol drugs that inhibit ferroptosis were found to alleviate myocardial injury through the KAT5/GPX4 pathway in myocardial infarction [52]. Studies have also investigated the occurrence and effects of ferroptosis during ischemia and reperfusion phases in acute myocardial infarction, occurring within 6 h in rats [53]. Recent findings demonstrated that inhibiting H/R-induced apoptosis and ferroptosis via shock wave therapy could protect cardiomyocyte function in CAD [54]. These findings indicate that inhibiting ferroptosis plays a crucial role in the progression of CAD. Furthermore, studies on ferroptosis-related genes identified CBS, HSPB1, and CEBPG as potential diagnostic markers in CAD [14]. Expressions of LONP1 and HSPB1 were linked to delayed CAD progression [17]. NLRP1 levels were associated with the severity of coronary artery stenosis in CAD patients [55]. Research has shown that NLRP1, a well-known inflammasome, contributes to the progression of atherosclerosis, myocardial infarction, and heart failure [56]. Our results similarly demonstrated that NLRP1 was identified in the blue module with a moderate diagnostic value (AUC = 0.7964) through the random forest, SVM, and LASSO analyses. A recent study also revealed that serum NLRP1 levels independently predict coronary artery calcification [57]. Moreover, enrichment analysis results in the blue module indicated the involvement of inflammatory response signaling and the IL-17 signaling pathway.

Additionally, an increasing number of studies indicate that pyroptosis-related genes contribute to the progression of coronary artery calcification [58]. Recent research has shown that the PELI1 inflammasome promotes pyroptosis in cardiovascular disease [59]. Specifically, PELI1 deletion in macrophages was found to reduce myocardial ischemia/reperfusion injury [60]. Another study demonstrated that PELI1 exacerbates myocardial ischemia/reperfusion injury by impairing autophagy flux [61]. Moreover, pyroptosis has been identified as a potential biomarker across various heart diseases [24, 62, 63]. Drugs that inhibit caspase-3/GSDME-mediated pyroptosis have been shown to alleviate the progression of coronary artery calcification [63]. Additionally, monocytes were directed towards pyroptosis through AIM2 inflammasome activation in CAD, suggesting that pyroptosis plays a role in CAD development and progression [64]. A recent report revealed that the FOXC1-JAK2 regulatory pathway might have a reverse regulatory function in CAD-related pyroptosis [65].

However, identifying specific biomarkers to predict CAD risk or disease progression remains an area of active research. Such biomarkers could potentially aid in the development of therapeutic and diagnostic strategies [66]. Feng et al. constructed a diagnostic model for advanced-stage CAD, aimed at predicting both diagnostic and therapeutic values [67]. In our study, we explored CAD-related modules using the WGCNA algorithm, an advanced system biology–based approach that links genetic information to phenotypic traits and aids in investigating molecular mechanisms [68]. Similar to our findings, WGCNA analysis has previously highlighted green modules as being closely associated with CAD [69]. Peng, Sun, and Zhang identified nine coexpression modules related to CAD using WGCNA [70]. WGCNA has also been employed to screen disease-associated modules in ischemic cardiomyopathy–related heart failure [71] and coexpression modules for immune gene signatures in diabetic cardiomyopathy [72]. Our results revealed that two modules had a moderate positive correlation with clinical traits, suggesting that these modules may be linked to CAD pathologies. Unlike our findings, another study used WGCNA to investigate M2 macrophage-associated biomarkers [73] or pathways in CAD [74]. This is consistent with previous findings, where the three most significant modules were identified as lipid metabolism biomarkers in CAD patients [75].

To further explore novel genes within these modules, three machine learning analyses were performed to develop classification models. Our results identified a total of 10 novel genes in the blue module and eight in the turquoise module that intersected with machine learning algorithms. Similar to our findings, they have proven to be reliable tools for accurately predicting in-hospital mortality risk in patients with chronic kidney disease [76]. Three machine learning classifiers (LASSO, SVM, and XGBoost) were used to construct and select the best predictive model for hemodynamically significant CAD [77]. Additionally, Pearson's correlation analysis in our study revealed that nine genes, including CTDSP2, NLRP1, JUNB, RILPL2, DHRS7, MARCKS, SLC40A1, STK17B, and PELI1, were strongly correlated with immune cells. Moreover, ROC results demonstrated higher classification efficacy with elevated AUC values for genes like PELI1, CTDSP2, and STK17B. Previous studies have suggested PELI1 as a biomarker to improve the prediction, prevention, and treatment of stroke in atrial fibrillation patients [78], consistent with our results. Furthermore, our findings identified potential therapeutic molecules, such as quercetin and dexamethasone, through the CMap and DGIdb databases. Mandaviya et al. highlighted CTDSP2 as a key factor in leukocytes using a genetic risk score model analysis [79]. STK17B is involved in protein serine/threonine kinase activity and plays a role in regulating fibroblast apoptotic processes [80]. While no direct evidence yet connects STK17B to heart disease, numerous studies associate dysregulated STK17B expression with poor clinicopathological features in hepatocellular carcinoma [81, 82]. STK17B was also identified as a significant hub gene involved in the pathogenesis of sepsis [83], aligning with our results. STK17B showed high diagnostic performance (AUC = 0.8112) as a potential hub biomarker in our study, marking the first exploration of STK17B's biological function in cell migration and invasion in CAD. Similarly, Jiang et al. demonstrated that silencing STK17B suppressed cell progression in ovarian cancer cells [84]. A recent study further revealed that T cell functions, including migration, are reduced in the absence of STK17B [85]. Consistent with previous findings, our research shows that STK17B silencing significantly inhibited cell migration and invasion. JUNB (AP-1 transcription factor subunit) encodes a protein involved in sequence-specific double-stranded DNA binding activity [86]. JUNB participates in calcium signaling within the CD4+ TCR pathway and cytokine signaling in the immune system [87]. Similarly, our results indicated a negative correlation between JUNB and Tgd cells (*R* = −0.63, *p* = 0.00092), suggesting an association between JUNB and immune pathways. Additionally, JUNB alerts Tregs of the emerging Teff activation [88] and activates a transcriptional program that sustains CD25-Tregs, leading to the absence of T follicular regulatory cells when JunB is deficient [89]. Other studies have shown that JUNB regulates homeostasis and suppressive functions of effector regulatory T cells [90]. These findings highlight potential diagnostic genes related to CAD, and we will next investigate the impact of the signaling pathways involving these genes on CAD progression.

It is well known that the signaling pathways play a critical role in the progression of CAD. Evidence has shown that apoptotic pathways contribute to modulating cardiomyocyte survival and to ischemic myocardial injury [91]. An imbalance of Th1 and Th2 cytokines is also present in cardiac injuries [92]. Consistent with our findings, the genes in the blue module were primarily associated with apoptosis, Th1 and Th2 cell differentiation, and innate immune responses. Additionally, cell cycle-related genes are known to be regulated and implicated in the pathogenesis of CAD [93]. Loss-of-function mutations in the apolipoprotein C3 gene, involved in triglyceride metabolism, reduce CAD risk [94]. Similarly, our results reinforced that these pathways are strongly connected to CAD. Our findings further indicated that genes in the turquoise module were not only enriched in cell cycle and metabolism pathways but also associated with immune and inflammatory responses. Supporting our results, studies have reported that innate and adaptive immune responses are activated to clear dead and apoptotic cells in CAD [95]. Molecular pathways related to oxidative stress and ferroptosis have also been associated with CAD risk factors [96]. A recent study highlighted the protective role of B cells in remodeling atheromatous plaques [97]. However, we did not observe significant differences in B cell levels between control and CAD samples in our data. Meanwhile, our analysis showed a lower accumulation of Th1 cells (Figure 5, *p* = 0.0233) and Th2 cell (Figure 5, *p* = 0.0335) in CAD samples. Th1 cells are proatherogenic, primarily through IFN-*γ* production [95, 98]. Conversely, He et al. reported no significant differences in Th1 cells between control and CAD samples [99], while Ding et al. observed increased frequencies of Th1-like phenotypes in CAD patients [100]. Another study indicated that the expression of certain genes in Th1 pathways, such as IL1 and IL18, was significantly elevated, whereas GATA3 expression was significantly reduced in CAD patients compared to controls [101]. Our results also revealed a negative correlation between Th2 cells and CFP-related genes. Additionally, research has shown a negative correlation between Th2 cells and serum BDNF in coronary heart disease patients [102]. Moreover, IL-17 levels in the CAD group were significantly lower and served as independent predictors of CAD [103], suggesting that IL-17 plays a crucial role in the progression of CAD. Our findings identified the IL-17 signaling pathway as relevant to the inflammatory response in the blue module in CAD patients. Consistent with the previous studies, blocking IL-17 in mice has been shown to promote atherosclerosis development [104], and IL-17 signaling pathway was also identified as the potent underlying pathogenesis of CAD [105].

Furthermore, CMap analysis revealed that these genes were involved in multiple drugs, including quercetin and dexamethasone. CMap is a tool that utilizes gene-expression signatures to link small molecules, genes, and diseases [106]. Dexamethasone has shown potential in improving outcomes after myocardial infarction [107]. It can minimize fluid retention and prevent mineralocorticoid-induced cell proliferation within plaques. One case report described the successful use of dexamethasone in a patient with a total artificial heart [108]. Similarly, our findings identified dexamethasone as relevant to CAD, aligning with recent reports in which several approved drugs, including dexamethasone, were highlighted through in silico models in CAD research [109]. Additionally, low-dose dexamethasone combined with luteolin has been shown to improve myocardial infarction recovery [110]. Many studies have examined the beneficial effects of quercetin, especially its positive impact on cardiovascular health [111, 112]. Quercetin has been associated with lowering blood pressure and reducing inflammation in cardiovascular disease [113]. In patients with CAD, quercetin has been shown to lower IL-1*β* and IL-10 levels, demonstrating its anti-inflammatory properties [114]. This aligns with our findings, where quercetin was identified as a potential therapeutic agent for CAD. However, other drugs may offer superior therapeutic effects compared to quercetin in treating CAD. For example, resveratrol has shown greater efficacy than quercetin in reducing endothelium degeneration and lowering necrosis factor *α* levels in CAD patients [115]. Unlike quercetin, resveratrol did not appear in our results, possibly because we focused on CFP-related genes rather than necrosis-associated genes and pathways. We also found that quercetin intersected with STK17B with high scores. The expression of STK17B is responsive to inflammatory lymphokine stimulation [116]. In future work, we plan to investigate the relationship between STK17B and inflammation in response to quercetin in CAD. Additionally, further in vitro experiments will be conducted to evaluate the efficacy of multiple drugs.

This study has certain limitations that warrant consideration. Our findings were derived from public datasets and cell-based experiments; therefore, they require further validation in larger and more diverse cohorts. Additionally, the expression levels and biological functions of other gene signatures still need to be explored.

## 5. Conclusion

In conclusion, WGCNA and machine learning algorithms identified STK17B as a key gene enriched in CFP pathways in CAD. The role of STK17B warrants further attention to enhance our understanding of CAD progression.

## Data Availability

This study utilized previously reported public data available in the GEO database. Experimental data are available upon request from the corresponding author.

## Supporting Information

Supporting Information 1 Figure S1: Validation of the performance of LASSO, SVM, and random forest in GSE20680 datasets. ROC curve (a) and the feature importance (b) of LASSO in blue modules. The ROC curve (c) and the feature value (d) of random forest analysis in blue modules. The ROC curve (e) and the feature value (f) of SVM algorithm in blue modules. ROC curve (g) and the feature importance (h) of LASSO in turquoise modules. The ROC curve (i) and the feature value (j) of random forest analysis in turquoise modules. The ROC curve (k) and the feature value (l) of SVM algorithm in turquoise modules.

Supporting Information 2 Table S1: The list of cuproptosis-, ferroptosis-, and pyroptosis-related genes.

Supporting Information 3 Table S2: The differentially expressed genes between CAD and control samples in GSE20681.

Supporting Information 4 Table S3: The differentially expressed genes between CAD and control samples in GSE42148.

Supporting Information 5 Table S4: The small molecules drugs targeting in genes from Connective Map (CMap) and DGIdb database.
