# Supplementary figures and images for: Comprehensive Analysis Based on Genes Associated With Cuproptosis, Ferroptosis, and Pyroptosis for the Prediction of Diagnosis and Therapies in Coronary Artery Disease
Source: Cardiovasc Ther. 2025 Mar 15;2025:9106621. doi: 10.1155/cdr/9106621 (PMC11929595; doi:10.1155/cdr/9106621)

**A**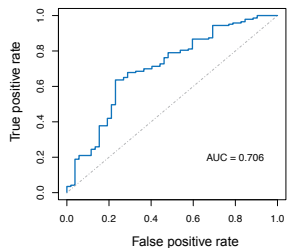**B**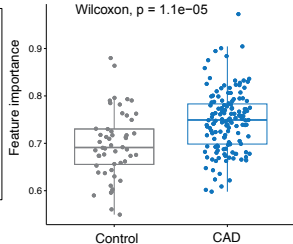**C**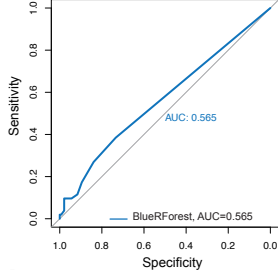**D**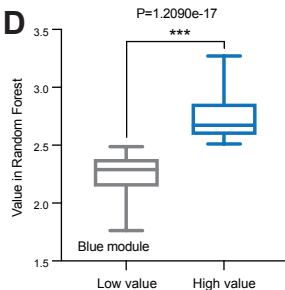**E**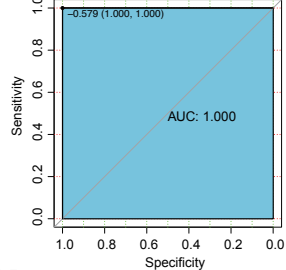**F**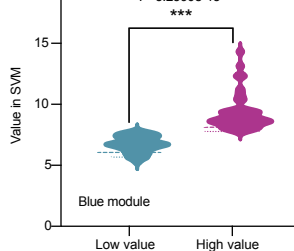**G**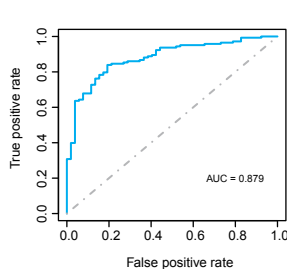**H**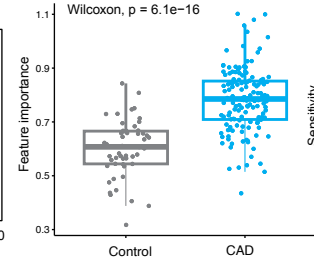**I**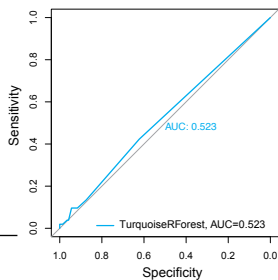**J**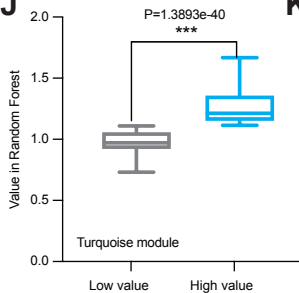**K**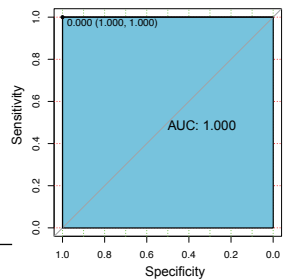**L**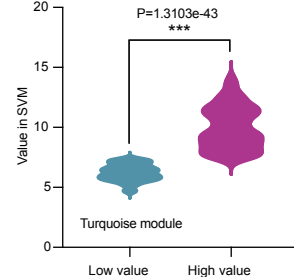

Supplement: Supporting Information 1 — Figure S1: Validation of the performance of LASSO, SVM, and random forest in GSE20680 datasets. ROC curve (a) and the feature importance (b) of LASSO in blue modules. The ROC curve (c) and the feature value (d) of random forest analysis in blue modules. The ROC curve (e) and the feature value (f) of SVM algorithm in blue modules. ROC curve (g) and the feature importance (h) of LASSO in turquoise modules. The ROC curve (i) and the feature value (j) of random forest analysis in turquoise modules. The ROC curve (k) and the feature value (l) of SVM algorithm in turquoise modules. [file 9106621.f1.pdf]
