# Supplementary material for: Comprehensive Analysis Based on Genes Associated With Cuproptosis, Ferroptosis, and Pyroptosis for the Prediction of Diagnosis and Therapies in Coronary Artery Disease
Source: Cardiovasc Ther. 2025 Mar 15;2025:9106621. doi: 10.1155/cdr/9106621 (PMC11929595; doi:10.1155/cdr/9106621)
Supplement: Supporting Information 2 — Table S1: The list of cuproptosis-, ferroptosis-, and pyroptosis-related genes. [file 9106621.f2.pdf]

Supplementary Table 1. The list of cuproptosis, ferroptosis and pyroptosis related genes

| Symbol | Type |
|---|---|
| FDX1 | cuproptosis |
| LIAS | cuproptosis |
| LIPT1 | cuproptosis |
| DLD | cuproptosis |
| DLAT | cuproptosis |
| PDHAI | cuproptosis |
| PDHB | cuproptosis |
| MTF1 | cuproptosis |
| GLS | cuproptosis |
| CDKN2A | cuproptosis |
| GCSH | cuproptosis |
| ATP7A | cuproptosis |
| ATP7B | cuproptosis |
| SLC31A1 | cuproptosis |
| ABCC1 | ferroptosis |
| ACACA | ferroptosis |
| ACO1 | ferroptosis |
| ACSF2 | ferroptosis |
| ACSL1 | ferroptosis |
| ACSL3 | ferroptosis |
| ACSL4 | ferroptosis |
| ACSL5 | ferroptosis |
| ACSL6 | ferroptosis |
| AIFM2 | ferroptosis |
| AKR1C1 | ferroptosis |
| AKR1C2 | ferroptosis |
| AKR1C3 | ferroptosis |
| ALOX12 | ferroptosis |
| ALOX15 | ferroptosis |
| ALOX5 | ferroptosis |
| ATG5 | ferroptosis |
| ATG7 | ferroptosis |
| ATP5MC3 | ferroptosis |
| BACH1 | ferroptosis |
| CARS | ferroptosis |
| CBS | ferroptosis |
| CD44 | ferroptosis |
| CHAC1 | ferroptosis |

| | |
|---|---|
| CISD1 | ferroptosis |
| CP | ferroptosis |
| CRYAB | ferroptosis |
| DPP4 | ferroptosis |
| EMC2 | ferroptosis |
| FADS2 | ferroptosis |
| FANCD2 | ferroptosis |
| FDFT1 | ferroptosis |
| FTH1 | ferroptosis |
| FTL | ferroptosis |
| FTMT | ferroptosis |
| G6PD | ferroptosis |
| GCLC | ferroptosis |
| GCLM | ferroptosis |
| GLS2 | ferroptosis |
| GOT1 | ferroptosis |
| GPX4 | ferroptosis |
| GSS | ferroptosis |
| HMGCR | ferroptosis |
| HMOX1 | ferroptosis |
| HSBP1 | ferroptosis |
| HSPB1 | ferroptosis |
| IREB2 | ferroptosis |
| KEAP1 | ferroptosis |
| LPCAT3 | ferroptosis |
| MAP1LC3A | ferroptosis |
| MAP1LC3B | ferroptosis |
| MAP1LC3C | ferroptosis |
| MT1G | ferroptosis |
| NCOA4 | ferroptosis |
| NFE2L2 | ferroptosis |
| NFS1 | ferroptosis |
| NOX1 | ferroptosis |
| NQO1 | ferroptosis |
| NRF2 | ferroptosis |
| OTUB1 | ferroptosis |
| PCBP1 | ferroptosis |
| PCBP2 | ferroptosis |
| PEBP1 | ferroptosis |
| PGD | ferroptosis |
| PHKG2 | ferroptosis |
| PRNP | ferroptosis |
| PROM2 | ferroptosis |

| | |
|---|---|
| PTGS2 | ferroptosis |
| RPL8 | ferroptosis |
| SAT1 | ferroptosis |
| SAT2 | ferroptosis |
| SLC11A2 | ferroptosis |
| SLC1A5 | ferroptosis |
| SLC39A14 | ferroptosis |
| SLC39A8 | ferroptosis |
| SLC3A2 | ferroptosis |
| SLC40A1 | ferroptosis |
| SLC7A11 | ferroptosis |
| SQLE | ferroptosis |
| STEAP3 | ferroptosis |
| TF | ferroptosis |
| TFRC | ferroptosis |
| TP53 | ferroptosis |
| VDAC2 | ferroptosis |
| VDAC3 | ferroptosis |
| ZEB1 | ferroptosis |
| AIM2 | pyroptosis |
| CASP1 | pyroptosis |
| CASP3 | pyroptosis |
| CASP4 | pyroptosis |
| CASP5 | pyroptosis |
| CASP6 | pyroptosis |
| CASP8 | pyroptosis |
| CASP9 | pyroptosis |
| ELANE | pyroptosis |
| GPX4 | pyroptosis |
| GSDMA | pyroptosis |
| GSDMB | pyroptosis |
| GSDMC | pyroptosis |
| GSDMD | pyroptosis |
| GSDME | pyroptosis |
| IL18 | pyroptosis |
| IL1B | pyroptosis |
| IL6 | pyroptosis |
| NLRC4 | pyroptosis |
| NLRP1 | pyroptosis |
| NLRP2 | pyroptosis |
| NLRP3 | pyroptosis |
| NLRP6 | pyroptosis |
| NLRP7 | pyroptosis |

| | |
|---|---|
| NOD1 | pyroptosis |
| NOD2 | pyroptosis |
| PJVK | pyroptosis |
| PLCG1 | pyroptosis |
| PRKACA | pyroptosis |
| PYCARD | pyroptosis |
| SCAF11 | pyroptosis |
| TIRAP | pyroptosis |
| TNF | pyroptosis |
| CYCS | pyroptosis |
| GZMB | pyroptosis |
| HMGB1 | pyroptosis |
| IRF1 | pyroptosis |
| IRF2 | pyroptosis |
| TLR3 | pyroptosis |
| TLR4 | pyroptosis |
| RIPK1 | pyroptosis |
| DIABLO | pyroptosis |
| CARD8 | pyroptosis |
| WNK1 | pyroptosis |
| BRD4 | pyroptosis |
