# Supplementary material for: Comprehensive Analysis Based on Genes Associated With Cuproptosis, Ferroptosis, and Pyroptosis for the Prediction of Diagnosis and Therapies in Coronary Artery Disease
Source: Cardiovasc Ther. 2025 Mar 15;2025:9106621. doi: 10.1155/cdr/9106621 (PMC11929595; doi:10.1155/cdr/9106621)
Supplement: Supporting Information 3 — Table S2: The differentially expressed genes between CAD and control samples in GSE20681. [file 9106621.f3.pdf]

Supplementary Table 2. The differentially expressed genes between CAD and control samples in GSE20681

DEG	logFC	AveExpr	t	P.Value	adj.P.Val	B
IL18RAP	6.6651121	189.27539	3.7942583	0.000197	0.999912	-3.413347
MMP9	5.7908361	222.041	2.9419236	0.0036527	0.999912	-3.90697
MYBPH	5.4126932	141.81605	2.8981474	0.0041788	0.999912	-3.929533
CLEC4D	5.3131874	152.05425	3.316122	0.0010866	0.999912	-3.702648
TPST1	5.2920808	137.74152	2.9544021	0.0035142	0.999912	-3.900486
CCL3	5.1555008	136.65243	2.1868849	0.0299281	0.999912	-4.254282
CLEC4E	4.5791431	196.18661	2.8550066	0.0047642	0.999912	-3.951486
RAB36	4.1909275	122.14123	3.0334154	0.0027436	0.999912	-3.858893
TNFAIP6	4.1866459	137.10007	2.7855707	0.0058649	0.999912	-3.986227
LOC283767	4.0623742	132.83602	2.3844153	0.018055	0.999912	-4.172221
ASPRV1	4.0066159	180.39904	3.1546839	0.0018584	0.999912	-3.793275
TREM1	3.8324447	174.5728	2.905119	0.0040906	0.999912	-3.925959
LOC151438	3.7720177	144.97106	2.4390874	0.0156097	0.999912	-4.14838
IL1R2	3.6069969	166.95616	2.6026798	0.0099529	0.999912	-4.074175
DUSP1	3.5879777	182.67485	2.8330081	0.0050906	0.999912	-3.962572
IGFBP2	3.5186536	130.21235	1.972983	0.0498954	0.999912	-4.335801
NAMPT	3.4399857	215.65843	2.8502116	0.0048337	0.999912	-3.953909
CEACAM4	3.4202848	174.81676	2.9540539	0.003518	0.999912	-3.900668
ANXA3	3.3793347	152.89926	2.3134424	0.0217298	0.999912	-4.202444
QPCT	3.3345236	174.03345	2.5707788	0.0108844	0.999912	-4.088979
NOD2	3.3038486	148.282	2.7384154	0.0067392	0.999912	-4.009399
ACSL1	3.293273	194.19192	2.3345699	0.0205727	0.999912	-4.193533
G0S2	3.2364805	130.01241	2.2296417	0.0268999	0.999912	-4.237066
TNFRSF10[illegible]	3.2359588	215.42842	3.1713696	0.0017598	0.999912	-3.78408
BMX	3.2025279	115.3763	2.6260074	0.0093177	0.999912	-4.063248
PAQR6	3.200631	145.94705	2.6600583	0.0084559	0.999912	-4.047144
C20orf3	3.1872805	175.31645	3.0343743	0.0027353	0.999912	-3.858383
MGAM	3.1176839	144.58475	2.2392186	0.0262593	0.999912	-4.233168
THBD	3.1169692	160.43268	2.2644237	0.0246366	0.999912	-4.222837
GPR177	3.0661205	113.67596	2.1992794	0.0290213	0.999912	-4.249323
HSPA1A	3.0510364	174.23596	2.1511653	0.0326802	0.999912	-4.268431
CEBPD	3.0506859	216.15118	2.1382296	0.0337293	0.999912	-4.273503
RALB	3.0308592	132.12158	2.1187227	0.0353663	0.999912	-4.281097
MMP25	3.022934	209.07925	2.4268594	0.0161296	0.999912	-4.153754
FKBP5	3.0228913	133.07293	2.3031024	0.0223166	0.999912	-4.206779
MANSC1	3.0164122	122.50404	3.0572671	0.0025436	0.999912	-3.846156
GPR160	3.0086397	137.8079	2.3876892	0.0178995	0.999912	-4.170807
FOS	2.9798928	153.51254	2.3556961	0.0194702	0.999912	-4.18455
MBD6	2.9630182	155.8754	3.1886226	0.0016629	0.999912	-3.77453
ECHDC3	2.943351	116.68826	2.3084599	0.0220109	0.999912	-4.204535

BCL3	2.93399	195.40674	3.1030975	0.0021965	0.999912	-3.82145
CA4	2.9129219	127.94607	2.151292	0.03267	0.999912	-4.268381
TLR4	2.8993137	137.53948	2.7506714	0.0065013	0.999912	-4.003409
ABHD5	2.8706299	166.68838	2.7678028	0.0061815	0.999912	-3.994998
FAM129A	2.827732	181.87803	2.605323	0.009879	0.999912	-4.072941
CKAP4	2.8035961	141.43594	1.992345	0.0477133	0.999912	-4.328741
RNF149	2.788501	235.80062	2.497415	0.013329	0.999912	-4.122413
RNF144B	2.7836093	156.2636	2.3295547	0.0208424	0.999912	-4.195655
DDIT4	2.7602093	156.71803	2.2108628	0.0281954	0.999912	-4.244665
C5orf32	2.7448896	171.79223	2.7478808	0.0065548	0.999912	-4.004775
RGL4	2.7429479	195.21975	2.123608	0.03495	0.999912	-4.279201
S100A8	2.7185857	269.6452	2.6612998	0.0084258	0.999912	-4.046553
FLJ22662	2.707479	206.04185	2.3778181	0.0183718	0.999912	-4.175065
CD93	2.6862633	141.59103	2.469813	0.0143685	0.999912	-4.134769
LOC10013C	2.682069	113.63214	3.4934408	0.0005887	0.999912	-3.598919
REPS2	2.668879	143.69335	2.7028587	0.0074747	0.999912	-4.026645
FBXL13	2.633037	121.20068	2.4238707	0.0162589	0.999912	-4.155064
KCNJ15	2.6239344	181.81028	1.9798765	0.0491091	0.999912	-4.333295
CBS	2.6198311	114.64984	2.1431564	0.0333264	0.999912	-4.271574
TSEN34	2.5850978	209.50608	3.0585456	0.0025333	0.999912	-3.845471
HCG27	2.5760506	135.22833	2.4541921	0.0149881	0.999912	-4.141708
DUSP13	2.5599996	111.92135	2.3040642	0.0222615	0.999912	-4.206376
ALOX5AP	2.5334654	204.21685	2.123759	0.0349372	0.999912	-4.279142
LOC100132	2.5289225	124.32939	2.5376433	0.0119339	0.999912	-4.104186
HAL	2.5263009	121.66079	2.2810466	0.0236149	0.999912	-4.215966
CMTM2	2.5230558	180.71295	2.2087449	0.0283449	0.999912	-4.245518
KCNE3	2.5202525	156.46331	2.6874873	0.0078146	0.999912	-4.03404
GRB10	2.514342	125.39896	2.066737	0.040066	0.999912	-4.301023
FAM53C	2.5065546	144.06386	2.9247975	0.0038508	0.999912	-3.915832
GNG10	2.4725388	195.99523	2.0128008	0.0454963	0.999912	-4.321213
LIN7A	2.4634168	134.16495	2.6891245	0.0077778	0.999912	-4.033254
PISD	2.4513282	136.8552	2.1786444	0.0305446	0.999912	-4.257565
ROPN1L	2.4480884	131.62372	2.3795689	0.0182872	0.999912	-4.174311
NFIL3	2.4474039	181.32982	2.2899681	0.023082	0.999912	-4.212259
LOC389634	2.4450842	138.80496	2.6177812	0.0095374	0.999912	-4.067111
SORL1	2.4386528	248.64237	2.2266972	0.0270995	0.999912	-4.238261
TACC3	2.4299007	147.81865	2.830181	0.005134	0.999912	-3.963992
C3orf34	2.4225583	140.73634	2.1415155	0.0334601	0.999912	-4.272217
PYGL	2.4200151	186.88914	2.4291231	0.0160322	0.999912	-4.152761
FPR2	2.4189177	170.22003	2.1063918	0.0364359	0.999912	-4.285865
SLC19A1	2.4086249	150.01453	3.0843292	0.002333	0.999912	-3.831605
F5	2.397385	144.27382	2.2219754	0.0274224	0.999912	-4.240175
PREX1	2.3801027	216.21556	2.1810963	0.03036	0.999912	-4.256589

PLAUR	2.3790149	144.5714	2.6142341	0.0096336	0.999912	-4.068773
LY96	2.3762399	180.69724	2.3465859	0.0199391	0.999912	-4.188433
SLC16A5	2.3729554	191.33481	2.7357443	0.0067921	0.999912	-4.010702
S1PR4	2.3492432	239.68854	2.733258	0.0068417	0.999912	-4.011913
AQP9	2.3437631	215.37064	2.2456076	0.0258394	0.999912	-4.230559
NCF2	2.3412963	235.56894	2.3354822	0.020524	0.999912	-4.193147
KIAA0329	2.3224347	173.01069	2.5201879	0.0125224	0.999912	-4.112126
BCL2A1	2.3109303	182.89996	2.3292942	0.0208565	0.999912	-4.195765
MEGF9	2.2958098	163.25304	2.4257342	0.0161782	0.999912	-4.154247
MOSC1	2.293102	113.80254	2.1475078	0.0329739	0.999912	-4.269868
MPZL3	2.2896508	182.60106	2.2203102	0.027537	0.999912	-4.240849
RBP7	2.2867226	158.13093	2.1099941	0.0361206	0.999912	-4.284474
DIRC2	2.2849748	130.01231	3.0384774	0.0027	0.999912	-3.856197
CEBPB	2.2806662	218.66701	2.3154042	0.02162	0.999912	-4.20162
OSM	2.267555	131.5833	2.0907397	0.0378335	0.999912	-4.291879
SAP30	2.2671298	142.13301	2.636768	0.0090372	0.999912	-4.058178
CFLAR	2.2643228	150.6014	2.3971326	0.0174578	0.999912	-4.166718
TMEM184I	2.2601558	195.67472	2.2810141	0.0236169	0.999912	-4.215979
NCF4	2.2570304	165.56107	2.1406042	0.0335346	0.999912	-4.272574
B3GNT8	2.2568015	185.68126	2.2400443	0.0262047	0.999912	-4.232831
TREML2	2.2557878	161.92501	2.3486971	0.0198296	0.999912	-4.187534
BST1	2.2514384	168.26722	2.249492	0.025587	0.999912	-4.22897
SULT1B1	2.243544	122.1369	2.0712291	0.0396398	0.999912	-4.299319
LOC100132	2.2308863	104.41131	3.7302285	0.0002501	0.999912	-3.453829
LRRC4	2.2298153	119.30352	2.344338	0.0200563	0.999912	-4.189389
TBC1D14	2.2280413	164.20563	2.8918879	0.0042594	0.999912	-3.932736
H3F3B	2.2249065	226.07916	2.7425019	0.006659	0.999912	-4.007405
GABARAP	2.2201828	155.73049	3.075956	0.0023963	0.999912	-3.836118
TSHZ3	2.2178158	135.99598	2.1086955	0.036234	0.999912	-4.284976
CREB5	2.2173127	161.85507	2.1217024	0.0351119	0.999912	-4.279941
LASP1	2.2074404	173.65934	2.0949405	0.037454	0.999912	-4.290269
LOC49719C	2.2051403	134.71107	1.9899002	0.0479843	0.999912	-4.329636
SLC22A4	2.2022899	130.97865	2.0590384	0.0408055	0.999912	-4.303935
HIST1H2AI	2.189708	151.99858	2.5084417	0.0129328	0.999912	-4.117442
NACC2	2.1736218	150.48081	2.2346734	0.0265616	0.999912	-4.23502
FRAT2	2.1732111	211.22914	2.36763	0.0188707	0.999912	-4.179443
SKAP2	2.1728184	155.35839	2.1740787	0.0308908	0.999912	-4.259379
tcag7.907	2.166934	181.82288	2.5924823	0.0102426	0.999912	-4.078925
STX3	2.1642036	142.15463	2.587377	0.0103904	0.999912	-4.081297
SEPX1	2.164039	196.49671	2.1009463	0.036917	0.999912	-4.287962
LOC131691	2.1630782	173.361	2.9028053	0.0041197	0.999912	-3.927146
FADD	2.1610001	145.79542	2.7509978	0.0064951	0.999912	-4.00325
SEC14L1	2.1562826	158.54394	2.2570663	0.025101	0.999912	-4.225863

SLC25A44	2.149101	166.10376	2.944429	0.0036245	0.999912	-3.905671
LOC131055	2.1490152	178.06818	2.5734053	0.0108049	0.999912	-4.087766
LRRK2	2.1486131	172.90013	2.3415665	0.0202017	0.999912	-4.190566
IL13RA1	2.1469905	165.8709	2.1725984	0.0310038	0.999912	-4.259967
SVIL	2.1462115	136.49258	2.6116069	0.0097054	0.999912	-4.070003
ERO1L	2.1448386	126.99917	2.626449	0.009306	0.999912	-4.06304
C14orf94	2.1327056	125.03554	2.0382724	0.0428585	0.999912	-4.31174
S100A6	2.1309542	252.80009	2.0236393	0.0443575	0.999912	-4.317196
HIST1H3G	2.1302388	168.8455	2.3137317	0.0217136	0.999912	-4.202323
ISLR2	2.1085174	104.81237	2.1676007	0.0313879	0.999912	-4.261947
OSBPL2	2.1080101	147.97789	2.3366376	0.0204625	0.999912	-4.192657
MTMR3	2.0894577	173.30423	2.9543321	0.003515	0.999912	-3.900523
LILRB3	2.0874804	210.5375	2.0949181	0.037456	0.999912	-4.290278
C2orf39	2.0840478	108.20147	2.3247525	0.0211035	0.999912	-4.197683
SIRPA	2.0802297	136.3945	2.7265976	0.0069761	0.999912	-4.015153
LOC100128	2.0684208	143.5348	2.2159869	0.0278366	0.999912	-4.242597
KIAA1257	2.0648923	125.18964	2.0878838	0.0380935	0.999912	-4.292972
MCTP2	2.0586199	136.54147	2.3881175	0.0178793	0.999912	-4.170621
SLC22A15	2.0529432	142.46952	2.7452803	0.006605	0.999912	-4.006047
MNDA	2.0527481	233.43496	2.4451323	0.0153583	0.999912	-4.145714
MDGA1	2.0464704	101.58178	2.2228273	0.0273639	0.999912	-4.23983
IRAK3	2.0458303	129.2961	2.2636021	0.0246881	0.999912	-4.223175
PLXNC1	2.0390572	130.69418	2.4712041	0.0143144	0.999912	-4.134149
TMEM140	2.0233997	148.78378	2.0317192	0.0435244	0.999912	-4.314188
RASSF2	2.0212172	220.17527	2.0946597	0.0374793	0.999912	-4.290377
NRBF2	2.0192826	134.41542	2.3627914	0.0191118	0.999912	-4.181516
DOK3	2.0183598	168.33797	2.9179391	0.0039329	0.999912	-3.919368
AOAH	2.00678	144.66594	2.6957954	0.0076292	0.999912	-4.030048
LIMK2	2.0042234	158.15861	2.0434849	0.0423351	0.999912	-4.309788
PPP4R1	1.9957235	122.53634	2.5483796	0.0115844	0.999912	-4.099278
HIST1H4H	1.9840673	125.5434	2.1312898	0.0343041	0.999912	-4.276212
MAPK14	1.9815315	167.66898	2.2564476	0.0251404	0.999912	-4.226117
EFHD2	1.9753247	204.64981	3.0076249	0.0029761	0.999912	-3.872571
SLA	1.9730348	178.90348	2.1970924	0.0291795	0.999912	-4.2502
SYTL3	1.9695622	151.00661	2.6512058	0.0086727	0.999912	-4.051348
DHRS7	1.96275	171.86101	2.448728	0.0152104	0.999912	-4.144125
HIST1H2AI	1.958516	187.65737	2.3901067	0.0177855	0.999912	-4.169761
SMARCD3	1.9566702	170.65534	2.2934362	0.0228777	0.999912	-4.210815
SDCBP	1.9565899	165.50119	2.112083	0.0359388	0.999912	-4.283667
CSGALNA	1.9563681	125.23839	2.1517399	0.0326342	0.999912	-4.268205
CPAMD8	1.9561003	135.07383	2.1353526	0.0339666	0.999912	-4.274627
AOC3	1.9542042	121.43187	2.0838393	0.0384642	0.999912	-4.294518
C20orf24	1.9534513	193.37423	2.6058537	0.0098643	0.999912	-4.072693

UBN1	1.9515765	168.53632	2.1803745	0.0304142	0.999912	-4.256877
SQRDL	1.9509269	219.2459	2.4894758	0.0136209	0.999912	-4.125979
HIST1H3C	1.9464586	206.94407	2.0247556	0.0442416	0.999912	-4.316781
NUMB	1.9454073	168.36853	2.6543169	0.0085959	0.999912	-4.049872
TPD52L2	1.9452725	121.57911	2.1707145	0.0311481	0.999912	-4.260714
RAB27A	1.9407414	143.29682	2.7301877	0.0069033	0.999912	-4.013407
MGC23284	1.9387913	119.27244	2.4277022	0.0160933	0.999912	-4.153384
VNN3	1.9247953	122.10596	2.0124724	0.0455312	0.999912	-4.321335
OSBPL6	1.9160133	101.34165	3.1462482	0.0019101	0.999912	-3.797909
LOC440093	1.8999612	237.04538	2.0937879	0.0375578	0.999912	-4.290711
LOC729313	1.8932243	191.74703	2.330137	0.0208109	0.999912	-4.195409
LPGAT1	1.88777	154.34387	2.5413753	0.0118114	0.999912	-4.102482
CCDC49	1.8847053	127.62182	3.1276971	0.0020287	0.999912	-3.808062
LOC728368	1.8804862	156.74917	2.8678488	0.0045825	0.999912	-3.944981
IL18R1	1.8744018	112.54152	2.7395888	0.0067161	0.999912	-4.008827
NADK	1.8728195	160.80562	2.0381558	0.0428703	0.999912	-4.311784
AGPAT9	1.8707326	149.58721	2.0876767	0.0381124	0.999912	-4.293052
HIST1H3B	1.8615183	152.14368	2.7669053	0.0061979	0.999912	-3.99544
SCARF1	1.8579738	125.72616	2.0508835	0.0416015	0.999912	-4.307009
GAGE7	1.8573565	96.218763	2.3600497	0.0192496	0.999912	-4.182689
SLC37A3	1.8557623	120.5329	2.554886	0.011377	0.999912	-4.096295
GLIPR2	1.8546114	223.39836	2.0904275	0.0378619	0.999912	-4.291999
HIST2H3A	1.8324167	177.26806	2.3513036	0.0196951	0.999912	-4.186424
ISG20	1.8307196	232.46586	2.188002	0.0298454	0.999912	-4.253836
HIST1H3D	1.8280006	137.89145	2.2831884	0.023486	0.999912	-4.215077
NAGK	1.8249504	180.90524	2.0703401	0.0397239	0.999912	-4.299657
C20orf106	1.8207818	138.54336	2.6057632	0.0098668	0.999912	-4.072735
PHC2	1.8188112	150.25898	2.3457774	0.0199812	0.999912	-4.188777
MAK	1.8186926	116.52406	2.1166524	0.035544	0.999912	-4.281899
LOC440731	1.8182014	130.50368	2.16187	0.0318334	0.999912	-4.264213
C1RL	1.8136811	129.44456	2.2804569	0.0236505	0.999912	-4.21621
MSL1	1.8129343	151.76825	2.002775	0.0465718	0.999912	-4.324912
LTB4R	1.808103	126.51369	2.040563	0.0426278	0.999912	-4.310883
INHBB	1.8058211	101.70681	2.5300252	0.0121877	0.999912	-4.107657
C1orf187	1.8050775	111.65647	2.1260835	0.0347407	0.999912	-4.278239
WDFY3	1.8032075	121.06898	2.3885801	0.0178574	0.999912	-4.170421
CIR	1.8011236	181.33325	2.133711	0.0341026	0.999912	-4.275267
TYROBP	1.7960848	252.58312	2.2510875	0.0254839	0.999912	-4.228316
FLJ43093	1.7959976	103.38026	2.7170704	0.0071725	0.999912	-4.019776
MMP1	1.7915793	99.530627	2.2052007	0.0285965	0.999912	-4.246944
POM121L4	1.7866395	108.44579	2.4099493	0.0168737	0.999912	-4.161146
STX11	1.7810506	138.98054	2.0971562	0.0372551	0.999912	-4.289419
HN1	1.7803147	186.20992	2.1528765	0.0325435	0.999912	-4.267758

B9D2	1.7773223	167.22496	2.5895384	0.0103276	0.999912	-4.080293
RILPL2	1.7755595	204.2119	2.4356742	0.0157533	0.999912	-4.149882
CREBBP	1.7681623	137.85024	2.2751621	0.0239723	0.999912	-4.218403
NHS	1.7641027	115.95139	2.1028277	0.0367502	0.999912	-4.287238
FLJ36031	1.7552862	121.18204	2.5446557	0.0117046	0.999912	-4.100982
HIST1H2B(	1.7531576	130.89457	2.5672022	0.0109936	0.999912	-4.090629
HIST2H4B	1.750479	123.08231	3.1425791	0.0019331	0.999912	-3.799921
GGTLC1	1.7463505	130.72756	2.201447	0.0288652	0.999912	-4.248453
PPP1R3B	1.7446783	146.73987	2.0819736	0.0386362	0.999912	-4.29523
ENTPD1	1.7445797	118.67882	2.7627906	0.0062736	0.999912	-3.997464
PSMB9	1.7401462	196.43146	2.0695166	0.0398018	0.999912	-4.299969
LOC64495(	1.7295649	234.73574	2.4131002	0.0167328	0.999912	-4.159772
LOC391532	1.7226061	159.60033	2.6404196	0.0089437	0.999912	-4.056454
CYP4F8	1.7209472	121.72466	2.2663085	0.0245188	0.999912	-4.22206
CG012	1.7099871	113.75081	2.1072575	0.0363599	0.999912	-4.285531
SLC40A1	1.7099757	158.8084	1.9800567	0.0490887	0.999912	-4.333229
HIST3H2A	1.7086106	170.79774	2.0650168	0.0402302	0.999912	-4.301675
NLRP3	1.6991309	164.90869	2.0729914	0.0394737	0.999912	-4.29865
LOC202227	1.6890453	162.96861	2.336797	0.020454	0.999912	-4.19259
SLC2A3	1.6846427	122.4754	2.2358771	0.0264813	0.999912	-4.23453
BRI3	1.6800858	231.45842	2.0536334	0.0413316	0.999912	-4.305974
KLF6	1.6800303	201.21085	2.1411849	0.0334871	0.999912	-4.272346
SLC39A1	1.6717145	122.0206	2.2118484	0.0281261	0.999912	-4.244267
LOC10013(	1.671036	128.57336	1.9884202	0.048149	0.999912	-4.330178
GABARAP.	1.6690105	109.69246	2.599282	0.0100486	0.999912	-4.075759
LST1	1.6665532	221.65662	1.9882311	0.0481701	0.999912	-4.330247
UBXN2B	1.6626376	131.48313	2.0580951	0.0408969	0.999912	-4.304291
CASP4	1.6473766	225.64524	2.242417	0.0260484	0.999912	-4.231863
NUDT16	1.6472937	141.54189	2.3947503	0.0175683	0.999912	-4.167751
HIST1H4F	1.6418856	112.507	2.8799279	0.0044175	0.999912	-3.938839
UBR2	1.6407821	165.2873	3.0007201	0.0030414	0.999912	-3.876216
F11R	1.6398387	158.44864	2.402346	0.0172181	0.999912	-4.164455
MAN2A2	1.637712	171.9869	2.9394842	0.0036803	0.999912	-3.908235
ELL	1.6374235	146.48956	2.2256182	0.027173	0.999912	-4.238699
FAM49A	1.6337791	153.99887	2.0706185	0.0396975	0.999912	-4.299551
HIST1H2BI	1.6273752	170.29491	2.4887361	0.0136484	0.999912	-4.126311
PSTPIP1	1.6248514	192.79482	2.0733321	0.0394417	0.999912	-4.29852
OBFC2A	1.6183921	147.21623	2.2295994	0.0269027	0.999912	-4.237083
NLRX1	1.6181783	166.05459	2.1235807	0.0349523	0.999912	-4.279212
PIGX	1.6160292	129.47645	2.4374152	0.0156799	0.999912	-4.149116
ATP6V0E1	1.6088024	149.82269	2.3539867	0.0195574	0.999912	-4.185279
PCNX	1.6083592	143.17382	1.9785784	0.0492563	0.999912	-4.333767
C1orf183	1.6032224	155.89258	1.9852567	0.0485026	0.999912	-4.331333

CR1	1.5961203	113.53878	1.9739159	0.0497884	0.999912	-4.335462
B3GNTL1	1.596074	116.88157	2.3890972	0.017833	0.999912	-4.170198
ACOX1	1.5897066	129.55204	2.5311855	0.0121487	0.999912	-4.107129
BEND7	1.5886228	100.39686	2.7771792	0.0060126	0.999912	-3.990376
SRA1	1.5884313	137.52903	2.4676248	0.0144539	0.999912	-4.135743
GYG1	1.5843997	124.42737	2.2814534	0.0235904	0.999912	-4.215797
HIST1H3H	1.5711982	130.71547	2.0503975	0.0416494	0.999912	-4.307192
LAMP2	1.5691238	152.63481	2.0885366	0.0380339	0.999912	-4.292723
HIST1H2B(	1.5643095	162.24451	2.4234037	0.0162792	0.999912	-4.155268
RRAGD	1.5599376	119.31372	2.0410759	0.0425763	0.999912	-4.310691
SLC39A5	1.5592964	102.16793	2.7473464	0.0065651	0.999912	-4.005037
RAF1	1.5576931	168.17605	2.168846	0.0312918	0.999912	-4.261454
TUBA4A	1.5552513	196.78479	2.1724433	0.0310156	0.999912	-4.260028
CDC123	1.5536345	152.18024	2.5511785	0.0114948	0.999912	-4.097995
HIST1H4I	1.5536189	114.57406	2.8417019	0.0049593	0.999912	-3.9582
LOC391769	1.5453881	255.53348	2.1201634	0.0352431	0.999912	-4.280538
RNF12	1.5432685	123.13131	2.3264488	0.0210109	0.999912	-4.196967
CYB5R4	1.5337177	163.31317	2.1748291	0.0308337	0.999912	-4.259081
CD58	1.5334573	142.03132	2.2875283	0.0232267	0.999912	-4.213274
CTBP2	1.5328045	150.72316	2.2097771	0.0282719	0.999912	-4.245102
HIST2H2BI	1.5297637	127.95101	2.542801	0.0117649	0.999912	-4.10183
KIF1B	1.5249243	114.28303	2.5442253	0.0117185	0.999912	-4.101179
ZNF516	1.5174964	128.15526	2.7843351	0.0058865	0.999912	-3.986838
ERLIN1	1.5173366	123.59594	2.3815913	0.01819	0.999912	-4.173439
PTPRJ	1.5153444	138.2315	2.4919373	0.0135298	0.999912	-4.124875
ATXN1	1.513224	121.05745	2.5686887	0.0109481	0.999912	-4.089944
QSOX1	1.5126494	178.68643	2.1729582	0.0309763	0.999912	-4.259824
XKR8	1.5115658	192.70287	2.1140889	0.035765	0.999912	-4.282892
SEMA4D	1.5094999	130.29184	2.2523992	0.0253995	0.999912	-4.227778
MBOAT2	1.5052346	135.62955	1.9939472	0.0475365	0.999912	-4.328154
HIST1H2BI	1.5046371	174.13826	2.4857804	0.0137587	0.999912	-4.127636
H3F3A	1.5041522	256.52344	2.1233223	0.0349743	0.999912	-4.279312
TPM4	1.502697	157.59229	2.2520864	0.0254196	0.999912	-4.227907
EVL	-1.500018	157.01092	-2.321368	0.0212892	0.999912	-4.19911
DDX39	-1.525617	171.11156	-2.082228	0.0386127	0.999912	-4.295133
CHI3L2	-1.582564	107.50457	-1.99054	0.0479133	0.999912	-4.329402
KIAA1303	-1.589863	132.68528	-2.063252	0.0403993	0.999912	-4.302342
DDX26B	-1.595825	124.659	-2.020546	0.04468	0.999912	-4.318344
PLAG1	-1.59714	112.79018	-2.417185	0.0165517	0.999912	-4.157989
AMBRA1	-1.633091	129.71932	-2.247988	0.0256845	0.999912	-4.229585
CCDC14	-1.645824	141.81251	-2.034628	0.0432277	0.999912	-4.313102
GPR18	-1.657734	125.11782	-2.160516	0.0319394	0.999912	-4.264748
PLCXD1	-1.660981	115.98941	-2.436504	0.0157183	0.999912	-4.149517

CD22	-1.664084	108.12429	-2.038717	0.0428137	0.999912	-4.311574
CHCHD10	-1.684604	140.07109	-2.011426	0.0456425	0.999912	-4.321721
RRAS	-1.703543	155.56904	-2.313772	0.0217113	0.999912	-4.202306
APOBEC3(	-1.714176	121.33851	-2.092695	0.0376565	0.999912	-4.291131
EPS8	-1.714311	101.76246	-2.56448	0.0110773	0.999912	-4.091883
RPL39	-1.723277	222.40039	-2.392619	0.0176677	0.999912	-4.168674
FUBP1	-1.737594	119.9873	-2.153118	0.0325243	0.999912	-4.267663
KCNG1	-1.738066	116.59216	-2.138158	0.0337352	0.999912	-4.27353
MAFB	-1.764785	160.98394	-2.094624	0.0374825	0.999912	-4.290391
CCT2	-1.772335	180.6774	-2.655674	0.0085627	0.999912	-4.049228
HLA-DPA1	-1.793072	170.89676	-2.251488	0.0254581	0.999912	-4.228152
LOC23117	-1.899393	204.13468	-2.078302	0.0389767	0.999912	-4.29663
DDX51	-1.912252	134.59106	-2.600156	0.0100239	0.999912	-4.075352
ABLIM1	-1.93091	185.83232	-1.989094	0.0480739	0.999912	-4.329931
TCF4	-1.950902	122.83338	-2.314356	0.0216787	0.999912	-4.202061
TPPP3	-1.99138	112.35786	-2.096566	0.037308	0.999912	-4.289645
FAM113B	-2.014753	150.09299	-2.213485	0.0280113	0.999912	-4.243607
ARGLU1	-2.037845	147.90369	-1.985999	0.0484195	0.999912	-4.331062
PTPRK	-2.049606	110.28973	-2.140694	0.0335273	0.999912	-4.272539
HSPB1	-2.112043	152.71462	-2.19536	0.0293055	0.999912	-4.250894
FAIM3	-2.199746	136.04558	-2.526634	0.0123022	0.999912	-4.1092
BACH2	-2.216588	119.93481	-2.689486	0.0077696	0.999912	-4.03308
SLC25A32	-2.223059	128.63018	-2.49763	0.0133212	0.999912	-4.122316
KCNH8	-2.267326	100.75013	-2.725447	0.0069995	0.999912	-4.015712
ZNF550	-2.297329	132.61514	-2.121897	0.0350953	0.999912	-4.279865
KLRB1	-2.315875	167.95845	-2.132579	0.0341966	0.999912	-4.275709
CD248	-2.371311	122.82688	-2.363132	0.0190947	0.999912	-4.18137
LOC440348	-2.412493	159.23117	-2.158573	0.0320922	0.999912	-4.265514
KLF12	-2.422635	146.74946	-2.502034	0.0131618	0.999912	-4.120333
L1TD1	-2.422859	98.641065	-3.346148	0.0009812	0.999912	-3.685388
IGHD	-2.428831	118.88863	-2.011634	0.0456204	0.999912	-4.321645
ZNF234	-2.432591	119.74351	-2.389476	0.0178152	0.999912	-4.170034
CD79B	-2.45804	125.31977	-2.128346	0.0345504	0.999912	-4.277358
CD83	-2.613465	126.875	-3.180099	0.0017101	0.999912	-3.779254
TARP	-2.735545	130.65015	-1.987808	0.0482172	0.999912	-4.330401
BLK	-2.886962	127.97103	-2.421484	0.0163629	0.999912	-4.156109
HLA-DPB1	-3.174824	210.76943	-3.063602	0.0024928	0.999912	-3.842759
C7orf16	-3.228526	108.61817	-2.522165	0.0124545	0.999912	-4.111229
KRT77	-3.54322	97.188719	-2.540231	0.0118488	0.999912	-4.103005
CDKN1C	-3.775888	174.31661	-2.408557	0.0169363	0.999912	-4.161753
HBZ	-9.748103	136.42117	-2.86204	0.0046639	0.999912	-3.947926
