# Supplementary material for: Comprehensive Analysis Based on Genes Associated With Cuproptosis, Ferroptosis, and Pyroptosis for the Prediction of Diagnosis and Therapies in Coronary Artery Disease
Source: Cardiovasc Ther. 2025 Mar 15;2025:9106621. doi: 10.1155/cdr/9106621 (PMC11929595; doi:10.1155/cdr/9106621)
Supplement: Supporting Information 4 — Table S3: The differentially expressed genes between CAD and control samples in GSE42148. [file 9106621.f4.pdf]

Supplementary Table 3. The differentially expressed genes between CAD and control samples in GSE42148

DEG	logFC	AveExpr	t	P.Value	adj.P.Val	B
EGR3	12.381845	15.758342	5.1019007	2.76E-05	0.0638678	2.4237394
LOC286058	9.7738993	14.07805	4.1042562	0.0003709	0.1057281	0.176851
G0S2	8.3473678	34.6192	3.0435828	0.0053868	0.2266983	-2.13851
IL8	7.9290028	29.029058	3.6872544	0.0010835	0.1399536	-0.753598
UNQ9368	7.2037147	15.917067	3.577915	0.0014303	0.1491954	-0.994306
ID1	7.1583469	19.475983	3.5096681	0.0016995	0.1541747	-1.143626
EGR2	6.4622629	23.395283	3.5991921	0.0013553	0.1482829	-0.947602
A_33_P327(	6.4601874	16.73105	3.5564802	0.00151	0.1515078	-1.041285
EGR1	6.1672741	30.042425	3.1463689	0.0041939	0.2139793	-1.923461
ENST00000(	5.9202769	23.092417	2.7787742	0.0101334	0.2779846	-2.678249
AREG	5.8371058	18.977778	2.4257205	0.0227232	0.3678645	-3.358807
ENST00000(	5.6699804	17.179167	4.4326497	0.000158	0.0736132	0.9172357
A_33_P335(	5.4275944	15.270383	3.4829491	0.0018178	0.1594252	-1.201874
ENST00000(	5.4167273	13.174933	2.7310986	0.0113292	0.2924256	-2.772965
ENST00000(	5.394151	10.89445	6.0418827	2.46E-06	0.0214714	4.482526
A_33_P327(	5.2863021	18.76105	3.7495865	0.0009242	0.1345858	-0.615648
ENST00000(	5.2842266	16.729417	2.9952348	0.0060544	0.2358908	-2.238659
CTAG1A	5.2317217	11.314158	3.1773297	0.0038873	0.2089565	-1.858147
ENST00000(	5.1765343	11.566683	4.6478356	9.01E-05	0.064665	1.4031144
TTTY7	5.096372	8.5163167	2.8191724	0.0092143	0.2690606	-2.597369
FOSB	5.0800028	24.20425	3.3747029	0.0023844	0.1706805	-1.436525
ENST00000(	4.8107329	15.77905	5.0392147	3.25E-05	0.0638678	2.2834896
ENST00000(	4.810028	13.28005	4.8979424	4.69E-05	0.064665	1.9665236
ENST00000(	4.739558	10.934933	3.9638732	0.0005331	0.1164353	-0.138112
MMP8	4.5972769	12.934192	3.4907122	0.0017826	0.1574654	-1.184963
MS4A3	4.5840895	18.973267	3.5973875	0.0013615	0.1482829	-0.951566
A_33_P321:	4.5684056	21.067317	4.7516129	6.87E-05	0.064665	1.6371605
A_33_P331(	4.5584587	18.553383	4.2363927	0.0002633	0.0902041	0.4743418
CXCL1	4.550372	31.065767	3.9456874	0.0005587	0.1164353	-0.178808
OLFM4	4.5388685	27.503408	2.2491869	0.0334301	0.4131238	-3.678883
NR4A2	4.5352886	17.470639	3.2016601	0.0036617	0.2061571	-1.806655
ENST00000(	4.4595776	18.56015	4.1620882	0.0003193	0.0995993	0.3069538
LOC100129	4.4196923	16.1364	3.5206871	0.0016529	0.1530728	-1.119569
ENST00000(	4.400621	15.309233	4.9396256	4.21E-05	0.064665	2.0601637
HUS1B	4.3477734	13.083117	3.3165847	0.0027558	0.1859295	-1.561564
lincRNA:ch	4.3390014	7.9665833	2.1742176	0.0392287	0.4319479	-3.810181
ENST00000(	4.3004476	10.6505	3.2952142	0.0029059	0.1891663	-1.607363
NFKBID	4.2588587	19.8975	3.0809776	0.0049194	0.2225409	-2.0606
RGS1	4.2377608	26.484908	2.5766085	0.0161782	0.3251357	-3.074068
PTGS2	4.2175382	29.749522	2.4790994	0.0201698	0.3519698	-3.259194

A_33_P339.	4.1937538	13.029217	2.2644633	0.0323484	0.4084332	-3.65178
LOC100129	4.1662434	27.116133	3.6595538	0.0011627	0.1429549	-0.814741
SFN	4.1458406	23.9001	4.5104044	0.000129	0.0677403	1.0928365
GALR3	4.1034881	39.96545	3.5385159	0.0015802	0.1529792	-1.080601
lincRNA:ch	4.0848671	12.7148	3.4437228	0.0020061	0.1624754	-1.287159
KLHL15	4.0470573	9.7648833	4.0558645	0.0004204	0.1096022	0.0681296
ENST00000	4.0313343	15.700767	3.7619194	0.0008955	0.1345828	-0.588296
ENST00000	4.0309231	16.824617	4.2800067	0.0002351	0.0890075	0.5726877
OLR1	4.0233748	14.151492	2.1897132	0.0379607	0.4270606	-3.78328
A_33_P334	3.9684224	11.303017	4.5223182	0.000125	0.0677403	1.1197422
PCOLCE2	3.9433986	14.918517	2.7045607	0.0120511	0.2962939	-2.825331
lincRNA:ch	3.9423413	16.708417	6.0459295	2.43E-06	0.0214714	4.4911658
A_33_P336	3.9067636	36.547	3.2053536	0.0036285	0.2061571	-1.798825
ENST00000	3.9058434	23.038283	3.5605683	0.0014945	0.1515078	-1.032331
NCAPG2	3.8951916	16.29775	2.4694862	0.0206091	0.3554645	-3.277227
CEACAM6	3.8892587	18.5143	2.399936	0.0240604	0.3757321	-3.406469
FCAR	3.8643671	23.879363	3.7391947	0.0009491	0.1364247	-0.63868
ENST00000	3.8300671	8.0785833	3.1898867	0.0037692	0.2065576	-1.83159
LOC730167	3.8294601	10.0576	3.5477282	0.0015438	0.1515078	-1.060446
A_24_P926	3.8271692	12.91465	3.2178252	0.0035188	0.2056835	-1.772364
KRTAP9-9	3.8243105	14.300883	3.0704728	0.0050466	0.2240985	-2.082525
ENST00000	3.8216476	12.89715	2.8836916	0.0079081	0.2570639	-2.467058
MAFF	3.8165743	22.813478	3.0708085	0.0050425	0.2240985	-2.081825
DGCR11	3.7974657	19.309033	7.3349808	1.01E-07	0.0035446	7.1180033
DMRTC2	3.7918657	11.018	3.3955934	0.0022631	0.1688885	-1.391413
FAM153C	3.7860112	13.526683	2.8718679	0.0081335	0.2579482	-2.491041
LOC100127	3.7761427	8.8114833	3.7785475	0.0008582	0.1329829	-0.55139
ENST00000	3.7745566	14.789133	3.4593087	0.0019291	0.1617964	-1.253306
TP53INP2	3.7485538	17.618067	3.5910815	0.0013834	0.1487994	-0.965413
ENST00000	3.7385678	27.866767	3.040531	0.0054268	0.2266983	-2.144851
LOC100128	3.736551	15.968983	3.4160778	0.00215	0.1687148	-1.347095
PER1	3.733565	28.595875	2.9531069	0.0066997	0.2412766	-2.325367
BQ706149	3.7236378	22.387225	4.0196031	0.0004617	0.1111414	-0.01324
lincRNA:ch	3.7175385	10.055267	3.2479523	0.0032667	0.1995927	-1.708292
COL17A1	3.7095888	16.086233	2.1776344	0.0389458	0.4316899	-3.80426
ENST00000	3.703793	8.3395667	3.2640606	0.0031391	0.1955602	-1.673948
lincRNA:ch	3.6840951	11.948533	3.0195543	0.0057093	0.2306829	-2.188366
ENST00000	3.6689594	19.734517	4.662266	8.68E-05	0.064665	1.4356758
lincRNA:ch	3.6574266	27.445833	4.3047179	0.0002204	0.0888702	0.6284344
EMR4P	3.6559385	12.5475	2.9923603	0.0060964	0.2367386	-2.244592
lincRNA:ch	3.6549986	27.8327	3.7572031	0.0009064	0.1345828	-0.598758
A_33_P330	3.6417231	12.775	2.4521505	0.0214238	0.360102	-3.309642
lincRNA:ch	3.6356531	11.397167	4.6959232	7.95E-05	0.064665	1.5116021

LOC100133	3.6345175	13.921133	2.5634323	0.0166709	0.3294068	-3.099315
lincRNA:ch	3.6250797	17.230967	2.4641532	0.0208567	0.3573357	-3.287213
ENST00000	3.6185986	22.674983	2.515723	0.0185742	0.3415817	-3.190132
USP32	3.6147413	22.461367	3.5361892	0.0015895	0.1530328	-1.08569
DEFA4	3.6073007	34.4057	2.5254414	0.0181708	0.3388496	-3.17171
C5orf23	3.6014266	12.5643	2.9393722	0.0069239	0.2451721	-2.353521
CSRNP1	3.5804853	27.027175	3.2089587	0.0035965	0.2061571	-1.79118
ENST00000	3.5682378	19.94825	4.0355417	0.0004431	0.1111414	0.0225147
A_33_P333	3.5651245	7.7294	4.0284226	0.0004513	0.1111414	0.0065423
lincRNA:ch	3.5626965	16.239067	3.0866544	0.0048519	0.2225409	-2.04874
lincRNA:ch	3.5588065	10.475578	4.6541503	8.87E-05	0.064665	1.4173638
PRRT4	3.5345594	18.382117	3.5897861	0.001388	0.1487994	-0.968257
ENST00000	3.4870573	16.78915	3.2410958	0.0033225	0.200491	-1.722893
EIF5	3.4780993	25.173225	5.0135926	3.47E-05	0.0638678	2.22609
ENST00000	3.4677119	10.332817	4.9295046	4.32E-05	0.064665	2.0374354
lincRNA:ch	3.4655776	20.261733	4.733189	7.21E-05	0.064665	1.5956326
lincRNA:ch	3.465558	19.26085	3.3402651	0.0025981	0.1784419	-1.510701
FAM105B	3.4518517	23.063717	4.0118649	0.000471	0.1111414	-0.030593
lincRNA:ch	3.4500699	13.594933	3.9445184	0.0005604	0.1164353	-0.181423
PAPD5	3.4479944	11.0397	3.3940261	0.002272	0.1688885	-1.394801
ENST00000	3.4444895	16.983983	4.5277347	0.0001233	0.0677403	1.1319741
ENST00000	3.4353063	10.7709	2.9795151	0.0062879	0.2375467	-2.271075
ENST00000	3.4323301	10.651433	3.7515276	0.0009196	0.1345858	-0.611344
CENPV	3.4276503	13.113917	2.9778618	0.006313	0.2377409	-2.27448
PTX3	3.4175351	24.267787	3.1585006	0.0040711	0.2111883	-1.897897
MGC16384	3.4061421	18.989324	2.6252163	0.0144754	0.3139876	-2.980326
PDZD3	3.3734909	12.529417	3.6119671	0.0013121	0.1474477	-0.919527
LOC388780	3.3490545	15.587017	3.2749618	0.0030555	0.1927588	-1.650673
LOC100131	3.3442378	16.424917	5.466423	1.07E-05	0.0416449	3.23312
TMEM196	3.3377566	8.0061333	2.6061153	0.0151235	0.3175738	-3.017275
lincRNA:ch	3.3362685	18.6648	4.2530211	0.0002521	0.0890075	0.51183
lincRNA:ch	3.3252839	11.03025	3.6261481	0.0012657	0.1468158	-0.888335
PLK3	3.3143189	36.368383	2.6162663	0.0147758	0.3165792	-2.997657
ENST00000	3.3133007	13.14565	3.055292	0.0052361	0.2245871	-2.114156
A_33_P331	3.3111273	14.2548	2.6726542	0.0129762	0.3029433	-2.887947
LTF	3.2806406	33.19365	2.3415963	0.0273568	0.3917302	-3.513179
lincRNA:ch	3.2766853	10.289417	3.868911	0.0006807	0.1239125	-0.350298
C15orf37	3.2695776	18.598767	4.5457961	0.0001176	0.0677403	1.172761
ENST00000	3.2622937	19.843367	3.959635	0.000539	0.1164353	-0.147599
lincRNA:ch	3.2608056	13.947033	5.2561533	1.85E-05	0.0564492	2.7676283
C9orf72	3.2552252	23.596883	3.5812629	0.0014183	0.1491954	-0.986962
ELANE	3.2419399	24.180742	2.289117	0.0306695	0.4022014	-3.607794
NUDT16P	3.219628	14.882583	2.9724675	0.0063954	0.2393071	-2.285584

STK35	3.2142629	15.93935	3.7951351	0.0008226	0.1315436	-0.514543
ATPAF1	3.1986965	11.6795	3.5178342	0.0016648	0.1530728	-1.125799
lincRNA:ch	3.1957986	11.366367	3.4509128	0.0019702	0.1624754	-1.271548
lincRNA:ch	3.1921762	19.32175	3.921144	0.0005952	0.1181849	-0.233687
TSPAN19	3.1919413	7.22715	2.6742553	0.0129283	0.3029433	-2.884814
UNQ3118	3.1896699	11.586283	2.5957529	0.0154864	0.3193262	-3.03726
LOC100129	3.1791944	7.6853	2.7855363	0.0099738	0.276697	-2.66475
ENST00000	3.178235	19.525217	2.4235028	0.0228355	0.3685575	-3.362918
GRM2	3.1743972	13.598083	2.7493016	0.0108578	0.2857438	-2.736897
lincRNA:ch	3.1607497	13.7592	5.224541	2.01E-05	0.0564492	2.697305
RAPGEFL1	3.1607497	10.948	2.7631476	0.0105116	0.2822202	-2.709383
LOC100128	3.1574601	21.1708	3.5362409	0.0015893	0.1530328	-1.085577
lincRNA:ch	3.1524867	18.439633	3.4026429	0.0022235	0.1688885	-1.376171
LOC100291	3.1358825	7.0585667	2.5527197	0.0170818	0.332813	-3.119789
C14orf49	3.1284811	16.972667	2.9659464	0.0064964	0.240257	-2.298997
LOC100132	3.1197483	13.5163	3.2036699	0.0036436	0.2061571	-1.802395
ZNF597	3.1167329	9.1410667	4.1308681	0.0003462	0.1052093	0.2366973
lincRNA:ch	3.1141091	8.4763	2.7936748	0.0097848	0.2751492	-2.648483
TRIB1	3.1138252	38.878292	4.9528957	4.07E-05	0.064665	2.0899549
LOC728114	3.0918462	6.47675	2.4747653	0.0203668	0.3537766	-3.267329
LOC148987	3.0804895	8.3084167	4.3768862	0.0001827	0.0811076	0.7913207
BEX1	3.0763972	19.3382	2.4341471	0.0223013	0.3659732	-3.343166
lincRNA:ch	3.0751049	19.8583	4.4031199	0.0001706	0.0774356	0.8505528
lincRNA:ch	3.071972	20.624567	4.5183158	0.0001264	0.0677403	1.1107034
ENST00000	3.0647469	15.797017	3.5428921	0.0015628	0.1517179	-1.071029
lincRNA:ch	3.0544867	31.37015	3.0778178	0.0049573	0.2225409	-2.067198
LOC441208	3.0539972	11.794067	2.8572371	0.0084209	0.2602147	-2.520655
CECR7	3.053449	13.051733	3.1230862	0.0044395	0.2177896	-1.972418
IRS2	3.0511287	33.600058	4.486714	0.0001372	0.0684648	1.0393338
EGR4	3.0495133	9.2685833	2.3605962	0.0262404	0.3875594	-3.4786
lincRNA:ch	3.0493762	19.642	4.8279017	5.63E-05	0.064665	1.8089868
A_33_P331	3.0416615	30.574367	3.4124739	0.0021695	0.1688685	-1.354898
ARG1	3.041531	31.188344	2.708352	0.0119454	0.2960758	-2.817866
A_33_P336	3.0342406	29.693183	3.0211558	0.0056872	0.2306829	-2.185048
LOC100129	3.0327133	11.068283	3.7378043	0.0009525	0.1364247	-0.641761
ENST00000	3.0303441	11.3246	4.3202584	0.0002117	0.0880737	0.6635007
lincRNA:ch	3.0293846	15.386117	3.4468556	0.0019904	0.1624754	-1.280358
C6orf146	3.029228	6.98985	2.7038423	0.0120712	0.2962939	-2.826745
lincRNA:ch	3.0115664	7.0474833	2.8981454	0.0076406	0.2548004	-2.43768
KIF13A	3.0036168	20.39485	4.0428369	0.0004348	0.1109165	0.0388859
lincRNA:ch	2.9892056	22.715117	3.7450841	0.0009349	0.1353853	-0.625628
lincRNA:ch	2.9848196	25.07365	2.7015678	0.0121352	0.2962939	-2.831221
KLF5	2.9834587	22.439258	2.8882191	0.0078234	0.2563428	-2.457863

ABCA13	2.9826657	14.746083	2.1106586	0.0448372	0.4502922	-3.919197
LOC727916	2.9809231	8.6118667	3.8535038	0.0007082	0.1271967	-0.384645
lincRNA:ch	2.9805315	9.4542	4.280168	0.000235	0.0890075	0.5730514
MAP7	2.9729865	16.714756	5.4992751	9.85E-06	0.0416449	3.305463
lincRNA:ch	2.9707608	10.818617	2.5964199	0.0154628	0.3193262	-3.035975
BPI	2.968607	23.41465	2.3547152	0.0265814	0.3883755	-3.489322
C17orf107	2.9666294	19.311833	2.8946653	0.0077042	0.2558378	-2.444759
LOC100128	2.9644168	12.421617	2.4477691	0.0216344	0.3614175	-3.317814
ENST00000	2.9495748	21.88445	3.0455021	0.0053618	0.2266983	-2.134521
ENST00000	2.9490853	17.277167	2.6101927	0.0149829	0.3172273	-3.0094
LOC100132	2.9474601	9.13745	3.625198	0.0012688	0.1468158	-0.890426
LOC100133	2.946814	13.5443	3.9076338	0.0006162	0.1203159	-0.263872
WDR93	2.9447776	22.225233	2.19203	0.0377743	0.4266764	-3.779247
lincRNA:ch	2.9382965	12.68645	2.1425952	0.0419351	0.4421393	-3.864689
CILP	2.9379832	11.552917	2.857848	0.0084087	0.2602147	-2.51942
lincRNA:ch	2.9327944	10.346233	3.4642619	0.0019052	0.1617143	-1.242538
ENST00000	2.929642	11.948417	3.0238412	0.0056504	0.2304636	-2.179484
ELF3	2.9214671	12.037025	3.5818081	0.0014163	0.1491954	-0.985766
ANKRD44	2.9207329	18.8587	3.874774	0.0006706	0.1233446	-0.337221
FOXJ2	2.9205371	20.014867	2.3808874	0.0250944	0.3823116	-3.441485
lincRNA:ch	2.9170126	9.7976667	3.2944593	0.0029114	0.1891663	-1.608979
FLJ44635	2.9105315	15.920683	2.9035628	0.0075425	0.2548004	-2.426651
ENST00000	2.905186	8.2091333	3.0791973	0.0049407	0.2225409	-2.064318
PPARG	2.8927845	10.377553	2.6613273	0.0133204	0.3051802	-2.910083
lincRNA:ch	2.888151	15.677433	4.6107642	9.93E-05	0.0671784	1.3194449
lincRNA:ch	2.8867608	24.826317	2.4657812	0.0207808	0.3570641	-3.284166
ORM1	2.8863594	30.202608	2.2038741	0.0368343	0.4230074	-3.758587
SLC5A9	2.8717818	11.759767	2.8478505	0.0086103	0.2621277	-2.539617
KCNK7	2.8678462	24.085017	3.4541445	0.0019543	0.1624754	-1.264528
ENST00000	2.8672587	17.427317	3.6830334	0.0010953	0.1399536	-0.762922
RP1-21O18	2.8586629	12.610733	2.0660036	0.0491958	0.4630302	-3.994482
BC021857	2.8562937	26.16705	2.9574148	0.0066308	0.2412766	-2.316525
lincRNA:ch	2.856235	22.68	3.7938111	0.0008254	0.1315436	-0.517485
EPCAM	2.8493818	9.2990333	3.0500327	0.0053033	0.2257538	-2.125099
SGK1	2.8448098	36.369375	5.3673654	1.39E-05	0.0484198	3.0143394
ENST00000	2.8446238	11.931383	2.5905355	0.0156721	0.3202106	-3.047306
FLJ31104	2.8443692	11.3071	3.1610906	0.0040453	0.2107	-1.892435
A_33_P341	2.8419217	9.7522833	4.0205476	0.0004606	0.1111414	-0.011122
NACC2	2.8320473	31.517138	4.6797304	8.29E-05	0.064665	1.4750768
lincRNA:ch	2.8275692	21.2296	3.5804071	0.0014213	0.1491954	-0.988839
A_33_P340	2.8256895	18.1384	3.7590531	0.0009021	0.1345828	-0.594654
MDN1	2.8226154	13.126517	2.7918775	0.0098262	0.2758365	-2.652077
DEFT1P2	2.8211664	12.58635	2.9532549	0.0066974	0.2412766	-2.325064

A_33_P325:	2.8143133	14.206383	2.4094868	0.0235569	0.372736	-3.38885
ARHGAP1'	2.8021734	8.5289167	3.6177411	0.001293	0.1469235	-0.906831
ProSAPiP1	2.7990797	11.93115	3.0967262	0.0047344	0.2225409	-2.027675
PMAIP1	2.7990699	29.738508	2.8100836	0.009414	0.2697407	-2.615614
ENST0000C	2.7889371	35.304383	3.4485296	0.001982	0.1624754	-1.276723
GGT8P	2.7828671	17.81535	2.3937576	0.0243914	0.3785329	-3.417845
KIAA2022	2.7819664	8.9727167	3.1240888	0.0044287	0.2176909	-1.970313
LHFPL5	2.7752895	16.7559	4.8188095	5.77E-05	0.064665	1.7885204
MALAT1	2.7627972	25.107133	3.1642428	0.0040142	0.2107	-1.885784
MPO	2.7595664	27.412583	2.3947898	0.0243358	0.3782092	-3.415945
C1orf88	2.7593706	15.51935	4.6148132	9.82E-05	0.0671784	1.3285847
CXCL6	2.7554839	13.794608	4.0658636	0.0004097	0.1093626	0.0905827
ZNF507	2.7536336	13.541733	4.6587189	8.76E-05	0.064665	1.4276724
lincRNA:ch	2.7525566	15.85675	4.3101926	0.0002173	0.0888702	0.6407871
IGFL3	2.7430993	8.7969	2.2168841	0.0358262	0.4210607	-3.73581
VNN3	2.7423748	19.865417	2.6627617	0.0132763	0.3051802	-2.907282
lincRNA:ch	2.7408084	18.95075	5.4715016	1.06E-05	0.0416449	3.2443105
PSMD9	2.7391767	19.362739	4.2248683	0.0002713	0.0920431	0.4483659
GREM2	2.7283357	17.209267	2.6490414	0.0137033	0.3072994	-2.934038
AZU1	2.7282476	23.335492	2.4571516	0.0211858	0.359043	-3.300304
IL1R2	2.7129161	29.697442	3.2250055	0.003457	0.2045187	-1.757113
SFRS5	2.7090685	38.796333	3.6823231	0.0010972	0.1399536	-0.76449
HIP1	2.7080308	20.59085	2.6601808	0.0133557	0.305256	-2.912321
lincRNA:ch	2.7058182	30.005033	4.0929902	0.0003819	0.1067713	0.1515272
lincRNA:ch	2.6985734	24.2186	4.3754872	0.0001833	0.0811076	0.7881623
ENST0000C	2.6979273	11.06945	2.2186792	0.0356891	0.4210607	-3.73266
ENST0000C	2.696635	11.81635	2.316771	0.0288808	0.3980667	-3.558102
lincRNA:ch	2.6876671	23.758583	2.4708886	0.0205445	0.3552327	-3.274598
lincRNA:ch	2.6755664	7.0082833	2.4299086	0.0225126	0.3671454	-3.351037
A_33_P338'	2.6743916	23.694883	2.9006142	0.0075958	0.2548004	-2.432655
ENST0000C	2.671572	13.116483	2.649478	0.0136895	0.3072994	-2.933188
lincRNA:ch	2.669614	13.31295	3.6613255	0.0011575	0.1429549	-0.810833
BEGAIN	2.6639944	23.959833	3.1887126	0.0037801	0.2066715	-1.834075
LOC100133	2.6591776	17.346933	6.5822871	6.34E-07	0.0110724	5.6156471
ENST0000C	2.654635	23.3688	2.8809298	0.0079602	0.2570639	-2.472664
ENST0000C	2.653421	10.132033	2.2358612	0.0344004	0.4170539	-3.702431
lincRNA:ch	2.6503664	11.236633	3.8978212	0.000632	0.1203925	-0.285786
CHST11	2.6482713	31.337717	5.0219406	3.40E-05	0.0638678	2.244796
LOC10013C	2.6477231	11.894983	3.4332095	0.0020597	0.1644633	-1.30997
A_23_P147(	2.6476643	7.2403333	2.3022754	0.0298062	0.399347	-3.584196
TCTEX1D1	2.6469399	6.99125	4.5122885	0.0001284	0.0677403	1.0970916
A_33_P330(	2.6459413	22.5722	3.3819821	0.0023414	0.1705453	-1.420816
lincRNA:ch	2.6319413	27.173417	3.1715941	0.0039424	0.2097171	-1.870265

NLGN3	2.6312853	10.687425	2.3854584	0.0248426	0.3810375	-3.433098
FGFBP3	2.6285538	13.1054	2.6697081	0.0130649	0.3034471	-2.893709
ENST00000	2.6275552	8.45635	3.0070803	0.0058839	0.2334111	-2.214184
lincRNA:ch	2.6218769	13.093383	3.0457472	0.0053586	0.2266983	-2.134011
lincRNA:ch	2.619449	15.87985	3.518979	0.00166	0.1530728	-1.123299
ORM2	2.6189497	26.334525	2.3196086	0.0287028	0.3972809	-3.552982
CRISP2	2.6175888	16.777133	2.6795432	0.012771	0.3021898	-2.87446
IL17D	2.6070154	7.4881333	2.4110169	0.0234771	0.3724479	-3.386023
SLC25A29	2.6068783	31.19235	2.7600914	0.0105871	0.282881	-2.715462
LOC100131	2.6042937	11.07855	2.9757648	0.0063449	0.2381763	-2.278798
RAPGEF6	2.5987394	17.170378	4.3578936	0.0001919	0.0838482	0.7484443
MBOAT7	2.5969315	35.076417	4.7727918	6.50E-05	0.064665	1.6848841
CYorf15A	2.5961091	18.519317	3.1069785	0.0046175	0.2220927	-2.006205
C8orf60	2.5785455	27.702967	3.4735584	0.0018612	0.1603466	-1.222316
LOC100129	2.5776056	16.116567	3.3900353	0.0022948	0.1688885	-1.403424
GJB6	2.576186	11.570125	2.829449	0.0089934	0.2659148	-2.576706
LOC100129	2.5749427	9.7848333	4.0598908	0.000416	0.1094088	0.0771701
LOC401588	2.5747077	22.965833	3.19921	0.0036838	0.2061571	-1.811846
lincRNA:ch	2.5687161	9.6903333	2.7017164	0.012131	0.2962939	-2.830929
JMJD6	2.5687161	27.337333	3.1766907	0.0038934	0.2089565	-1.859498
lincRNA:ch	2.5686965	8.00065	2.5032137	0.0191055	0.3456829	-3.213786
CLEC5A	2.5677762	12.605133	2.4568004	0.0212024	0.359043	-3.30096
H2AFB3	2.5617846	13.346433	2.7293404	0.0113757	0.292762	-2.776442
CR736977	2.5616671	13.593533	2.5511676	0.0171421	0.3328324	-3.122752
SLC11A1	2.5599392	27.732979	3.4434464	0.0020075	0.1624754	-1.287759
PLAUR	2.559758	32.528008	2.2255628	0.0351677	0.419515	-3.720568
OLT-2	2.5581818	13.7067	2.5688467	0.0164668	0.3274087	-3.088949
lincRNA:ch	2.5579273	21.258417	3.6913304	0.0010723	0.1399536	-0.744592
UBE2J1	2.5552448	32.557	4.5898261	0.0001049	0.0671784	1.2721772
AY358103	2.5550294	17.529283	3.3758648	0.0023775	0.1706151	-1.434018
LOC645158	2.5480196	20.04905	4.655716	8.83E-05	0.064665	1.4208966
DUSP1	2.5442973	40.550008	2.7810654	0.010079	0.2773488	-2.673677
CD69	2.5347636	29.8193	2.5503182	0.0171752	0.3329917	-3.124373
UNQ565	2.5259329	8.08185	2.1370079	0.0424304	0.4431046	-3.874264
SRF	2.5222811	21.755708	2.9625189	0.0065501	0.2412091	-2.306041
lincRNA:ch	2.5187469	14.675267	3.6361264	0.001234	0.1452138	-0.866369
ENST00000	2.5184336	8.0355333	2.4045219	0.0238174	0.3747166	-3.398014
lincRNA:ch	2.5156531	9.9505	2.3784042	0.0252321	0.3829079	-3.446037
TNFRSF12.	2.5151343	26.269892	2.934648	0.0070027	0.246711	-2.363192
GOLGA8A	2.5107091	19.670058	3.5187761	0.0016609	0.1530728	-1.123743
ZNF783	2.5078797	20.050217	3.0613394	0.0051598	0.2240985	-2.101563
lincRNA:ch	2.5011049	16.125783	4.6648689	8.62E-05	0.064665	1.4415487
PCDHB4	2.5009483	11.965917	3.3731311	0.0023937	0.1706805	-1.439916

lincRNA:ch	2.4985203	17.045583	4.1056704	0.0003695	0.1057281	0.1800303
AK124781	2.4962881	20.945283	3.3734629	0.0023918	0.1706805	-1.4392
OR10G4	2.4953091	20.029917	4.2602021	0.0002475	0.0890075	0.5280224
MEGF6	2.4915301	24.911833	3.2569917	0.0031945	0.1976008	-1.689027
FAM7A1	2.4864392	14.601767	3.7215548	0.0009928	0.1387497	-0.677747
lincRNA:ch	2.4859692	18.826967	3.6330877	0.0012436	0.1455183	-0.87306
ENST00000	2.4701287	15.32755	3.5231898	0.0016425	0.1530728	-1.114102
CLEC4C	2.4676909	16.732975	2.631892	0.014255	0.3119592	-2.967378
CDC42EP3	2.4675441	18.63855	3.8822557	0.0006578	0.1222826	-0.32053
ZNF487	2.4650378	10.639883	2.2284009	0.0349547	0.4185103	-3.715575
ENST00000	2.4606322	12.389533	2.5035797	0.0190898	0.3456829	-3.213094
ENST00000	2.4602993	10.721317	3.0297582	0.0055701	0.2292946	-2.167214
INHBA	2.456861	18.254763	2.2242219	0.0352687	0.419515	-3.722925
MYRIP	2.4535832	9.3851333	2.6786113	0.0127986	0.3026374	-2.876285
FAM193B	2.4516056	24.795517	2.843041	0.0087089	0.2636111	-2.549321
lincRNA:ch	2.450842	15.390667	4.2762549	0.0002374	0.0890075	0.5642252
lincRNA:ch	2.4501762	8.9070333	3.1850596	0.0038142	0.2066715	-1.841804
ENST00000	2.4461427	7.2454667	2.3762577	0.0253517	0.3835944	-3.44997
KRTAP1-1	2.439035	7.8744167	2.2447501	0.0337504	0.4147478	-3.686733
ENST00000	2.4363916	11.920767	3.965634	0.0005307	0.1164353	-0.134171
MGC12488	2.4327301	15.074383	2.5054124	0.0190111	0.345414	-3.209633
MNX1	2.4260825	7.6974917	2.6049033	0.0151655	0.3175738	-3.019615
ENST00000	2.4259748	15.564033	2.793622	0.009786	0.2751492	-2.648588
XRRA1	2.4216867	11.488983	2.600823	0.0153078	0.318259	-3.027487
lincRNA:ch	2.4178294	18.759767	3.1850927	0.0038139	0.2066715	-1.841734
TGM3	2.4162042	18.51605	2.7001787	0.0121744	0.2962939	-2.833953
MAML2	2.4150587	17.801175	3.4620909	0.0019156	0.1617143	-1.247258
ENST00000	2.4146378	25.172583	3.8197421	0.0007723	0.1297651	-0.459825
A_33_P327	2.411172	22.232233	3.8999046	0.0006286	0.1203925	-0.281134
PIK3R3	2.4106825	9.27255	3.3622975	0.0024593	0.1731262	-1.463273
lincRNA:ch	2.4052392	9.4897833	4.1695859	0.0003131	0.0994852	0.3238333
SEMG1	2.402958	13.799275	3.6504407	0.00119	0.1439026	-0.834833
B3GNT5	2.4022042	29.694467	3.6146406	0.0013032	0.1469235	-0.913649
RAB26	2.401049	12.26155	2.3571198	0.0264415	0.3876276	-3.48494
STK32B	2.4007357	9.0434167	2.3041496	0.029685	0.399347	-3.580828
LOC100128	2.4006965	17.20565	4.7852043	6.30E-05	0.064665	1.7128463
A_32_P185	2.3991301	10.959783	3.0301538	0.0055648	0.2292946	-2.166394
lincRNA:ch	2.3951161	10.2095	2.1100316	0.044896	0.4504937	-3.920261
lincRNA:ch	2.3950573	16.96205	2.6257125	0.0144589	0.3139876	-2.979364
HCFC1	2.3899273	16.176417	2.1787303	0.0388555	0.4312358	-3.80236
PSIMCT-1	2.388635	11.909917	3.2835308	0.0029914	0.1921322	-1.632359
ENST00000	2.3838182	24.278217	3.1986435	0.0036889	0.2061571	-1.813047
NR1D1	2.3837594	20.660967	3.7440206	0.0009375	0.1353853	-0.627986

A_24_P196(	2.3794909	19.8086	3.7949188	0.000823	0.1315436	-0.515024
lincRNA:ch	2.3767888	10.6211	2.4586794	0.0211136	0.359043	-3.297449
RP11-35N6	2.3767301	7.06685	3.6149697	0.0013021	0.1469235	-0.912926
SMC1B	2.3736559	6.6721667	2.756555	0.0106751	0.2834736	-2.722492
lincRNA:ch	2.3727161	18.331367	2.7180246	0.0116796	0.2935077	-2.798796
C20orf117	2.3704252	13.090817	4.1936826	0.0002941	0.0988466	0.3780993
BX457161	2.3684867	14.209767	2.2042275	0.0368066	0.4228649	-3.757969
ACPP	2.3669056	21.174738	4.1203405	0.0003558	0.1057281	0.2130176
lincRNA:ch	2.3653538	9.2752333	3.0897443	0.0048156	0.2225409	-2.04228
ENST00000	2.3645902	24.849183	3.9439017	0.0005613	0.1164353	-0.182803
BHLHE40	2.3610168	28.520975	2.7763499	0.0101912	0.2789139	-2.683085
IQCG	2.3609091	13.234317	4.4835931	0.0001383	0.0684648	1.0322856
RNF166	2.3601259	26.701383	2.1732174	0.0393118	0.4320798	-3.811913
ABHD2	2.3590881	26.809767	2.335422	0.0277288	0.3926637	-3.524379
ATF7	2.3546923	7.1722583	2.7898358	0.0098735	0.2760175	-2.656159
lincRNA:ch	2.3539972	16.7181	3.7702062	0.0008768	0.1345828	-0.569908
ENST00000	2.3515497	12.660083	2.5267254	0.0181182	0.3388496	-3.169273
ENST00000	2.3463413	19.803117	2.5065694	0.0189616	0.3454014	-3.207447
ENST00000	2.3448923	20.17015	4.5021303	0.0001318	0.0677403	1.0741505
XM_001716	2.3447161	11.4702	2.718316	0.0116717	0.2935077	-2.79822
DOCK6	2.3380587	10.977867	2.7296653	0.0113671	0.2927559	-2.775799
lincRNA:ch	2.3378825	23.616717	3.9426248	0.0005631	0.1164353	-0.185659
WWC2	2.3375497	12.8345	3.1813063	0.0038495	0.2073002	-1.849741
lincRNA:ch	2.336649	22.413067	4.3317992	0.0002054	0.0875525	0.6895455
ENST00000	2.3340252	14.8631	2.81214	0.0093684	0.2696307	-2.611489
CYP4F12	2.3233538	17.803683	2.7567977	0.010669	0.2834736	-2.72201
5-Mar	2.320221	19.62275	2.7000608	0.0121777	0.2962939	-2.834185
FAM156B	2.3180084	20.737733	2.0913318	0.0466795	0.455826	-3.951915
GLUL	2.3148364	34.847633	4.451758	0.0001503	0.0719559	0.9603887
lincRNA:ch	2.310372	12.187233	3.0207645	0.0056926	0.2306829	-2.185859
A_24_P843(	2.3098042	6.1244167	2.1609519	0.0403443	0.4359062	-3.833112
FAM153B	2.3085902	19.09565	2.9260338	0.0071485	0.2496387	-2.380808
SF3B4	2.3066909	13.292767	3.2237751	0.0034675	0.2045187	-1.759727
lincRNA:ch	2.3054965	12.346483	3.06118	0.0051618	0.2240985	-2.101895
OR1F1	2.3054573	11.918317	2.1312978	0.0429419	0.4451527	-3.884033
C3orf57	2.3053399	8.6038167	2.0653772	0.0492596	0.4631831	-3.995531
TOP2A	2.3012397	12.289527	2.1215495	0.043828	0.4469511	-3.90067
C4orf51	2.2989958	6.5192167	2.4379611	0.0221127	0.36422	-3.336076
MGAM	2.2989762	30.051933	2.2195045	0.0356262	0.4210607	-3.731212
RPS15AP10	2.296979	17.657033	2.0897335	0.0468348	0.4561551	-3.954611
A_33_P341(	2.2937678	21.299367	3.1440248	0.004218	0.2139793	-1.928397
lincRNA:ch	2.292828	21.223767	2.1580499	0.0405921	0.4371817	-3.838115
ENST00000	2.2896364	9.9377833	2.5453578	0.0173696	0.3344367	-3.133832

A_33_P326	2.2895189	15.233283	3.8334575	0.0007456	0.1288463	-0.429298
C2CD3	2.2893818	22.7283	4.0481752	0.0004288	0.1102006	0.0508676
LOC283624	2.2876979	13.495533	2.6027782	0.0152395	0.3175738	-3.023716
PNMA6A	2.2809427	12.391983	2.0915205	0.0466612	0.455826	-3.951596
BC030617	2.2808839	28.202533	3.1453444	0.0042044	0.2139793	-1.925618
ENST0000C	2.2788084	15.6149	3.5183967	0.0016625	0.1530728	-1.124571
FAM9B	2.2782406	10.439683	2.880927	0.0079603	0.2570639	-2.47267
KLF7	2.275421	23.437283	4.1765585	0.0003075	0.0992932	0.339533
ENST0000C	2.2742657	12.105567	2.414258	0.023309	0.3719757	-3.380032
ENST0000C	2.2714657	17.31485	2.7170264	0.0117068	0.2935077	-2.800765
C22orf32	2.2687832	15.999433	2.7626589	0.0105236	0.2822202	-2.710355
A_33_P322	2.2684895	7.2637833	2.4092369	0.02357	0.372736	-3.389311
lincRNA:ch	2.2632811	6.6564167	2.8944423	0.0077083	0.2558378	-2.445213
A_33_P340	2.263242	23.62745	2.4016689	0.0239684	0.3756903	-3.403275
lincRNA:ch	2.261558	23.654283	4.0531347	0.0004234	0.1096022	0.0620011
A_33_P333	2.2601287	10.4706	3.5647502	0.0014788	0.1515078	-1.023168
ENST0000C	2.2534713	13.999067	2.9116128	0.0073991	0.2524505	-2.410246
TAGAP	2.2483315	31.339992	2.5062477	0.0189754	0.3454014	-3.208055
JUNB	2.2424573	29.738392	2.3126291	0.0291425	0.398859	-3.565568
ENST0000C	2.2372	10.628217	2.4557882	0.0212504	0.359043	-3.302851
IL17C	2.236965	13.897217	2.8049314	0.0095289	0.2716226	-2.625945
FAM151B	2.2363189	14.302867	3.5005863	0.0017388	0.1562206	-1.163438
ENST0000C	2.2298182	31.5	2.6406834	0.0139696	0.3091988	-2.950301
lincRNA:ch	2.2264895	14.557433	3.5472112	0.0015458	0.1515078	-1.061578
ENST0000C	2.2252951	23.03595	2.808185	0.0094562	0.2700033	-2.619422
CKAP2L	2.224179	10.4076	2.4456204	0.0217383	0.3614175	-3.321818
lincRNA:ch	2.2217706	7.50295	2.6429328	0.0138975	0.3091681	-2.945927
A_33_P324	2.2191664	14.150267	2.9911855	0.0061137	0.2368074	-2.247016
ENST0000C	2.2123916	17.847433	3.5158673	0.0016731	0.1530728	-1.130094
OR13D1	2.2114322	12.27135	2.1263599	0.0433887	0.4460421	-3.892467
IMPA2	2.2105902	35.2583	4.1617959	0.0003195	0.0995993	0.3062958
lincRNA:ch	2.2099636	9.2967	2.13603	0.0425176	0.4431046	-3.875938
SPRED2	2.2069189	8.4825417	2.3523832	0.0267178	0.3884394	-3.493569
lincRNA:ch	2.205793	11.56015	2.8173525	0.009254	0.2692901	-2.601025
ENST0000C	2.2017203	16.179217	2.4649841	0.0208179	0.3573357	-3.285657
LOC100129	2.2008196	11.144583	2.9109715	0.0074104	0.2524505	-2.411554
lincRNA:ch	2.2004671	10.460683	2.685968	0.0125824	0.3010286	-2.861865
RIPK4	2.1999776	13.862333	3.0687419	0.0050679	0.2240985	-2.086135
ENST0000C	2.1996056	15.743817	2.2796519	0.0313044	0.4049891	-3.624717
NEDD9	2.195386	20.869158	3.0993504	0.0047042	0.2225409	-2.022182
AK172772	2.1946909	15.6457	4.6603568	8.72E-05	0.064665	1.431368
ZNF460	2.1940056	16.143983	2.145648	0.0416667	0.4415694	-3.85945
MARCKS	2.1900993	41.642358	3.5308188	0.0016112	0.1530728	-1.097431

RAB41	2.1850965	8.6804667	3.342353	0.0025847	0.1784419	-1.50621
CNTN2	2.1844993	12.827325	2.539963	0.0175833	0.3352101	-3.144108
EPB41	2.1820028	15.566367	3.1251808	0.0044169	0.2176909	-1.968019
lincRNA:ch	2.1774014	16.920517	2.6463579	0.0137883	0.3079147	-2.939263
LOC100127	2.1759916	12.765317	2.9196049	0.0072592	0.2504528	-2.393939
lincRNA:ch	2.175972	12.128433	2.4259622	0.022711	0.3678645	-3.358359
C6orf204	2.1754629	17.412267	4.2582107	0.0002488	0.0890075	0.5235319
ANKHD1	2.1748951	29.47105	4.0132089	0.0004694	0.1111414	-0.027579
AK097991	2.1703133	17.86435	3.3442999	0.0025722	0.1783616	-1.502023
SIRPB1	2.1701305	28.678106	2.10582	0.0452923	0.4518753	-3.927407
AK125393	2.1682378	20.503117	2.3871397	0.0247506	0.3809379	-3.43001
ENST0000C	2.1671021	25.633883	2.6077085	0.0150684	0.3175738	-3.014199
CPHL1	2.163049	8.3358333	2.7225528	0.0115571	0.2935077	-2.789856
hg18:lincRN	2.1610909	23.8231	3.2485458	0.0032619	0.1995927	-1.707028
PLEKHH1	2.1605231	8.9794833	2.2979499	0.0300875	0.4000235	-3.591963
lincRNA:ch	2.1603469	8.8951333	2.5404981	0.017562	0.3350295	-3.14309
SGSM2	2.1602196	28.005192	3.1974563	0.0036997	0.2061571	-1.815562
lincRNA:ch	2.1597399	6.57615	2.227301	0.0350371	0.4189229	-3.71751
RNF19B	2.1581734	25.446283	2.4330268	0.0223569	0.3659732	-3.345247
ENST0000C	2.1560587	6.3600833	2.173181	0.0393148	0.4320798	-3.811976
LMTK2	2.1551874	24.785775	4.4814926	0.0001391	0.0684648	1.0275416
LOC100293	2.1551385	11.546967	2.2631242	0.0324419	0.4086958	-3.65416
LOC100131	2.1543944	18.4506	3.0114473	0.0058222	0.232381	-2.20515
ENST0000C	2.1537678	20.565533	3.6767398	0.0011129	0.1409288	-0.776819
ENST0000C	2.1521622	18.6375	2.9491967	0.0067629	0.241919	-2.333388
lincRNA:ch	2.1514769	9.9069833	2.4949485	0.0194643	0.3470706	-3.229378
lincRNA:ch	2.150772	17.465583	2.8188443	0.0092215	0.2690606	-2.598028
A_32_P154	2.150165	13.2062	3.310143	0.0028002	0.1871229	-1.575379
AK130290	2.1492643	11.108767	3.0814115	0.0049142	0.2225409	-2.059694
ZBED4	2.1482462	16.832433	3.5775136	0.0014318	0.1491954	-0.995186
C22orf36	2.1481287	15.699133	2.6937705	0.0123568	0.2980388	-2.846549
ENST0000C	2.1470322	21.025667	3.2961384	0.0028993	0.1891663	-1.605384
NCRNA001	2.1390629	27.25135	3.0722328	0.0050251	0.2240985	-2.078854
SIGLEC10	2.1379664	31.245083	3.1341884	0.0043207	0.2154134	-1.949091
LARP4	2.1361259	17.62845	3.9366718	0.0005718	0.116472	-0.198973
lincRNA:ch	2.1359692	15.291383	3.1931318	0.0037393	0.2064525	-1.82472
C3orf62	2.1350098	21.9177	2.356786	0.0264609	0.3877491	-3.485549
FAM157A	2.1348336	17.28335	2.176664	0.039026	0.4318941	-3.805943
lincRNA:ch	2.1334042	21.045267	3.3250592	0.0026983	0.1834701	-1.543375
A_33_P328	2.1334042	17.377267	2.7603418	0.0105809	0.282881	-2.714964
lincRNA:ch	2.131172	6.1373667	2.0847467	0.0473224	0.4586444	-3.963016
BM977558	2.1305259	23.309417	2.8517455	0.0085313	0.2610847	-2.531752
lincRNA:ch	2.1273147	9.16335	2.1116298	0.0447464	0.4501553	-3.917547

ENST0000C	2.1269818	11.345133	3.1093665	0.0045907	0.2216041	-2.001201
ZNF669	2.124358	15.392767	3.3666621	0.0024327	0.1726719	-1.453866
lincRNA:ch	2.1228895	18.706917	2.9321327	0.007045	0.2474522	-2.368338
lincRNA:ch	2.1220476	7.3061333	2.3540273	0.0266216	0.3883799	-3.490575
lincRNA:ch	2.1212056	18.09535	2.6403259	0.0139811	0.3092574	-2.950996
FLJ10213	2.1167608	27.175633	2.4682269	0.0206673	0.3558142	-3.279586
lincRNA:ch	2.1148224	15.910183	2.3752324	0.0254091	0.3835944	-3.451848
RALGAPA:	2.106814	13.4057	2.224388	0.0352562	0.419515	-3.722633
LOC100131	2.1055021	7.2101167	2.1267517	0.0433531	0.445974	-3.891798
OOEP	2.1042294	14.7175	2.2501123	0.0333637	0.4131238	-3.677245
THBS4	2.103965	17.651375	2.7225334	0.0115576	0.2935077	-2.789894
PLEKHA7	2.1035636	13.237467	2.0631756	0.0494841	0.463389	-3.999213
ZNF281	2.101351	29.52005	4.7481504	6.94E-05	0.064665	1.6293569
ENST0000C	2.1012727	18.919717	2.5632218	0.0166789	0.3294068	-3.099718
TPST1	2.0987566	28.330808	2.2750037	0.0316206	0.4057901	-3.633011
NCRNA00(	2.0976308	18.188217	2.7217534	0.0115787	0.2935077	-2.791435
lincRNA:ch	2.096358	13.244	4.1122597	0.0003633	0.1057281	0.1948454
BEX4	2.0959469	22.51305	2.5473537	0.0172911	0.3342535	-3.130027
ENST0000C	2.0952028	7.1874833	2.9255107	0.0071574	0.2496387	-2.381876
FLJ25006	2.0929315	17.244617	3.5523695	0.0015258	0.1515078	-1.050286
BEND7	2.0906406	17.219067	2.8987471	0.0076296	0.2548004	-2.436455
ARRDC5	2.0867049	18.401717	3.0626935	0.0051429	0.2240985	-2.098742
SLC45A4	2.0839245	32.197083	4.0855019	0.0003894	0.107147	0.1346989
CLEC7A	2.0837238	25.285429	3.8099084	0.000792	0.1305667	-0.4817
SPNS2	2.0833175	25.4093	3.781647	0.0008515	0.1328476	-0.544507
LOC100292	2.0827497	6.2424833	2.1704987	0.0395386	0.4331687	-3.816619
RAD23B	2.082717	25.214078	3.3909899	0.0022893	0.1688885	-1.401362
lincRNA:ch	2.0793622	13.180067	2.7514111	0.0108043	0.2849344	-2.73271
VHLL	2.0786182	12.5125	3.6407822	0.0012195	0.1452138	-0.856115
ADPRHL1	2.0782266	13.340833	4.3249635	0.0002091	0.0880502	0.6741185
ZFP36	2.0778643	41.637692	2.4697596	0.0205965	0.3554645	-3.276714
CITED4	2.0751133	33.139983	3.7321908	0.0009662	0.1367135	-0.654196
A_33_P334'	2.0732923	8.5850333	2.1592212	0.0404919	0.4363715	-3.836096
PPP4R1L	2.070972	11.753758	3.8823126	0.0006577	0.1222826	-0.320403
TCP11L2	2.0682601	21.692417	2.7212086	0.0115934	0.2935077	-2.792511
C1orf55	2.0637175	25.230683	4.6034459	0.0001012	0.0671784	1.3029247
ENST0000C	2.0636	8.5237833	2.1326572	0.0428196	0.4448582	-3.881709
lincRNA:ch	2.062993	21.3808	3.1623234	0.0040331	0.2107	-1.889834
lincRNA:ch	2.0621315	15.615133	2.141693	0.0420147	0.4421478	-3.866236
ENST0000C	2.0615245	16.38455	2.616071	0.0147824	0.3165792	-2.998035
CCNY	2.0569818	30.985617	2.9315707	0.0070545	0.2475363	-2.369487
ATG16L2	2.052028	41.491333	3.5698134	0.00146	0.151208	-1.012071
RCN3	2.0517147	18.7152	2.7932469	0.0097946	0.2751707	-2.649339

UPP2	2.049835	11.046	2.5825032	0.0159622	0.3231011	-3.06275
LOC100129	2.0461343	11.97385	2.2661966	0.0322277	0.4080885	-3.648697
USP12	2.0441566	13.846233	3.966727	0.0005292	0.1164353	-0.131724
RRP12	2.0438434	27.7305	3.4054407	0.002208	0.1688885	-1.370119
MYO15B	2.0404559	21.832883	2.7872622	0.0099334	0.2763087	-2.661302
LOC100128	2.0396727	6.89955	2.6820995	0.0126956	0.3012221	-2.86945
ALOX15	2.0366573	19.206717	2.3018824	0.0298316	0.399347	-3.584902
H3F3B	2.0358807	36.555944	4.1080522	0.0003673	0.1057281	0.1853851
lincRNA:ch	2.0322322	10.273083	2.5000315	0.0192429	0.3461253	-3.219792
GPR27	2.0251832	15.279483	2.6137351	0.0148618	0.3167176	-3.002553
lincRNA:ch	2.0240867	23.324817	2.9213275	0.0072294	0.2503341	-2.390422
HIGD2B	2.0224615	14.2247	2.673733	0.0129439	0.3029433	-2.885836
lincRNA:ch	2.021835	15.404433	2.9936753	0.0060772	0.2365153	-2.241878
FLJ33544	2.0207972	8.0900167	2.2938989	0.0303531	0.401063	-3.599228
lincRNA:ch	2.0198573	7.7624167	2.9100414	0.0074269	0.2524505	-2.41345
PLIN4	2.0197986	28.016567	2.1301433	0.043046	0.4452977	-3.886006
AQP7P3	2.0160783	11.930333	2.9470704	0.0067974	0.2426586	-2.337748
TMEM185I	2.0154987	22.141187	3.4225686	0.0021153	0.1668826	-1.333036
STX11	2.0144336	31.038933	2.5338294	0.0178293	0.3355597	-3.155777
ENST0000C	2.0139245	20.441167	2.9559023	0.006655	0.2412766	-2.31963
ENST0000C	2.0137874	11.827783	2.3643261	0.0260262	0.3868951	-3.471792
LOC401052	2.0128867	7.71995	2.6046816	0.0151732	0.3175738	-3.020043
ENST0000C	2.0121427	7.4751833	3.5333961	0.0016007	0.1530728	-1.091797
HTR3B	2.0110168	8.3603917	2.3318211	0.0279479	0.3936325	-3.530903
NRBP2	2.0109678	13.754183	2.6239291	0.0145182	0.3143724	-2.982821
BCL2A1	2.0097016	39.391061	2.5024422	0.0191388	0.3456829	-3.215242
lincRNA:ch	2.0092252	24.005567	2.2656828	0.0322634	0.4082453	-3.649611
FEM1B	2.0079916	22.726317	3.8136391	0.0007845	0.1300441	-0.473402
MED29	2.0060923	15.935033	2.2740766	0.031684	0.4057901	-3.634664
lincRNA:ch	2.0014028	13.895875	2.0910916	0.0467028	0.4559262	-3.95232
ZNF267	2.0003748	29.5323	3.7118055	0.0010178	0.1387497	-0.699322
CXCR4	1.9990982	45.662027	4.4227965	0.0001621	0.0745339	0.8949849
SF1	1.9959448	29.646546	2.8579608	0.0084065	0.2602147	-2.519191
CBX4	1.9953916	23.957092	2.8316053	0.0089477	0.2659145	-2.572365
lincRNA:ch	1.993835	20.028867	3.102701	0.0046659	0.2221665	-2.015166
OR2J2	1.9936783	13.2286	3.04344	0.0053887	0.2266983	-2.138807
ENST0000C	1.9927385	15.2334	2.6902479	0.0124582	0.299039	-2.853467
lincRNA:ch	1.9923273	10.22525	2.7817334	0.0100632	0.2773488	-2.672344
EXOSC6	1.9919357	21.609583	4.5797624	0.0001076	0.0671784	1.2494563
ABCD3	1.989351	18.015783	3.2213282	0.0034885	0.2049084	-1.764925
lincRNA:ch	1.9872168	18.1867	3.9832569	0.0005071	0.1164353	-0.094707
C20orf107	1.9858853	19.608633	3.6235764	0.001274	0.1468158	-0.893994
ENST0000C	1.9856503	10.795633	2.4494076	0.0215554	0.3614175	-3.314759

A_33_P328.	1.9813231	23.490017	2.1881016	0.0380908	0.4276377	-3.786084
SRRM2	1.9798872	31.622306	3.4790736	0.0018356	0.1598952	-1.210312
AWAT2	1.9756448	9.24665	2.3399315	0.0274566	0.391968	-3.516201
lincRNA:ch	1.9728937	14.960342	2.4851582	0.0198974	0.3495379	-3.247809
IVNS1ABP	1.9718853	23.81505	3.2503449	0.0032474	0.1994989	-1.703195
DDX5	1.9716797	41.851775	3.6205453	0.0012838	0.1469235	-0.900662
HERV-FRI	1.9697315	7.8272833	3.0626632	0.0051432	0.2240985	-2.098805
lincRNA:ch	1.9688112	25.598767	3.7853816	0.0008434	0.1321738	-0.536213
lincRNA:ch	1.9683021	14.8582	3.5305899	0.0016121	0.1530728	-1.097931
LOC284751	1.9677343	23.990983	2.4431341	0.0218592	0.362517	-3.326449
LOC644075	1.9665594	15.799583	3.4958595	0.0017596	0.1572829	-1.173744
ENST0000C	1.9651497	11.733983	2.5377866	0.0176702	0.3355597	-3.148251
VAMP1	1.9629743	12.537245	2.6760198	0.0128756	0.3029433	-2.88136
lincRNA:ch	1.9614685	10.238317	3.1227532	0.0044432	0.2177896	-1.973117
SORD	1.9605091	21.176633	3.3945702	0.0022689	0.1688885	-1.393625
IL20RB	1.9594909	17.9403	3.9565695	0.0005433	0.1164353	-0.15446
AK123255	1.9592755	7.1657833	2.4018097	0.0239609	0.3756903	-3.403016
lincRNA:ch	1.9591189	11.915517	2.4271848	0.0226494	0.3678645	-3.356091
LHX3	1.959034	15.549956	2.5467684	0.0173141	0.3342535	-3.131143
PTPRJ	1.9586979	21.898625	4.2941319	0.0002266	0.0888702	0.6045512
ENST0000C	1.9573175	17.39745	2.9724762	0.0063953	0.2393071	-2.285567
SSH3	1.9566517	19.652617	2.67429	0.0129272	0.3029433	-2.884746
SLC2A3	1.95658	27.028711	3.4012503	0.0022313	0.1688885	-1.379183
ENST0000C	1.9521483	10.21265	2.7113396	0.0118627	0.295381	-2.811979
lincRNA:ch	1.9519133	20.40885	2.099172	0.0459242	0.4546752	-3.938667
ACVR1B	1.9516294	9.9476417	2.865425	0.0082589	0.2587405	-2.50409
CSF2RA	1.9505818	28.934383	3.1116793	0.0045649	0.2209678	-1.996352
OSTalpha	1.9501706	12.015033	2.3122205	0.0291685	0.398859	-3.566305
A_33_P328	1.9499944	7.0390833	2.3240927	0.0284235	0.3970507	-3.544884
LOC100131	1.9468811	23.923433	2.4401927	0.022003	0.3632416	-3.331924
lincRNA:ch	1.9430042	21.815733	3.2821477	0.0030016	0.1921322	-1.635316
CAMP	1.9426713	37.389917	2.1345274	0.0426519	0.4437753	-3.87851
LOC100131	1.9419077	10.235867	2.7807539	0.0100864	0.2773488	-2.674299
BAALC	1.940772	10.386833	2.7409923	0.0110706	0.2880918	-2.753376
TBX20	1.9401455	6.0967667	2.4305097	0.0224825	0.366855	-3.349921
A_33_P323	1.9382657	7.0879667	2.8347827	0.0088808	0.2648239	-2.565967
LOC100132	1.9358182	16.27115	2.9621202	0.0065564	0.2412091	-2.306861
LOC100132	1.9353874	10.360117	2.4111002	0.0234728	0.3724479	-3.385869
ENST0000C	1.9314713	9.39225	2.08089	0.0477026	0.459347	-3.969506
tcag7.1227	1.9265958	16.4339	3.7925385	0.0008281	0.1315436	-0.520313
HSD17B13	1.9264587	10.262117	2.1915039	0.0378166	0.4266764	-3.780163
lincRNA:ch	1.9248727	7.1565667	3.6890173	0.0010787	0.1399536	-0.749703
C1QTNF6	1.9243832	18.220883	2.1963176	0.0374316	0.4250149	-3.771776

KIF7	1.9220531	6.2493667	2.3119808	0.0291837	0.398859	-3.566736
RBBP6	1.9210056	20.765908	3.9741048	0.0005192	0.1164353	-0.115205
GSG2	1.918	8.4673167	4.0194772	0.0004618	0.1111414	-0.013523
lincRNA:ch	1.9173538	14.162167	3.6617024	0.0011564	0.1429549	-0.810002
PROC	1.9153697	16.301056	2.6470177	0.0137673	0.3078285	-2.937978
A_33_P339	1.9119888	9.4005333	2.3781604	0.0252457	0.3829473	-3.446484
lincRNA:ch	1.9115385	23.729417	2.1200512	0.0439656	0.4470926	-3.903223
lincRNA:ch	1.9093846	27.91705	2.0787374	0.047916	0.4593556	-3.973126
ENST0000C	1.9075441	11.276417	2.3032639	0.0297422	0.399347	-3.58242
ERLIN1	1.9071524	18.01835	3.1956461	0.0037162	0.2061571	-1.819396
FLJ36031	1.9059972	33.597433	2.7645144	0.010478	0.2820898	-2.706663
RNF222	1.9050867	19.751958	2.5876427	0.015776	0.3214	-3.052871
CRIPAK	1.904842	24.696117	2.9838892	0.0062221	0.237308	-2.262062
CAMK1D	1.9042203	29.126271	3.4076631	0.0021958	0.1688885	-1.36531
ENST0000C	1.9028252	16.502733	3.5188759	0.0016605	0.1530728	-1.123525
ENST0000C	1.9010042	15.322183	3.1907432	0.0037613	0.2065576	-1.829777
ENST0000C	1.9000839	19.128667	3.3965455	0.0022577	0.1688885	-1.389355
lincRNA:ch	1.8963832	12.258517	2.5102535	0.0188048	0.3440884	-3.200482
LOC100132	1.8960112	14.076533	2.2171669	0.0358046	0.4210607	-3.735314
ENST0000C	1.8952671	17.572567	3.2249264	0.0034577	0.2045187	-1.757281
AK096917	1.8950517	14.78365	3.072022	0.0050277	0.2240985	-2.079293
CAMKK1	1.8949783	18.999838	2.9879785	0.0061611	0.2370945	-2.253631
LCE6A	1.8932699	16.800467	3.0880349	0.0048356	0.2225409	-2.045854
A_33_P324	1.8918797	13.57055	2.8115966	0.0093805	0.2696307	-2.612579
KCNC4	1.8912336	13.5016	4.7445697	7.00E-05	0.064665	1.6212862
lincRNA:ch	1.8901175	19.27065	2.1603867	0.0403925	0.4359062	-3.834086
ENST0000C	1.8892364	10.9445	2.2546345	0.0330407	0.4111299	-3.669231
lincRNA:ch	1.8883357	13.587467	2.0701114	0.0487797	0.4612558	-3.987603
CA5A	1.8858294	17.9284	2.9175109	0.0072956	0.2505573	-2.398214
LOC100133	1.8849678	7.2935333	2.6334321	0.0142046	0.3110514	-2.964389
KRT13	1.8845371	13.4295	2.7577618	0.010645	0.2833447	-2.720094
OR4P4	1.8838517	6.5595833	3.0472686	0.0053389	0.2264439	-2.130848
lincRNA:ch	1.8837343	23.657083	2.2287094	0.0349316	0.4185103	-3.715032
LOC339929	1.8829902	17.955117	3.0634934	0.0051329	0.2240985	-2.097075
lincRNA:ch	1.8825203	20.197917	2.890078	0.0077888	0.2563428	-2.454086
ANP32C	1.880014	20.53205	3.4729771	0.0018639	0.1603466	-1.223581
CD101	1.8769594	16.77165	2.0821827	0.0475749	0.4592922	-3.967332
ENST0000C	1.8760979	7.4933833	3.2757179	0.0030498	0.1927588	-1.649058
LOC10027C	1.8758825	16.943267	2.6041966	0.0151901	0.3175738	-3.020979
lincRNA:ch	1.8750406	26.060883	2.4173819	0.023148	0.3704211	-3.374254
lincRNA:ch	1.8745315	16.177117	2.3356562	0.0277146	0.3926637	-3.523955
LOC10013C	1.8737874	16.38315	2.2496066	0.0334	0.4131238	-3.67814
ITGB4	1.8734741	21.467017	2.3541186	0.0266162	0.3883799	-3.490409

LOC440900	1.8728867	12.689717	2.6846017	0.0126223	0.3010286	-2.864545
ENPP3	1.8715357	11.469967	2.1929283	0.0377023	0.4265606	-3.777683
CTSK	1.8711343	26.195458	4.1738507	0.0003097	0.0992932	0.3334359
FLJ38717	1.8647217	16.424567	2.3052938	0.0296113	0.399347	-3.578771
FAM101B	1.8631357	38.051417	2.785164	0.0099825	0.276697	-2.665494
LOC284009	1.8604336	13.161517	3.4782912	0.0018392	0.1598952	-1.212015
LNP1	1.8599441	18.253433	2.610434	0.0149746	0.3172273	-3.008934
ENST00000	1.8592	19.405867	3.6057231	0.001333	0.1482829	-0.933253
DAPK2	1.8561161	28.760142	2.1292651	0.0431253	0.4454091	-3.887506
lincRNA:ch	1.8546965	6.0347	3.1612079	0.0040442	0.2107	-1.892187
MYO7B	1.852758	18.20805	2.770528	0.0103313	0.2805518	-2.694689
NBPF22P	1.8527385	14.393167	2.0614079	0.0496651	0.4634338	-4.002168
PBX2	1.851965	26.288675	2.9616641	0.0065636	0.2412091	-2.307798
lincRNA:ch	1.8493021	29.346742	2.2726896	0.0317791	0.4057901	-3.637136
lincRNA:ch	1.8492531	21.584733	3.0587508	0.0051923	0.2245865	-2.106955
lincRNA:ch	1.8466294	27.729567	2.372243	0.0255769	0.3843614	-3.45732
LOC284441	1.8450825	28.921783	2.9376458	0.0069526	0.2456899	-2.357056
ANKRD45	1.8442014	34.654433	2.4927733	0.0195598	0.3479565	-3.233476
PIM3	1.8417734	41.8033	2.4479394	0.0216262	0.3614175	-3.317496
ENST00000	1.8388755	17.603367	2.1722017	0.0393964	0.4325574	-3.813672
HIGD1C	1.8384252	8.68385	3.0554881	0.0052336	0.2245871	-2.113748
DIP2A	1.8380336	19.480183	2.4731688	0.0204398	0.3541692	-3.270324
ENST00000	1.8378769	17.658317	2.3213523	0.0285939	0.3971357	-3.549834
PDE4B	1.8366042	34.2083	2.2864719	0.0308457	0.4033021	-3.612528
MARCKSL	1.8352727	25.814833	3.0239552	0.0056489	0.2304636	-2.179247
CDR2	1.8352629	21.975392	2.1718828	0.039423	0.4325883	-3.814224
JUND	1.834235	25.527017	2.4277934	0.0226187	0.3676756	-3.354962
XRN2	1.8335888	27.311667	3.0542487	0.0052493	0.2245871	-2.116327
NBPF14	1.8307105	30.351417	2.3176043	0.0288285	0.3979668	-3.556599
CAMKK2	1.8279594	28.159308	2.6341725	0.0141805	0.3109118	-2.962951
LOC100292	1.8264028	23.452683	2.3725872	0.0255575	0.3843614	-3.45669
MTUS2	1.8261776	7.350525	2.74743	0.0109054	0.2861334	-2.740611
ENST00000	1.8236224	12.92445	2.4616818	0.0209723	0.3574747	-3.291836
lincRNA:ch	1.8234462	22.3853	2.9774306	0.0063195	0.2377409	-2.275368
LOC644422	1.8209594	15.712317	3.2118263	0.0035712	0.2061571	-1.785097
lincRNA:ch	1.8208615	22.3643	2.6756735	0.0128859	0.3029433	-2.882038
lincRNA:ch	1.8208028	10.60885	2.0732675	0.0484621	0.4605658	-3.982311
HHIP	1.8205678	11.07645	2.1957666	0.0374755	0.425099	-3.772737
RBM33	1.8199869	15.675644	2.258648	0.0327564	0.4101764	-3.662111
ZBTB25	1.8190014	19.940783	3.0792976	0.0049395	0.2225409	-2.064108
C21orf66	1.8186098	14.966117	2.4885204	0.0197476	0.3487739	-3.241484
ENST00000	1.8179245	6.7298	3.1210828	0.0044613	0.2180682	-1.976624
PIGW	1.8172783	16.34745	2.8224572	0.0091432	0.2684883	-2.590768

MPPE1	1.8172	30.648917	3.4086763	0.0021902	0.1688885	-1.363118
A_33_P329(	1.8168867	23.635383	2.1200192	0.0439686	0.4470926	-3.903277
AF461897	1.8162993	6.7716833	2.0983913	0.0459989	0.4548469	-3.939987
lincRNA:ch	1.8144	19.9948	3.2943758	0.002912	0.1891663	-1.609157
TLE3	1.8121678	28.4081	2.7355856	0.0112112	0.2902381	-2.764085
LOC10013(	1.8104448	19.304367	2.8338823	0.0088997	0.265162	-2.56778
ACVR2A	1.8102881	14.6769	2.2184162	0.0357092	0.4210607	-3.733122
CPA3	1.8101902	20.708683	2.7538593	0.0107426	0.2841935	-2.727848
SNAP23	1.8101217	25.850592	2.9525726	0.0067083	0.2412766	-2.326464
lincRNA:ch	1.8093091	19.073133	3.3801613	0.0023521	0.1705453	-1.424746
LOC100128	1.8065678	12.425467	2.2027863	0.0369198	0.4230074	-3.760488
LOC401913	1.8060392	7.4320167	2.3414854	0.0273634	0.3917302	-3.513381
AB030181	1.8057063	13.6626	2.1745972	0.0391971	0.4318941	-3.809524
lincRNA:ch	1.8044727	12.96575	3.4088177	0.0021895	0.1688885	-1.362812
LOC645676	1.8025147	22.518417	4.2902752	0.0002289	0.0888702	0.5958506
C6orf58	1.8021622	12.433517	2.3070645	0.0294975	0.399347	-3.575586
ITCH	1.8015063	25.549125	2.8576601	0.0084125	0.2602147	-2.5198
ERF	1.8007524	21.744917	2.6761061	0.012873	0.3029433	-2.881191
lincRNA:ch	1.8003217	7.7544833	2.2468569	0.033598	0.4140394	-3.683007
lincRNA:ch	1.8001455	29.819533	2.5601455	0.016796	0.3294068	-3.105602
LOC100288	1.799421	13.72665	3.3166731	0.0027552	0.1859295	-1.561374
LOC100128	1.797649	9.9173083	2.6850092	0.0126104	0.3010286	-2.863746
CCNG2	1.7973024	21.040833	3.3461148	0.0025606	0.1781246	-1.498118
C14orf132	1.7965427	15.0836	2.4961092	0.0194136	0.3468525	-3.22719
ENST0000(	1.7957594	6.5174667	2.3399594	0.027455	0.391968	-3.51615
FLJ26332	1.7950545	15.870867	3.0616942	0.0051553	0.2240985	-2.100824
LENEP	1.7934881	11.1902	2.124754	0.0435349	0.4462653	-3.895207
PFAS	1.793449	15.911233	2.154004	0.0409399	0.4392951	-3.845084
lincRNA:ch	1.7921371	15.16165	3.156245	0.0040936	0.2115763	-1.902653
CR987211	1.7914322	32.14225	3.3069588	0.0028225	0.1878045	-1.582205
OGFRL1	1.791158	34.523883	3.7097296	0.0010232	0.1387497	-0.703914
ENST0000(	1.7885538	15.7556	2.3199579	0.028681	0.3971357	-3.552352
MAP6D1	1.7875748	25.418633	3.5498261	0.0015357	0.1515078	-1.055854
lincRNA:ch	1.7858322	27.664817	2.5420797	0.0174991	0.3347443	-3.140078
ENST0000(	1.7856951	15.226633	2.4155251	0.0232436	0.371378	-3.377689
lincRNA:ch	1.7854993	9.3758	2.211436	0.0362453	0.4217107	-3.745359
ENST0000(	1.7854406	11.14155	2.9522651	0.0067133	0.2412766	-2.327095
lincRNA:ch	1.785235	21.255675	2.5343429	0.0178086	0.3355597	-3.154801
A_33_P339:	1.7849902	6.6124333	2.3260051	0.0283051	0.3963285	-3.541427
lincRNA:ch	1.7840699	27.426117	2.7485241	0.0108775	0.2858509	-2.73844
EDEM1	1.782758	25.226133	3.1243933	0.0044254	0.2176909	-1.969673
HMGN5	1.782059	17.729238	3.8356911	0.0007413	0.1288463	-0.424325
C6orf26	1.7813874	18.9651	2.495266	0.0194504	0.3469998	-3.228779

SLC9A8	1.7811329	27.886017	2.678086	0.0128142	0.3028008	-2.877314
NIPAL2	1.7809762	10.72015	2.2751877	0.031608	0.4057901	-3.632683
PPP4R1	1.7801734	25.233133	2.2776793	0.0314383	0.4049891	-3.628238
lincRNA:ch	1.7798993	22.759567	3.3812158	0.0023459	0.1705453	-1.42247
SOX4	1.7796154	18.431758	2.8161829	0.0092795	0.2693612	-2.603373
C20orf106	1.7780196	26.500367	2.8163768	0.0092753	0.2693612	-2.602984
CLSTN2	1.7770406	8.2642	2.1882424	0.0380794	0.4276377	-3.785839
lincRNA:ch	1.7761203	6.4202833	2.1189498	0.044067	0.4472151	-3.905098
C14orf138	1.7741427	26.137067	2.754553	0.0107252	0.2841056	-2.72647
MAGOHB	1.7723315	19.809358	4.7446957	7.00E-05	0.064665	1.6215703
ZDHHC18	1.7712448	31.085133	2.6560178	0.0134846	0.3058213	-2.920442
AGBL1	1.7700406	14.869808	3.8285679	0.000755	0.1288463	-0.440183
LOC202781	1.7692671	19.184317	3.2088664	0.0035973	0.2061571	-1.791376
ZNF628	1.7655664	26.025767	3.1840224	0.0038239	0.2068781	-1.843997
OCLN	1.765449	7.6043333	2.8124128	0.0093624	0.2696307	-2.610941
SFRS18	1.7644327	24.192782	3.3724347	0.0023979	0.1706805	-1.441418
CCKBR	1.7629133	7.493675	3.4727534	0.001865	0.1603466	-1.224068
RPL13AP3	1.7627664	42.20825	2.4379265	0.0221144	0.36422	-3.33614
lincRNA:ch	1.7620224	6.6470833	2.8183323	0.0092326	0.2691008	-2.599057
MAFB	1.7611168	39.063529	2.3865627	0.0247821	0.3810375	-3.43107
lincRNA:ch	1.7602797	6.7064667	2.437196	0.0221504	0.3646413	-3.337499
ENST0000C	1.7596727	18.079483	2.9010696	0.0075875	0.2548004	-2.431728
LOC729603	1.7591636	21.308117	2.3523592	0.0267192	0.3884394	-3.493612
CEP68	1.7581846	18.54895	3.3596865	0.0024754	0.1737197	-1.468899
TTLL13	1.7581063	13.502417	2.3112799	0.0292282	0.398859	-3.567999
lincRNA:ch	1.7560503	13.997667	2.7791134	0.0101253	0.2779816	-2.677573
AMPD2	1.7552867	34.230817	2.3317259	0.0279537	0.3936325	-3.531076
lincRNA:ch	1.7545035	16.617883	2.9292057	0.0070945	0.2484419	-2.374324
ANKRD6	1.752565	16.196833	2.7899274	0.0098714	0.2760175	-2.655976
ENST0000C	1.7524476	25.521533	3.6690357	0.001135	0.1421743	-0.793824
lincRNA:ch	1.7523692	9.8392	2.302779	0.0297736	0.399347	-3.583291
HCG27	1.7517231	22.41365	2.4798088	0.0201377	0.3519698	-3.257862
A_32_P756	1.7498238	18.898367	2.6433088	0.0138855	0.3090972	-2.945196
FKBP5	1.7497552	28.604275	2.7946109	0.0097633	0.2751492	-2.64661
ATP8B3	1.745986	11.023833	2.1087856	0.0450129	0.4510888	-3.922376
SPAG1	1.7456825	9.7103417	2.0972634	0.046107	0.4550676	-3.941895
lincRNA:ch	1.7437343	7.07805	3.3110786	0.0027937	0.1870461	-1.573373
lincRNA:ch	1.7414434	14.7945	2.153143	0.0410143	0.4392951	-3.846566
SCPEP1	1.7407972	26.64795	3.3983049	0.0022478	0.1688885	-1.385551
CCDC138	1.7402881	14.512983	2.4891028	0.0197218	0.3487739	-3.240388
LOC100131	1.7376056	11.839567	2.3183258	0.0287832	0.3979204	-3.555297
CES8	1.736607	13.358917	2.8724306	0.0081227	0.2579482	-2.489901
LOC100134	1.7350406	19.18945	2.1958249	0.0374708	0.425099	-3.772636

SKP2	1.734972	20.717958	3.6850687	0.0010896	0.1399536	-0.758426
C9orf47	1.7339179	11.949739	3.5184547	0.0016622	0.1530728	-1.124444
LOC100129	1.7325147	24.0779	2.2335118	0.0345741	0.4173873	-3.706573
IRGM	1.7297669	13.116872	2.2337283	0.0345581	0.4173873	-3.706192
lincRNA:ch	1.7294406	16.831617	2.7838414	0.0100136	0.27687	-2.668135
ENST00000	1.7291273	24.852683	4.5148725	0.0001275	0.0677403	1.1029271
GAB2	1.7286671	35.872725	3.5572101	0.0015073	0.1515078	-1.039687
CXorf41	1.7269343	6.32415	2.1763479	0.0390521	0.4318941	-3.806491
lincRNA:ch	1.7266993	11.31515	2.7715708	0.0103061	0.2801815	-2.692612
AK022213	1.725172	16.60225	2.347229	0.0270214	0.390281	-3.502946
CHPT1	1.7241401	24.203258	4.2554518	0.0002505	0.0890075	0.5173108
lincRNA:ch	1.7235077	9.0031667	2.2410715	0.034018	0.4150494	-3.693235
DDX17	1.722979	25.650917	2.2448617	0.0337423	0.4147478	-3.686536
CAPN3	1.7210308	22.296225	3.2089135	0.0035969	0.2061571	-1.791276
lincRNA:ch	1.7188867	30.5263	3.240639	0.0033262	0.200491	-1.723865
CHRNA7	1.7172126	16.384375	2.5321034	0.0178991	0.3363198	-3.159058
lincRNA:ch	1.7164196	6.0718	2.507247	0.0189327	0.3454014	-3.206166
H2AFV	1.715049	26.764967	3.16161	0.0040402	0.2107	-1.891339
lincRNA:ch	1.712621	27.368833	2.7593442	0.0106056	0.2829436	-2.716948
lincRNA:ch	1.7109762	20.384233	3.0791161	0.0049417	0.2225409	-2.064487
lincRNA:ch	1.7098014	22.502433	2.9618831	0.0065601	0.2412091	-2.307348
ZBTB7B	1.709449	25.889733	2.6201913	0.0146433	0.3149228	-2.99006
lincRNA:ch	1.7090378	25.680783	3.2994607	0.0028755	0.1891324	-1.59827
LOC729799	1.7066098	29.60685	2.3536746	0.0266422	0.3883799	-3.491217
lincRNA:ch	1.7059636	6.6325	2.0793488	0.0478553	0.4593556	-3.972098
TUSC3	1.7056601	5.7334083	2.5064126	0.0189683	0.3454014	-3.207743
lincRNA:ch	1.704593	29.329067	3.2975112	0.0028894	0.1891663	-1.602445
KDM2A	1.7018713	27.596683	2.6413081	0.0139496	0.3091811	-2.949087
IDS	1.7007944	30.0762	4.246048	0.0002567	0.0897287	0.4961084
ENST00000	1.7001483	16.81295	2.6193388	0.014672	0.3151637	-2.991711
lincRNA:ch	1.698386	5.85305	2.221999	0.0354368	0.4203608	-3.726831
SCN4B	1.6982294	14.973583	2.2273818	0.035031	0.4189229	-3.717368
FETUB	1.6975049	11.8965	2.5354052	0.0177658	0.3355597	-3.152781
A_33_P342	1.6964671	16.148883	2.096705	0.0461606	0.4551037	-3.942839
lincRNA:ch	1.6959385	16.805833	3.548365	0.0015413	0.1515078	-1.059052
TGIF2	1.6952531	17.581317	2.755763	0.0106949	0.2835933	-2.724066
PABPC1L	1.6951455	16.973658	3.6364436	0.001233	0.1452138	-0.865671
FOXK1	1.6942937	23.891233	2.4110339	0.0234763	0.3724479	-3.385992
LOC286052	1.6922182	27.7984	4.8849252	4.86E-05	0.064665	1.9372627
lincRNA:ch	1.6894867	23.658775	3.4904363	0.0017838	0.1574654	-1.185564
lincRNA:ch	1.6887133	7.3022833	2.7460148	0.0109415	0.2862232	-2.743418
HOXA6	1.6857762	11.500183	3.0170755	0.0057436	0.2312816	-2.1935
ZNF516	1.6850713	30.292383	2.615101	0.0148153	0.3167176	-2.999911

lincRNA:ch	1.6805972	19.927542	2.3191627	0.0287307	0.3975098	-3.553787
ZXDC	1.6774545	23.344767	3.0237769	0.0056513	0.2304636	-2.179617
TREM1	1.676593	36.2285	2.4132846	0.0233594	0.3722428	-3.381832
IL13RA1	1.675751	35.50645	3.7999784	0.0008124	0.1315436	-0.503778
lincRNA:ch	1.6754769	18.13595	3.0035167	0.0059347	0.2340979	-2.221551
PNMAL2	1.6752028	12.537583	3.0449208	0.0053694	0.2266983	-2.135729
DDIT3	1.6743413	25.943517	3.079325	0.0049392	0.2225409	-2.064051
LOC731789	1.6717958	10.907283	2.1422061	0.0419694	0.4421393	-3.865356
lincRNA:ch	1.6712671	18.044833	2.1634593	0.0401313	0.4356864	-3.828785
A_33_P321	1.6711692	6.4240167	2.4033326	0.0238802	0.3753814	-3.400208
USP46	1.6697203	14.83825	2.8000066	0.0096401	0.2739115	-2.63581
A_33_P329	1.6679189	18.186583	3.0858923	0.0048609	0.2225409	-2.050332
PCBP3	1.6676252	10.563933	2.4924145	0.0195755	0.3479565	-3.234152
SH3BP2	1.6656606	16.884039	2.9017812	0.0075747	0.2548004	-2.430279
lincRNA:ch	1.664414	7.1180667	2.3573228	0.0264297	0.3876175	-3.48457
KIAA0895	1.664042	6.6680833	3.0060781	0.0058981	0.2337109	-2.216256
lincRNA:ch	1.6625734	13.916233	3.095199	0.004752	0.2225409	-2.030871
IKZF5	1.6610462	18.676933	3.4283957	0.0020847	0.1652092	-1.320407
ENST0000C	1.6608112	21.145133	3.7228346	0.0009896	0.1387497	-0.674914
PAQR5	1.6601063	13.793733	2.0731936	0.0484695	0.4605658	-3.982434
POSTN	1.659049	6.7258333	2.468703	0.0206453	0.3556102	-3.278694
C9orf122	1.6582853	16.401583	2.5712142	0.0163782	0.3267141	-3.084413
RAPH1	1.6582462	21.775017	2.6864497	0.0125683	0.3010286	-2.86092
GPER	1.6581221	21.990422	2.487581	0.0197894	0.3487739	-3.243252
LOC100291	1.6578937	15.255917	2.5521553	0.0171037	0.332813	-3.120867
DKFZp686	1.6570126	19.165767	2.820763	0.0091798	0.2686977	-2.594173
MXI1	1.6543379	21.980863	2.4167073	0.0231827	0.3706367	-3.375502
A_33_P334	1.6543105	12.551467	2.230157	0.0348235	0.4182176	-3.712483
MICALL2	1.6539972	23.602133	3.1440961	0.0042173	0.2139793	-1.928246
ZBED1	1.6516182	25.852808	2.4791932	0.0201655	0.3519698	-3.259018
ELL2	1.6510406	28.15715	3.3656777	0.0024387	0.1726719	-1.455988
lincRNA:ch	1.6494741	18.145283	2.788448	0.0099058	0.2760175	-2.658933
FBXO24	1.6491902	13.971475	3.06281	0.0051414	0.2240985	-2.098499
lincRNA:ch	1.6486909	10.74675	2.0865151	0.047149	0.4580245	-3.960037
GABARAP	1.6483189	30.5921	2.2874059	0.0307834	0.4029988	-3.610857
KIT	1.6475716	15.543519	2.3434115	0.0272483	0.3909279	-3.509883
MYB	1.6419748	28.073967	2.0714489	0.0486449	0.4611038	-3.985361
ENST0000C	1.6408196	12.86845	2.7215856	0.0115832	0.2935077	-2.791766
lincRNA:ch	1.6400951	12.283367	2.4659837	0.0207714	0.3570641	-3.283786
lincRNA:ch	1.6397818	26.705233	3.2732506	0.0030685	0.192881	-1.654329
LOC727938	1.6384308	13.151483	2.3620661	0.0261558	0.3875029	-3.475918
RBM3	1.6334117	33.164522	3.2934919	0.0029184	0.1892297	-1.611049
A_33_P339	1.6320084	22.83855	2.5302944	0.0179725	0.3371555	-3.162495

ATP13A1	1.6315776	24.229917	3.1667177	0.0039899	0.2107	-1.880561
SYAP1	1.6268979	28.7924	2.2985032	0.0300514	0.3999487	-3.59097
C9orf57	1.6258993	5.82855	3.2312578	0.0034041	0.2037179	-1.743823
LOC730974	1.6252727	5.9414833	2.3343206	0.0277957	0.3929735	-3.526376
SLC39A7	1.6248811	24.268417	2.077326	0.0480564	0.4593556	-3.975497
LCOR	1.6247441	22.138433	2.2460511	0.0336562	0.4142947	-3.684433
WDFY3	1.6234615	24.900575	2.2707437	0.0319129	0.4060157	-3.640603
LOC100131	1.6228839	6.0369167	3.6550692	0.001176	0.1430858	-0.82463
lincRNA:ch	1.6215524	6.02805	2.3286324	0.0281432	0.3953289	-3.536675
ZNF674	1.6206713	13.4323	2.8155536	0.0092933	0.2694363	-2.604637
lincRNA:ch	1.6203776	16.70585	2.3584483	0.0263645	0.3875594	-3.482518
FAM65A	1.6176364	33.071383	2.064291	0.0493703	0.4632547	-3.997348
lincRNA:ch	1.6172056	28.97055	2.5345423	0.0178005	0.3355597	-3.154422
FAM55C	1.6150517	21.441583	2.0659054	0.0492058	0.4630302	-3.994647
NFRKB	1.6149734	16.75765	2.5772944	0.0161529	0.3248146	-3.072752
CXorf65	1.6143273	29.8081	2.6663265	0.0131674	0.3043575	-2.900319
STK17B	1.6131231	34.048175	3.2078079	0.0036067	0.2061571	-1.793621
MSRB3	1.6112336	21.619733	2.6213613	0.0146041	0.3149228	-2.987795
DUSP16	1.6110573	19.692983	3.0149198	0.0057736	0.2319309	-2.197963
lincRNA:ch	1.6105874	25.841783	3.0404385	0.005428	0.2266983	-2.145043
UNQ2963	1.6094517	13.25695	3.1141134	0.0045379	0.2206763	-1.991248
lincRNA:ch	1.6091483	25.201458	2.3224077	0.0285282	0.3971357	-3.547928
KRTAP9-8	1.6086685	7.6252167	2.387218	0.0247463	0.3809379	-3.429866
NFIL3	1.6080811	38.253717	3.5456457	0.001552	0.1515078	-1.065003
AMY1C	1.6079571	25.884056	2.5452869	0.0173724	0.3344367	-3.133967
lincRNA:ch	1.6075916	14.199033	2.4959088	0.0194223	0.3468525	-3.227567
GRK6	1.6062161	34.211479	2.6715599	0.0130091	0.3029786	-2.890087
A_33_P323	1.6057706	18.106083	2.6982323	0.0122295	0.2964076	-2.837781
ERN1	1.6054867	18.034275	3.4160526	0.0021501	0.1687148	-1.34715
CRYZL1	1.604586	19.292642	2.2549986	0.0330148	0.4111299	-3.668586
lincRNA:ch	1.6043413	26.7064	3.3959785	0.0022609	0.1688885	-1.390581
PHC2	1.6037538	34.5247	2.4231417	0.0228538	0.3685825	-3.363588
NLRP1	1.6031762	27.161342	2.7020727	0.012121	0.2962939	-2.830228
MYT1L	1.6021678	6.55235	3.0126031	0.0058059	0.2321647	-2.202759
MAPK8IP3	1.599407	36.8298	2.9269436	0.007133	0.2495396	-2.378948
COQ10B	1.5992521	21.848814	3.1005055	0.004691	0.2225409	-2.019764
CD59	1.5991035	16.145908	2.3336092	0.0278389	0.3931293	-3.527664
lincRNA:ch	1.5967441	22.562867	3.2044123	0.003637	0.2061571	-1.800821
ZNF815	1.5935524	25.988083	3.4478152	0.0019856	0.1624754	-1.278274
ENST0000C	1.591379	17.851633	2.3162641	0.0289128	0.3982523	-3.559016
lincRNA:ch	1.5907916	29.659933	3.4740309	0.001859	0.1603466	-1.221288
A_33_P321	1.5905762	6.3554167	2.4917926	0.0196029	0.3481216	-3.235324
SAT1	1.589297	43.284772	3.1649099	0.0040076	0.2107	-1.884377

PTGER2	1.589049	30.552317	3.1723709	0.0039349	0.2096368	-1.868624
lincRNA:ch	1.5881483	19.118283	2.0684545	0.0489472	0.4622301	-3.990379
LOC100288	1.5880112	12.2661	2.4534447	0.021362	0.3596244	-3.307227
EIF2C4	1.586249	30.4738	3.550728	0.0015322	0.1515078	-1.05388
IRAK3	1.5856517	26.118458	2.5607288	0.0167737	0.3294068	-3.104487
DTX4	1.5837818	12.7505	3.0144294	0.0057804	0.2319392	-2.198979
GPSM2	1.5828028	17.244733	3.0802224	0.0049284	0.2225409	-2.062177
NOTCH1	1.579953	26.287768	2.0747736	0.0483112	0.4603129	-3.979783
TSHZ3	1.5799049	28.7868	2.7655233	0.0104532	0.2820898	-2.704655
AGAP7	1.5784608	21.728554	2.3890808	0.0246447	0.3803859	-3.426444
A_33_P324	1.5781231	11.105617	2.1505609	0.041238	0.4403384	-3.851008
SIK2	1.5778294	17.562767	2.4775621	0.0202395	0.3524409	-3.262081
ENST0000C	1.5777706	21.873717	2.9704686	0.0064262	0.2393269	-2.289697
RNASE12	1.5771832	12.413217	2.8209529	0.0091757	0.2686977	-2.593791
A_33_P334	1.5753035	8.6108167	2.2584249	0.0327721	0.4102265	-3.662507
ENST0000C	1.575186	14.499917	2.1430295	0.0418968	0.4421393	-3.863944
lincRNA:ch	1.5733259	6.3	2.131068	0.0429626	0.4451527	-3.884426
METT11D1	1.5728951	24.359767	2.7220024	0.0115719	0.2935077	-2.790943
A_33_P327	1.5726601	18.987967	3.6540427	0.0011791	0.1430858	-0.826893
lincRNA:ch	1.5725818	28.950833	2.4880919	0.0197667	0.3487739	-3.24229
PER3	1.5708	12.850717	2.3147066	0.029011	0.398859	-3.561824
PELI1	1.5679804	39.30605	3.1828512	0.003835	0.2071534	-1.846474
SPTBN1	1.5667958	14.743108	2.867847	0.0082116	0.2581722	-2.499186
ENST0000C	1.566179	7.3267833	2.1636973	0.0401111	0.4356864	-3.828374
GABBR1	1.5642732	18.531606	2.8360682	0.0088538	0.2648239	-2.563377
CTDSP2	1.5640839	33.698467	3.5979186	0.0013597	0.1482829	-0.950399
PAGE1	1.5628503	5.5798167	2.6407353	0.013968	0.3091988	-2.9502
ENST0000C	1.5621259	17.547133	3.0537573	0.0052556	0.2245871	-2.11735
SPATA6	1.5607455	16.205058	3.7502569	0.0009226	0.1345858	-0.614161
NAMPT	1.5602984	39.062956	3.095411	0.0047496	0.2225409	-2.030427
lincRNA:ch	1.5596881	11.508758	2.0864918	0.0471513	0.4580245	-3.960076
RBM6	1.5594434	26.398517	2.9451362	0.006829	0.2432702	-2.341713
CR591103	1.5582685	12.643517	2.4632642	0.0208982	0.3574747	-3.288876
A_33_P331	1.5577399	14.521267	2.2459671	0.0336623	0.4142947	-3.684581
IP6K3	1.5577007	18.9371	2.7736569	0.0102558	0.2800238	-2.688454
TMEM88	1.5564671	27.09945	2.0683072	0.0489621	0.4622301	-3.990625
TRPV3	1.5555664	5.9844167	2.2087313	0.036455	0.4224547	-3.750094
ENST0000C	1.5541958	13.653383	2.1085745	0.0450327	0.4510888	-3.922735
LOC100128	1.5530993	11.072717	2.4983354	0.0193165	0.3462109	-3.222992
A_33_P328	1.5523161	16.601783	2.0719195	0.0485975	0.4609684	-3.984571
OVGP1	1.5506517	16.2463	2.5415317	0.0175209	0.334977	-3.141121
SBDSP	1.5487524	22.301417	3.2292764	0.0034208	0.2043662	-1.748035
LOC100130	1.5462462	10.81395	2.0724255	0.0485467	0.4609285	-3.983723

KDM2B	1.5433776	18.318942	2.2912527	0.0305278	0.4012472	-3.60397
A_33_P341·	1.5424867	12.79355	2.4175737	0.0231382	0.3704211	-3.373899
lincRNA:ch	1.5414098	29.833767	3.3816194	0.0023435	0.1705453	-1.421599
XRCC5	1.538835	32.045008	2.1386768	0.0422819	0.4430089	-3.871406
STRN	1.5386098	15.76785	3.804124	0.0008038	0.1315436	-0.494562
TMPPE	1.538042	21.101033	4.0712845	0.000404	0.1093626	0.102758
MMP9	1.5372666	42.397133	2.0640361	0.0493963	0.4633253	-3.997774
ALPL	1.535849	27.5037	2.4731632	0.0204401	0.3541692	-3.270334
SYF2	1.5355161	25.832683	3.6867571	0.0010849	0.1399536	-0.754697
lincRNA:ch	1.5344	12.817933	2.522091	0.018309	0.3390991	-3.178065
A_33_P334(	1.5333622	20.564717	2.6564511	0.0134711	0.3058213	-2.919597
ARHGAP1.	1.5328727	30.013433	3.3258688	0.0026929	0.183458	-1.541637
KIAA0556	1.5316979	16.650433	2.9913996	0.0061106	0.2368074	-2.246574
ENST0000C	1.5311497	23.6873	2.3543827	0.0266008	0.3883799	-3.489927
A_33_P332!	1.5308364	37.974767	2.4456808	0.0217354	0.3614175	-3.321706
CBLN3	1.5288392	17.725867	2.3823165	0.0250154	0.3817743	-3.438864
NXF1	1.5276252	30.6159	2.8681159	0.0082063	0.2581722	-2.498642
GMEB2	1.5272531	23.081917	3.1033966	0.004658	0.2220927	-2.01371
lincRNA:ch	1.5239636	36.393117	2.4700165	0.0205847	0.3554645	-3.276233
SON	1.5227497	20.58931	2.3831694	0.0249684	0.3813898	-3.437299
LOC222159	1.5223189	21.103717	2.8754818	0.008064	0.2574548	-2.483715
lincRNA:ch	1.5215748	16.26135	2.9893789	0.0061404	0.2370945	-2.250743
KCNMB4	1.5206643	22.251075	2.7511611	0.0108107	0.2849344	-2.733206
C17orf102	1.5203021	10.807533	2.4995197	0.0192651	0.3462109	-3.220758
FAM114A1	1.5202238	12.5776	2.4880206	0.0197698	0.3487739	-3.242424
ENST0000C	1.5178937	13.696083	2.2115695	0.036235	0.4217107	-3.745125
ZMYM5	1.5172182	20.721808	3.3425965	0.0025831	0.1784419	-1.505687
SOCS7	1.5171692	10.7114	2.1854608	0.0383049	0.4281054	-3.790675
MPZL1	1.5169636	28.199325	2.4641944	0.0208547	0.3573357	-3.287135
SNHG3	1.5158573	33.607817	2.8376517	0.0088207	0.2643867	-2.560187
lincRNA:ch	1.5157986	27.527967	3.596031	0.0013662	0.1482829	-0.954545
ENST0000C	1.5149958	9.73455	2.2070698	0.0365844	0.4224608	-3.753
lincRNA:ch	1.5140364	13.054067	2.3518924	0.0267465	0.3885822	-3.494462
LOC10013C	1.513449	5.7215667	2.1362049	0.042502	0.4431046	-3.875639
FLJ31306	1.5118042	23.939767	3.1725239	0.0039334	0.2096368	-1.868301
A_33_P341:	1.5117259	25.575433	2.1427899	0.0419179	0.4421393	-3.864355
lincRNA:ch	1.5104336	12.896333	3.2088176	0.0035977	0.2061571	-1.791479
A_33_P333·	1.5101399	30.665483	3.7347281	0.00096	0.1367135	-0.648576
INPP5A	1.5090923	22.618225	5.1609161	2.37E-05	0.0590814	2.555524
FLYWCH1	1.508172	18.693908	2.533493	0.0178429	0.3356245	-3.156417
lincRNA:ch	1.5075552	24.302483	2.5014356	0.0191822	0.3457445	-3.217142
KIAA0895I	1.5071245	20.35425	2.5257016	0.0181602	0.3388496	-3.171216
A_33_P321'	1.5068503	29.780683	2.4018546	0.0239585	0.3756903	-3.402933

LOC285084	1.5047944	11.305933	2.2288147	0.0349237	0.4185103	-3.714846
lincRNA:ch	1.5044028	29.376667	3.0620418	0.005151	0.2240985	-2.1001
TLR8	1.5039622	30.985792	2.5623099	0.0167135	0.3294068	-3.101463
MGAT4A	1.5036196	30.739333	2.9753292	0.0063515	0.2381763	-2.279694
lincRNA:ch	1.5032867	16.546717	2.7679618	0.0103937	0.2811016	-2.699801
UBXN10	1.5031301	9.45665	2.1801385	0.0387397	0.4309086	-3.799917
8-Mar	1.5024643	17.307617	3.2646265	0.0031347	0.1955602	-1.672741
PLCL2	1.5001441	27.689142	3.6997457	0.0010496	0.1394762	-0.725993
TRPM6	1.5000912	17.799997	2.560473	0.0167835	0.3294068	-3.104976
lincRNA:ch	-1.500173	41.060133	-2.63876	0.0140316	0.3100369	-2.95404
HIST3H2A	-1.500741	13.185317	-2.242149	0.0339395	0.4150494	-3.691331
lincRNA:ch	-1.501152	14.628367	-2.321541	0.0285821	0.3971357	-3.549493
USP53	-1.502523	21.852133	-2.545837	0.0173507	0.3344367	-3.132918
RRAS2	-1.503241	29.146678	-2.76903	0.0103677	0.2806665	-2.697673
TMEM39A	-1.503443	21.397017	-3.234129	0.0033801	0.2027527	-1.737716
A_33_P336.	-1.505773	20.1663	-2.077044	0.0480845	0.4593556	-3.975971
VCX2	-1.506126	12.6014	-2.28109	0.0312072	0.4049651	-3.622149
UPK3A	-1.507526	17.042842	-2.112005	0.0447114	0.4501553	-3.91691
NPHS2	-1.508162	20.886133	-2.161655	0.0402845	0.4359062	-3.831898
BOLA1	-1.508358	25.7005	-4.688231	8.11E-05	0.064665	1.4942513
GALNT3	-1.508642	24.934292	-2.244396	0.0337761	0.4147716	-3.68736
TTC38	-1.510375	28.191917	-2.114585	0.044471	0.4490226	-3.912525
ZBTB24	-1.510414	26.56535	-3.052831	0.0052674	0.2247746	-2.119277
LOC100293	-1.511207	35.936775	-2.113628	0.04456	0.4493159	-3.914152
KIR2DS4	-1.512444	18.637111	-2.596212	0.0154701	0.3193262	-3.036376
GZMM	-1.512734	34.376475	-2.449984	0.0215277	0.361196	-3.313683
KIAA1211	-1.513273	6.5567833	-2.061958	0.0496087	0.4634338	-4.001248
EED	-1.513694	32.261192	-3.708004	0.0010277	0.1387497	-0.70773
GMPR	-1.513723	23.460267	-2.108088	0.0450784	0.4511166	-3.923559
MRPS14	-1.514389	30.496433	-3.227382	0.0034368	0.2045187	-1.752063
lincRNA:ch	-1.515309	6.2609167	-2.28974	0.0306281	0.4019604	-3.606679
lincRNA:ch	-1.515524	11.5248	-2.185322	0.0383162	0.4281054	-3.790916
CCDC28B	-1.515857	23.428183	-3.55132	0.0015299	0.1515078	-1.052584
NCRNA000	-1.516053	12.34415	-2.4786	0.0201924	0.3521474	-3.260131
ABHD6	-1.517463	26.60175	-3.702384	0.0010426	0.1394762	-0.72016
LOC400743	-1.517786	18.130175	-2.506401	0.0189688	0.3454014	-3.207766
KNCN	-1.519206	10.971333	-2.893108	0.0077328	0.2558585	-2.447927
FANCF	-1.51968	27.550513	-2.941636	0.0068865	0.2442486	-2.348884
LOC100130	-1.51995	14.787967	-4.099884	0.0003751	0.1057281	0.1670221
ZNF35	-1.520792	16.888783	-2.150261	0.0412641	0.4403474	-3.851523
lincRNA:ch	-1.521575	18.34105	-2.260447	0.0326297	0.4098389	-3.658917
lincRNA:ch	-1.522808	15.071	-2.764573	0.0104765	0.2820898	-2.706547
CCDC117	-1.523376	29.576983	-2.879464	0.007988	0.2571214	-2.475638

ENTPD8	-1.523885	11.321217	-2.92212	0.0072157	0.2503341	-2.388803
GPAM	-1.523905	20.825933	-2.624835	0.0144881	0.3139876	-2.981065
lincRNA:ch	-1.526	16.791017	-3.025562	0.005627	0.2303131	-2.175916
ENST0000C	-1.528076	5.9197833	-2.613731	0.0148619	0.3167176	-3.00256
MMP26	-1.529681	7.76755	-2.474073	0.0203984	0.3540935	-3.268629
ENST0000C	-1.53066	30.045983	-2.825389	0.0090801	0.2675717	-2.584873
FAT2	-1.531326	12.67315	-2.957577	0.0066283	0.2412766	-2.316191
lincRNA:ch	-1.532344	23.277217	-2.350407	0.0268338	0.3893928	-3.497166
lincRNA:ch	-1.534557	6.6374	-2.526055	0.0181457	0.3388496	-3.170545
CCL15	-1.535134	20.726942	-2.425725	0.022723	0.3678645	-3.358799
C1orf111	-1.53581	12.953267	-3.217078	0.0035253	0.2056835	-1.773951
GADD45G	-1.536456	27.498917	-2.171541	0.0394515	0.4326859	-3.814815
GAS1	-1.536603	14.806692	-3.495939	0.0017593	0.1572829	-1.173571
lincRNA:ch	-1.536613	14.55825	-2.879559	0.0079862	0.2571214	-2.475447
SPINLW1	-1.536789	8.4595	-2.65923	0.013385	0.305256	-2.914177
TNFRSF19	-1.536975	12.354708	-2.206832	0.036603	0.4224608	-3.753417
lincRNA:ch	-1.537964	11.7145	-2.083415	0.0474534	0.4592922	-3.965258
lincRNA:ch	-1.538629	15.442467	-2.086322	0.0471679	0.4580245	-3.960362
TMEM231	-1.540294	12.992583	-2.475172	0.0203482	0.3537766	-3.266566
NAT1	-1.542281	25.767525	-2.272194	0.0318131	0.4058114	-3.638019
EN1	-1.543505	8.1747167	-2.307246	0.0294859	0.399347	-3.57526
SH3RF2	-1.544131	14.43785	-2.11108	0.0447979	0.4501553	-3.918482
LOC646588	-1.544151	11.115767	-3.134734	0.004315	0.2154134	-1.947943
A_33_P339	-1.544464	5.6128333	-2.502432	0.0191392	0.3456829	-3.215262
lincRNA:ch	-1.545443	20.707867	-2.343828	0.0272235	0.3907322	-3.509127
CHRNB1	-1.547284	20.370233	-2.592224	0.0156118	0.3201973	-3.044056
EPC2	-1.549418	30.26975	-2.988074	0.0061597	0.2370945	-2.253434
ZNF454	-1.551533	5.58915	-2.173403	0.0392964	0.4320798	-3.811593
CYP2U1	-1.55351	23.306733	-3.685663	0.0010879	0.1399536	-0.757114
ZNF232	-1.554685	20.572533	-2.987605	0.0061667	0.2370945	-2.254401
SC5DL	-1.555322	11.337025	-2.425904	0.022714	0.3678645	-3.358468
C20orf134	-1.556448	24.519833	-4.021587	0.0004593	0.1111414	-0.00879
LOC100129	-1.557799	23.249683	-2.0651	0.0492878	0.4632547	-3.995995
lincRNA:ch	-1.559169	11.86465	-2.157773	0.0406158	0.4373021	-3.838592
C3orf20	-1.559189	11.902567	-2.68251	0.0126836	0.3011401	-2.868645
ZNF236	-1.559483	19.600117	-2.543561	0.0174405	0.3346187	-3.137257
lincRNA:ch	-1.559678	11.453283	-2.272962	0.0317604	0.4057901	-3.63665
DARS2	-1.560931	18.59235	-2.295298	0.0302611	0.4005965	-3.596719
lincRNA:ch	-1.562185	23.628617	-2.529214	0.0180165	0.3376943	-3.164547
CB962925	-1.562194	14.379575	-3.532355	0.0016049	0.1530728	-1.094073
CCDC27	-1.562792	9.6056333	-2.100905	0.0457587	0.4538878	-3.935734
RARRES3	-1.562958	39.501525	-3.390827	0.0022902	0.1688885	-1.401713
MAG	-1.563575	21.1659	-2.125448	0.0434717	0.4460421	-3.894023

THEG	-1.564201	18.529233	-2.104837	0.0453852	0.4524151	-3.929073
FLNC	-1.566678	6.9274917	-2.352389	0.0267174	0.3884394	-3.493558
lincRNA:ch	-1.568313	21.247333	-4.694395	7.98E-05	0.064665	1.5081553
LOC100129	-1.568607	21.550083	-3.391527	0.0022862	0.1688885	-1.400202
lincRNA:ch	-1.569018	19.526733	-2.668343	0.0131062	0.3039688	-2.896377
TMPRSS11	-1.569155	6.23735	-2.570099	0.0164199	0.3270385	-3.08655
lincRNA:ch	-1.569253	18.853333	-2.996359	0.006038	0.23565	-2.236337
LOC400043	-1.569782	18.331483	-3.846619	0.0007208	0.1278826	-0.399985
ENST00000	-1.572092	10.85525	-2.718253	0.0116734	0.2935077	-2.798345
NCRNA000	-1.574873	19.560217	-2.726645	0.0114474	0.2935077	-2.78177
PPIAL4A	-1.575744	35.348308	-3.019539	0.0057095	0.2306829	-2.188398
ZNF322A	-1.576243	28.892383	-3.249193	0.0032567	0.1995927	-1.70565
FAM98A	-1.577164	20.717667	-2.31111	0.0292391	0.398859	-3.568305
lincRNA:ch	-1.577457	11.046817	-2.888067	0.0078262	0.2563428	-2.458171
CALHM2	-1.578241	31.578283	-4.50826	0.0001297	0.0677403	1.0879944
HSFX1	-1.579513	15.182067	-3.713447	0.0010136	0.1387497	-0.695689
SLC7A3	-1.581667	8.7031	-2.655102	0.0135131	0.3058744	-2.922228
lincRNA:ch	-1.581961	12.91605	-2.479563	0.0201488	0.3519698	-3.258323
ACSM5	-1.582509	16.508917	-2.334391	0.0277914	0.3929735	-3.526247
lincRNA:ch	-1.583371	12.15165	-3.280587	0.0030133	0.1921729	-1.638654
A_33_P323	-1.584369	16.4206	-2.46193	0.0209606	0.3574747	-3.291372
lincRNA:ch	-1.584448	6.3214667	-2.197679	0.0373233	0.4250149	-3.769402
OSMR	-1.585936	11.377333	-2.861173	0.0083427	0.2601276	-2.512694
SNORA76	-1.586288	15.698433	-2.090882	0.0467231	0.4559972	-3.952673
LOC339803	-1.586484	23.0804	-2.673058	0.0129641	0.3029433	-2.887157
lincRNA:ch	-1.586778	17.72715	-2.372185	0.0255801	0.3843614	-3.457427
A_33_P340	-1.587835	10.36385	-2.199114	0.0372095	0.4247015	-3.766899
ENHO	-1.588109	21.475883	-2.638682	0.0140341	0.3100369	-2.954191
GCNT1	-1.592769	21.84945	-2.602325	0.0152553	0.3175738	-3.02459
lincRNA:ch	-1.593552	13.861517	-2.220692	0.035536	0.4209987	-3.729128
BC015720	-1.593846	6.0302667	-2.114052	0.0445206	0.4490479	-3.913432
DCP_22_9	-1.594101	11.022783	-2.149417	0.0413374	0.4406177	-3.852974
PHLPP2	-1.5974	16.731342	-3.273994	0.0030629	0.1928731	-1.65274
lincRNA:ch	-1.59833	20.705183	-3.683019	0.0010953	0.1399536	-0.762954
TBX21	-1.598428	36.811367	-2.077661	0.048023	0.4593556	-3.974934
C12orf60	-1.599622	15.082083	-2.328875	0.0281283	0.3952781	-3.536236
KCNA10	-1.599642	13.1292	-2.567835	0.0165047	0.3274087	-3.090888
lincRNA:ch	-1.601659	8.2706167	-2.638133	0.0140519	0.3101914	-2.955258
PALLD	-1.602448	21.784856	-3.007274	0.0058811	0.2334111	-2.213783
ZNF879	-1.605281	18.9728	-2.62365	0.0145275	0.3143793	-2.983361
LOC284263	-1.606241	6.4499167	-2.737745	0.0111549	0.2889928	-2.759808
OR10C1	-1.607102	18.07505	-3.260316	0.0031683	0.1963303	-1.681937
DGCR5	-1.607885	7.2941167	-2.223613	0.0353147	0.419515	-3.723996

MRM1	-1.608355	23.024517	-4.177031	0.0003071	0.0992932	0.3405977
lincRNA:ch	-1.609197	9.0673333	-2.085896	0.0472097	0.4580245	-3.961081
SAC3D1	-1.611743	26.7085	-3.032175	0.0055377	0.2292353	-2.1622
ZNF107	-1.612076	25.513483	-2.889722	0.0077954	0.2563428	-2.454808
PDE8B	-1.614327	17.1857	-2.175872	0.0390915	0.4318941	-3.807315
ZNF713	-1.615159	20.829958	-2.295089	0.0302748	0.4005965	-3.597094
AK309505	-1.615345	15.972367	-2.636653	0.0140998	0.3105066	-2.958134
SYT17	-1.617127	19.949183	-2.366539	0.0258999	0.385838	-3.467751
LOC100131	-1.61889	7.9728833	-2.168074	0.0397418	0.4344328	-3.820812
LOC650095	-1.619164	6.7285167	-2.14002	0.0421628	0.442736	-3.869105
PIGV	-1.619731	23.9253	-3.043574	0.0053869	0.2266983	-2.138528
LOC100131	-1.620495	13.46205	-2.614856	0.0148236	0.3167176	-3.000385
TNFAIP8L	-1.621063	33.366433	-2.730158	0.0113541	0.2926356	-2.774826
CPXM2	-1.622013	10.230792	-2.274666	0.0316437	0.4057901	-3.633613
C6orf138	-1.623256	7.0455	-2.152089	0.0411055	0.4397291	-3.84838
EDN3	-1.62519	13.373471	-2.7016	0.0121343	0.2962939	-2.831157
AMTN	-1.625234	9.6570833	-2.719085	0.0116508	0.2935077	-2.796702
SUGT1L1	-1.625723	12.8562	-2.156181	0.0407525	0.4382868	-3.841336
CCR9	-1.62727	18.121017	-2.224683	0.035234	0.419515	-3.722115
RTN4R	-1.627466	20.929183	-2.703777	0.0120731	0.2962939	-2.826873
ZNF555	-1.629179	23.329892	-3.145943	0.0041982	0.2139793	-1.924357
LOC727844	-1.629776	13.80855	-2.073525	0.0484362	0.4605658	-3.981878
HYLS1	-1.630207	21.278717	-3.202632	0.0036529	0.2061571	-1.804595
lincRNA:ch	-1.630951	6.72315	-2.096573	0.0461734	0.4551037	-3.943063
GRB7	-1.631	20.000342	-2.545149	0.0173778	0.3344367	-3.134231
C19orf53	-1.634573	16.719733	-3.103837	0.004653	0.2220927	-2.012788
LOC729681	-1.636492	5.6123667	-2.549289	0.0172153	0.3335135	-3.126336
APOA4	-1.636698	9.9332917	-3.209007	0.003596	0.2061571	-1.791078
lincRNA:ch	-1.637276	9.6742333	-2.139367	0.0422206	0.442736	-3.870223
GPR68	-1.640007	30.626808	-2.454199	0.021326	0.3593651	-3.305818
BATF	-1.640614	36.507625	-3.92671	0.0005867	0.1179671	-0.221245
lincRNA:ch	-1.641779	37.043067	-2.085799	0.0472192	0.4580245	-3.961243
lincRNA:ch	-1.641838	40.553217	-2.285813	0.0308898	0.4033224	-3.613707
lincRNA:ch	-1.642347	17.62705	-2.387086	0.0247535	0.3809379	-3.430108
lincRNA:ch	-1.642425	37.123917	-2.224538	0.0352449	0.419515	-3.72237
DPPA3	-1.643561	5.9190833	-2.290419	0.030583	0.4015677	-3.605462
lincRNA:ch	-1.643717	14.678417	-2.743404	0.0110085	0.2875444	-2.748597
lincRNA:ch	-1.644246	10.334567	-2.411683	0.0234425	0.3724479	-3.384792
lincRNA:ch	-1.644834	15.431267	-2.278886	0.0313563	0.4049891	-3.626083
lincRNA:ch	-1.645343	8.9859	-2.402573	0.0239204	0.3755592	-3.401608
C21orf116	-1.645538	10.367467	-2.191455	0.0378205	0.4266764	-3.780248
DHFRL1	-1.645617	21.154933	-3.482797	0.0018185	0.1594252	-1.202204
FZD2	-1.64639	24.231842	-3.036637	0.0054782	0.228143	-2.152938

lincRNA:ch	-1.647359	36.598917	-2.288286	0.0307248	0.402581	-3.609282
lincRNA:ch	-1.648319	27.699233	-2.597827	0.0154131	0.319129	-3.033263
KIR2DL5A	-1.650473	16.807	-2.704514	0.0120524	0.2962939	-2.825424
METRN	-1.651726	30.463533	-2.339924	0.0274571	0.391968	-3.516215
ENST00000	-1.653841	7.9585333	-2.296534	0.0301801	0.4004419	-3.594503
ADRB2	-1.65469	34.560237	-2.86986	0.0081724	0.2581722	-2.495108
lincRNA:ch	-1.655427	15.744983	-2.204483	0.0367866	0.4228649	-3.757523
ENST00000	-1.656249	7.0418833	-2.264833	0.0323226	0.4084176	-3.651123
MED21	-1.657766	27.105225	-3.871805	0.0006757	0.1236385	-0.343843
TP53RK	-1.657965	16.086435	-2.605102	0.0151586	0.3175738	-3.019231
RP11-167P2	-1.658011	9.4001833	-2.081906	0.0476022	0.4592922	-3.967798
lincRNA:ch	-1.658187	26.178833	-3.627989	0.0012598	0.1467636	-0.884284
lincRNA:ch	-1.658364	22.972483	-3.644448	0.0012082	0.14461	-0.848038
lincRNA:ch	-1.659813	12.934717	-2.070624	0.048728	0.4612558	-3.986743
DRD2	-1.65992	8.6282583	-2.697546	0.012249	0.2966677	-2.839131
CHRNA4	-1.662897	12.350392	-2.990858	0.0061185	0.2368074	-2.247691
C5orf52	-1.66367	7.2975	-2.734061	0.0112512	0.2910568	-2.767103
LAX1	-1.664042	21.989917	-2.764235	0.0104848	0.2820898	-2.707219
FBXO30	-1.664708	27.605083	-2.420403	0.0229933	0.3695591	-3.36866
HPX-2	-1.666088	13.692408	-2.663861	0.0132427	0.3050874	-2.905136
A_33_P334	-1.667841	29.14975	-2.160971	0.0403427	0.4359062	-3.833079
lincRNA:ch	-1.667938	7.3957333	-2.143873	0.0418225	0.4421393	-3.862496
SPIB	-1.668966	28.639158	-2.484814	0.0199127	0.3495379	-3.248456
CRIP2	-1.669113	19.479133	-2.218136	0.0357306	0.4210607	-3.733614
STX1B	-1.669828	11.606292	-3.082522	0.0049009	0.2225409	-2.057374
LOC100132	-1.670014	17.7261	-2.534392	0.0178066	0.3355597	-3.154707
KLHL9	-1.67019	31.40935	-2.905879	0.007501	0.2537778	-2.421934
LOC100131	-1.670895	22.83715	-2.065355	0.0492618	0.4631831	-3.995568
OR2A1	-1.671287	11.521883	-2.114496	0.0444792	0.4490226	-3.912675
FANCL	-1.671881	23.670422	-3.152274	0.0041337	0.2130784	-1.911022
ZNF180	-1.672951	22.4784	-3.392858	0.0022786	0.1688885	-1.397325
A_33_P342	-1.675986	13.865483	-2.385246	0.0248543	0.3810375	-3.433488
DLL3	-1.676074	9.2033083	-2.537184	0.0176944	0.3355597	-3.149398
FTSJD1	-1.676143	24.016417	-2.835762	0.0088602	0.2648239	-2.563994
LOC285804	-1.676338	12.081183	-2.790349	0.0098616	0.2760175	-2.655133
RNF34	-1.676828	30.7111	-2.682639	0.0126798	0.3011401	-2.868393
BOK	-1.677778	20.570258	-2.95116	0.0067311	0.2412766	-2.329361
FHL2	-1.677983	21.036983	-2.532287	0.0178916	0.3363198	-3.158709
lincRNA:ch	-1.678571	23.798483	-2.279715	0.0313002	0.4049891	-3.624604
BX114012	-1.678747	12.514133	-2.243769	0.0338216	0.4150389	-3.688468
ZNF92	-1.679158	29.116383	-3.30003	0.0028714	0.1891324	-1.59705
A_33_P335	-1.68141	8.7584	-2.551362	0.0171345	0.3328324	-3.12238
DUX3	-1.681547	12.857017	-2.713364	0.011807	0.294918	-2.807989

A_33_P338(	-1.682448	14.004783	-2.136447	0.0424804	0.4431046	-3.875225
HSPH1	-1.6831	29.938806	-3.954096	0.0005467	0.1164353	-0.159996
LOC10013C	-1.684758	9.38735	-2.077819	0.0480073	0.4593556	-3.974669
ZNF831	-1.684973	30.922033	-3.559588	0.0014982	0.1515078	-1.034478
lincRNA:ch	-1.685404	10.5098	-2.208555	0.0364687	0.4224547	-3.750402
UNQ6494	-1.687108	8.46335	-2.241347	0.0339979	0.4150494	-3.692748
MGAT2	-1.68746	30.99705	-3.390472	0.0022923	0.1688885	-1.402481
RASL10B	-1.689379	9.7340833	-2.138403	0.0423062	0.4430089	-3.871875
lincRNA:ch	-1.690064	6.5727667	-2.453935	0.0213386	0.3594037	-3.306311
ADRA1D	-1.690241	10.822817	-2.691289	0.0124281	0.2990216	-2.851423
FAM90A10	-1.691357	16.313267	-2.898773	0.0076292	0.2548004	-2.436403
A_33_P331	-1.692414	11.525967	-2.341938	0.0273363	0.3917143	-3.512559
SUNC1	-1.692414	12.595567	-2.263514	0.0324147	0.4086958	-3.653468
FADD	-1.693207	35.731792	-2.952323	0.0067123	0.2412766	-2.326975
BSX	-1.693256	9.8719833	-2.114439	0.0444846	0.4490226	-3.912774
lincRNA:ch	-1.693354	15.065167	-2.445451	0.0217465	0.3614175	-3.322134
MORN4	-1.694039	9.64985	-2.18609	0.0382539	0.4281054	-3.789582
lincRNA:ch	-1.696115	6.7838167	-2.490779	0.0196477	0.3483849	-3.237233
IL26	-1.697133	9.6654833	-2.37442	0.0254546	0.3836446	-3.453336
C2orf42	-1.699052	22.512117	-3.852689	0.0007097	0.1271967	-0.386461
lincRNA:ch	-1.699443	11.29205	-2.865174	0.0082638	0.2587405	-2.504599
lincRNA:ch	-1.699776	16.537033	-2.816414	0.0092745	0.2693612	-2.602909
lincRNA:ch	-1.699952	37.771883	-2.252236	0.0332116	0.4120387	-3.673483
UFSP1	-1.70009	19.4775	-3.658271	0.0011665	0.1429549	-0.81757
PHLDA1	-1.701343	14.195767	-2.197149	0.0373654	0.4250149	-3.770327
lincRNA:ch	-1.703986	14.021117	-2.269261	0.0320153	0.4068732	-3.643244
lincRNA:ch	-1.704084	15.4203	-2.655458	0.013502	0.3058213	-2.921534
ENST0000C	-1.706316	9.8616	-2.500105	0.0192398	0.3461253	-3.219654
CBR1	-1.706943	28.125533	-2.6992	0.0122021	0.2963524	-2.835879
ENST0000C	-1.707295	6.4654333	-2.417984	0.0231171	0.3704211	-3.37314
LOC84856	-1.709194	10.14335	-2.270867	0.0319045	0.4060157	-3.640384
ZNF350	-1.709488	30.1105	-2.456734	0.0212056	0.359043	-3.301084
lincRNA:ch	-1.712269	5.7530667	-3.203618	0.0036441	0.2061571	-1.802504
lincRNA:ch	-1.714129	7.01155	-2.234977	0.0344657	0.4170539	-3.703991
POM121L9	-1.71591	6.8135667	-2.147848	0.0414743	0.4411319	-3.855671
lincRNA:ch	-1.717908	10.359067	-2.312449	0.029154	0.398859	-3.565894
ZNF284	-1.718103	15.996633	-3.240768	0.0033252	0.200491	-1.72359
SIT1	-1.719455	28.438083	-2.505518	0.0190066	0.345414	-3.209433
HIST1H4C	-1.719494	41.671117	-2.162592	0.0402049	0.4356864	-3.830282
CCDC29	-1.721491	12.686217	-3.430683	0.0020728	0.1646378	-1.315447
CRLF2	-1.721843	16.727783	-2.472659	0.0204632	0.3543941	-3.27128
lincRNA:ch	-1.722274	29.398483	-2.420291	0.022999	0.3695591	-3.368868
ENPP4	-1.723664	29.408167	-2.917493	0.0072959	0.2505573	-2.39825

PRAMEF22	-1.724585	7.15785	-2.603191	0.0152251	0.3175738	-3.02292
C9orf40	-1.725113	21.9282	-2.455053	0.0212854	0.359043	-3.304224
LOC100127	-1.727326	16.923783	-2.215792	0.0359099	0.4210607	-3.737725
lincRNA:ch	-1.731536	8.9338667	-2.46196	0.0209592	0.3574747	-3.291315
LOC220980	-1.736822	6.6469667	-2.46271	0.0209241	0.3574747	-3.289912
LOC730351	-1.740425	13.086033	-2.498651	0.0193028	0.3462109	-3.222396
GLYATL1	-1.740817	15.185567	-2.769141	0.010365	0.2806665	-2.697452
COL10A1	-1.741062	19.657925	-2.982757	0.0062391	0.237308	-2.264396
lincRNA:ch	-1.742814	18.823467	-2.346422	0.0270692	0.390281	-3.504413
lincRNA:ch	-1.745908	15.7479	-3.547102	0.0015463	0.1515078	-1.061816
A_33_P323	-1.747259	12.05575	-2.235227	0.0344472	0.4170539	-3.70355
OR2T33	-1.747513	9.9642667	-2.384955	0.0248702	0.3810375	-3.434021
GEMIN6	-1.74859	29.376083	-3.124839	0.0044206	0.2176909	-1.968738
lincRNA:ch	-1.74908	20.8684	-3.528232	0.0016217	0.1530728	-1.103084
lincRNA:ch	-1.750587	19.397583	-2.923588	0.0071904	0.2500476	-2.385804
ENST00000	-1.752389	11.599117	-2.142025	0.0419854	0.4421393	-3.865666
ENST00000	-1.752428	22.69295	-3.48137	0.001825	0.1594547	-1.205312
NAT12	-1.75282	19.009083	-2.98396	0.006221	0.237308	-2.261917
ENST00000	-1.755541	11.8237	-2.193333	0.0376698	0.4265606	-3.776977
C5orf58	-1.756109	17.842883	-3.262243	0.0031533	0.195744	-1.677828
lincRNA:ch	-1.758459	41.950883	-2.908514	0.007454	0.2524505	-2.416563
lincRNA:ch	-1.762199	13.114967	-2.910859	0.0074124	0.2524505	-2.411783
lincRNA:ch	-1.76351	11.752183	-2.331711	0.0279547	0.3936325	-3.531103
LOC100131	-1.763804	11.682533	-2.200129	0.0371292	0.4242006	-3.765127
LOC402382	-1.764039	10.048733	-2.290429	0.0305823	0.4015677	-3.605444
ZNF175	-1.764303	17.185408	-2.662041	0.0132984	0.3051802	-2.90869
HES2	-1.764724	9.7294167	-2.082792	0.0475148	0.4592922	-3.966307
lincRNA:ch	-1.764999	11.15625	-2.122905	0.0437038	0.4463484	-3.89836
DBT	-1.765508	25.075283	-3.209909	0.0035881	0.2061571	-1.789164
LINGO1	-1.766702	7.5348	-2.069694	0.0488219	0.4615296	-3.988302
A_33_P341	-1.767055	25.7047	-3.012952	0.0058011	0.2321647	-2.202036
TMEM60	-1.77009	28.720183	-3.142033	0.0042386	0.2140681	-1.93259
KCNJ9	-1.770628	6.5550917	-2.56063	0.0167775	0.3294068	-3.104675
ZC3H10	-1.770873	20.64125	-3.313228	0.0027789	0.1867656	-1.568764
SOCS4	-1.772008	29.003217	-3.174811	0.0039114	0.2093424	-1.863469
MGC44328	-1.773046	10.9816	-2.428885	0.0225639	0.3675764	-3.352937
PRMT6	-1.773072	24.213012	-3.493315	0.001771	0.1574654	-1.179291
LOC100287	-1.777961	18.367883	-2.203363	0.0368744	0.4230074	-3.759479
PLEKHF1	-1.780056	26.596967	-2.188961	0.0380214	0.4272702	-3.78459
lincRNA:ch	-1.781211	14.49245	-2.189023	0.0380163	0.4272702	-3.784481
DENND2D	-1.782014	35.689033	-4.712447	7.61E-05	0.064665	1.548867
ENST00000	-1.783071	10.332933	-2.224558	0.0352433	0.419515	-3.722334
C2orf88	-1.783189	23.597233	-2.154506	0.0408966	0.4392426	-3.84422

A_33_P331	-1.78828	13.981567	-2.38604	0.0248107	0.3810375	-3.432029
lincRNA:ch	-1.788515	23.620567	-2.528873	0.0180304	0.3376943	-3.165195
lincRNA:ch	-1.789043	10.283117	-2.333934	0.0278192	0.3931293	-3.527077
A_33_P326.	-1.789866	10.335617	-2.58751	0.0157808	0.3214	-3.053126
CDH16	-1.791187	8.0355917	-2.306065	0.0295617	0.399347	-3.577384
ENST0000C	-1.79155	20.23525	-2.359442	0.026307	0.3875594	-3.480706
lincRNA:ch	-1.79574	14.338217	-2.28876	0.0306932	0.4023615	-3.608433
SDR42E1	-1.796445	14.457217	-2.827485	0.0090352	0.2666534	-2.580657
C1orf63	-1.801399	30.509733	-2.550383	0.0171726	0.3329917	-3.124249
CMKLR1	-1.801403	20.265846	-3.303979	0.0028434	0.1882099	-1.588591
SHC3	-1.802149	7.4688056	-2.121475	0.0438348	0.4469511	-3.900797
ZNF259P	-1.802887	17.857	-2.140892	0.0420855	0.442698	-3.86761
lincRNA:ch	-1.804375	7.0606667	-2.17211	0.0394041	0.4325574	-3.813831
THAP6	-1.805785	25.703067	-2.771785	0.0103009	0.2801815	-2.692184
ESX1	-1.806098	11.616733	-2.945587	0.0068216	0.2432702	-2.340789
LOC441493	-1.806568	7.6141333	-2.634725	0.0141625	0.3109118	-2.961879
SERPINI2	-1.806822	12.55905	-2.135759	0.0425418	0.4431575	-3.876403
GIMAP8	-1.807566	29.833883	-2.346797	0.027047	0.390281	-3.503732
lincRNA:ch	-1.807841	15.841117	-2.908805	0.0074488	0.2524505	-2.415971
LOC100129	-1.808761	7.756	-2.58362	0.0159215	0.3231011	-3.060604
lincRNA:ch	-1.80974	14.101033	-3.100982	0.0046855	0.2225409	-2.018766
lincRNA:ch	-1.811913	24.542583	-2.824044	0.009109	0.2679712	-2.587578
LOC100129	-1.813578	14.2499	-3.60114	0.0013486	0.1482829	-0.943323
ENST0000C	-1.81489	7.3487167	-2.357965	0.0263925	0.3875594	-3.4834
LOC729426	-1.81534	6.5352	-2.744019	0.0109926	0.2873459	-2.747376
LOC100128	-1.815673	28.650183	-2.351122	0.0267917	0.3890091	-3.495864
PID1	-1.815849	28.201833	-2.398135	0.0241565	0.3765593	-3.409787
UBFD1	-1.819197	23.463183	-3.890914	0.0006433	0.1208764	-0.301206
lincRNA:ch	-1.819236	18.454217	-4.751037	6.88E-05	0.064665	1.6358633
BE468260	-1.820078	16.173033	-2.64515	0.0138267	0.3083784	-2.941613
LOC100131	-1.822389	12.5104	-2.260638	0.0326163	0.4098389	-3.658578
TRIM13	-1.824846	28.819642	-2.983394	0.0062295	0.237308	-2.263082
KIAA1279	-1.827343	20.059317	-2.846467	0.0086386	0.2625673	-2.542408
KIR2DS2	-1.82886	25.834258	-2.454743	0.0213001	0.3591018	-3.304803
A_33_P329	-1.833197	19.1856	-4.517606	0.0001266	0.0677403	1.1090995
DOCK4	-1.833716	24.555592	-2.293344	0.0303897	0.4011176	-3.600223
TPTE2	-1.838053	6.7041333	-2.343959	0.0272156	0.3907322	-3.508889
ENST0000C	-1.838229	9.8761833	-2.293042	0.0304096	0.4011176	-3.600763
A_24_P392	-1.838817	20.421683	-2.202488	0.0369433	0.4230074	-3.761009
UNC5A	-1.841108	17.208333	-2.613005	0.0148867	0.3170467	-3.003964
FOXA3	-1.841636	15.607083	-3.091121	0.0047994	0.2225409	-2.039401
AQP7	-1.844975	13.484275	-2.720866	0.0116026	0.2935077	-2.793186
lincRNA:ch	-1.845846	11.759767	-2.69063	0.0124471	0.2990216	-2.852716

lincRNA:ch	-1.850526	24.10065	-3.048692	0.0053205	0.2259383	-2.127888
lincRNA:ch	-1.851838	26.548667	-2.956998	0.0066375	0.2412766	-2.317382
LOC100131	-1.851857	9.1341833	-2.582108	0.0159766	0.3231011	-3.06351
C5orf54	-1.854775	14.061367	-2.3614	0.0261941	0.3875594	-3.477135
VIT	-1.855832	17.972267	-2.400107	0.0240514	0.3757321	-3.406154
XCL1	-1.85642	29.199567	-2.665713	0.0131861	0.3044196	-2.901518
lincRNA:ch	-1.857653	9.0091167	-2.68122	0.0127215	0.3016318	-2.871175
KRTAP20-4	-1.858985	12.34625	-2.535375	0.017767	0.3355597	-3.152838
lincRNA:ch	-1.859513	11.5556	-2.161162	0.0403264	0.4359062	-3.832749
lincRNA:ch	-1.860864	14.28105	-2.234912	0.0344705	0.4170539	-3.704104
lincRNA:ch	-1.862724	15.979133	-3.447865	0.0019853	0.1624754	-1.278167
PPP1R14D	-1.866758	13.679167	-2.235868	0.0343999	0.4170539	-3.702419
lincRNA:ch	-1.868873	11.415367	-2.625073	0.0144801	0.3139876	-2.980604
ZNF735	-1.870008	13.734933	-2.724923	0.0114935	0.2935077	-2.785173
LOC643072	-1.870694	17.604417	-2.37764	0.0252746	0.3829881	-3.447437
OPN5	-1.871389	6.8894583	-3.070119	0.0050509	0.2240985	-2.083262
DEFB103A	-1.872867	7.5527667	-2.207618	0.0365417	0.4224608	-3.752042
A_33_P333	-1.876548	11.3687	-2.672703	0.0129748	0.3029433	-2.887851
ENST0000C	-1.877821	11.232083	-2.312759	0.0291343	0.398859	-3.565334
LOC100131	-1.877938	7.9483833	-2.126841	0.043345	0.445974	-3.891646
LOC221814	-1.879544	16.59035	-2.536391	0.0177262	0.3355597	-3.150906
C1orf25	-1.879564	22.463467	-2.570335	0.0164111	0.3270385	-3.086098
RP11-526D	-1.882501	10.743367	-2.125331	0.0434824	0.4460421	-3.894223
lincRNA:ch	-1.883656	22.58725	-2.312351	0.0291602	0.398859	-3.566069
ENST0000C	-1.883813	21.638983	-2.704841	0.0120433	0.2962939	-2.824779
ENST0000C	-1.88442	29.1788	-2.223413	0.0353298	0.4195518	-3.724347
SLC41A2	-1.884576	10.327333	-2.908483	0.0074545	0.2524505	-2.416628
C2orf44	-1.88487	20.396483	-3.017033	0.0057442	0.2312816	-2.193587
lincRNA:ch	-1.888375	10.487167	-2.202665	0.0369293	0.4230074	-3.760699
ZNF79	-1.889138	21.205917	-4.158771	0.000322	0.0995993	0.2994859
ELOVL2	-1.889158	8.3274333	-2.064652	0.0493334	0.4632547	-3.996744
SLC5A3	-1.889236	28.0567	-3.865884	0.0006861	0.1242328	-0.357048
TRPC1	-1.889432	16.999267	-2.958081	0.0066202	0.2412766	-2.315156
lincRNA:ch	-1.891234	22.5554	-2.419914	0.0230182	0.3696985	-3.369566
TBC1D30	-1.891762	6.83795	-2.061716	0.0496336	0.4634338	-4.001654
HSPA1B	-1.892947	29.772108	-2.327016	0.0282427	0.3959544	-3.539598
C16orf88	-1.89515	23.138733	-2.337777	0.0275864	0.3921472	-3.52011
ZNF431	-1.900945	6.0820667	-2.254806	0.0330285	0.4111299	-3.668927
C9orf96	-1.90118	13.352267	-2.809333	0.0094306	0.2699357	-2.617121
ZNF649	-1.901435	15.343183	-2.448382	0.0216048	0.3614175	-3.316672
ENST0000C	-1.902375	7.3715833	-2.344812	0.0271648	0.3906966	-3.507339
SLC35D3	-1.902845	18.588383	-2.23351	0.0345743	0.4173873	-3.706577
lincRNA:ch	-1.902884	18.871417	-2.243197	0.0338632	0.4150494	-3.689479

lincRNA:ch	-1.903648	25.591767	-4.581905	0.000107	0.0671784	1.2542939
lincRNA:ch	-1.905841	13.147633	-2.308534	0.0294034	0.399347	-3.572942
PTCRA	-1.907877	33.602567	-2.230749	0.0347794	0.4181246	-3.711441
TMSB4X	-1.909639	42.623467	-2.233681	0.0345616	0.4173873	-3.706275
ENST0000C	-1.910109	13.408267	-2.455032	0.0212864	0.359043	-3.304262
LOC440896	-1.910129	16.318983	-3.821777	0.0007683	0.1297134	-0.455296
ACOT12	-1.91148	13.606833	-2.23759	0.0342732	0.4164646	-3.699382
ZNF239	-1.913301	18.874683	-2.900001	0.0076069	0.2548004	-2.433903
MT1B	-1.913555	33.6308	-2.14206	0.0419823	0.4421393	-3.865607
PATE2	-1.913888	14.132183	-2.253116	0.0331488	0.4115516	-3.671923
lincRNA:ch	-1.915944	6.5942333	-2.111638	0.0447456	0.4501553	-3.917533
LOC100127	-1.916845	10.1528	-2.3202	0.0286658	0.3971357	-3.551914
A_33_P324	-1.916982	6.2350167	-3.576806	0.0014344	0.1491954	-0.996739
LOC100132	-1.919331	14.310217	-2.32512	0.0283598	0.3967766	-3.543027
lincRNA:ch	-1.920213	24.408067	-3.110285	0.0045805	0.2214141	-1.999276
ENST0000C	-1.924971	7.8450167	-2.50147	0.0191807	0.3457445	-3.217078
C21orf15	-1.926498	13.204917	-2.801662	0.0096026	0.2730684	-2.632496
MAMSTR	-1.927898	20.099158	-3.170929	0.0039489	0.2097239	-1.871671
LOC387647	-1.928084	20.865367	-3.294988	0.0029076	0.1891663	-1.607847
RNF186	-1.928319	9.7003667	-2.215904	0.0359013	0.4210607	-3.737528
C14orf115	-1.928456	7.2497833	-2.435022	0.0222579	0.365722	-3.34154
PHYHIPL	-1.928613	9.4767167	-2.651638	0.0136215	0.3069363	-2.928981
lincRNA:ch	-1.929102	10.060633	-2.878767	0.0080013	0.2571214	-2.477053
ENST0000C	-1.92967	12.761817	-2.649241	0.013697	0.3072994	-2.93365
TTTY22	-1.930159	9.3305333	-2.788748	0.0098988	0.2760175	-2.658333
LOC100133	-1.930218	13.812283	-3.31201	0.0027873	0.1869719	-1.571375
IGLON5	-1.931824	19.20345	-2.422277	0.0228978	0.3689503	-3.36519
LOC100127	-1.93198	7.0999833	-2.620178	0.0146438	0.3149228	-2.990086
ENST0000C	-1.932333	8.7764833	-2.063276	0.0494739	0.463389	-3.999045
LOC100130	-1.932764	22.10985	-4.087039	0.0003878	0.107147	0.1381525
KIR2DL2	-1.93481	19.368942	-2.394346	0.0243597	0.3782092	-3.416763
lincRNA:ch	-1.935387	20.095483	-3.322825	0.0027134	0.1841342	-1.548173
FAM50B	-1.935564	25.185533	-3.827468	0.0007572	0.1288463	-0.442631
JAKMIP3	-1.935583	15.61805	-2.215244	0.0359519	0.4210607	-3.738685
CETN3	-1.93992	23.019792	-3.24634	0.0032797	0.2000399	-1.711727
lincRNA:ch	-1.942417	17.061567	-2.629843	0.0143223	0.3122587	-2.971354
MT1E	-1.943895	31.449075	-2.202422	0.0369484	0.4230074	-3.761123
A_33_P332	-1.945354	15.424267	-3.078729	0.0049464	0.2225409	-2.065297
lincRNA:ch	-1.947136	18.689883	-2.830391	0.0089734	0.2659145	-2.574809
A_33_P325	-1.954048	6.6448667	-3.445719	0.001996	0.1624754	-1.282826
A_24_P419	-1.956358	8.4982333	-2.396592	0.0242391	0.3770692	-3.412628
A2BP1	-1.957337	12.435267	-2.695421	0.0123096	0.2977217	-2.843307
A_33_P331	-1.958003	24.151633	-2.451355	0.0214619	0.3602653	-3.311126

LOC284108	-1.961508	10.988717	-2.433376	0.0223396	0.3659732	-3.344599
lincRNA:ch	-1.965443	40.385567	-2.096487	0.0461816	0.4551037	-3.943207
LOC284440	-1.967421	10.66835	-2.080761	0.0477154	0.459347	-3.969724
LOC100288	-1.967499	6.4548167	-2.295289	0.0302617	0.4005965	-3.596735
lincRNA:ch	-1.96934	18.080183	-3.82664	0.0007588	0.1288463	-0.444474
ZNF100	-1.969487	19.218558	-2.956452	0.0066462	0.2412766	-2.318502
SH2D2A	-1.970779	33.455858	-3.910902	0.0006111	0.1199786	-0.256572
TMEM114	-1.973138	8.9104167	-2.667554	0.0131301	0.3040014	-2.897921
LOC643441	-1.981499	6.0132333	-2.593779	0.0155564	0.3200006	-3.041062
APOL5	-1.981695	7.1176	-2.546439	0.017327	0.3342535	-3.13177
LOC146795	-1.982048	20.4477	-2.124191	0.0435863	0.4462653	-3.896167
LOC729678	-1.985102	29.6247	-3.555918	0.0015122	0.1515078	-1.042516
EPHB3	-1.985435	16.350483	-2.860492	0.0083562	0.260164	-2.514073
ICAM4	-1.986443	26.768992	-2.52435	0.0182157	0.3388496	-3.173779
hCG_18144	-1.989214	8.2068	-2.22273	0.0353814	0.4200225	-3.725548
ENST00000	-1.989938	17.124917	-2.871872	0.0081335	0.2579482	-2.491032
lincRNA:ch	-1.990643	14.852717	-4.291066	0.0002284	0.0888702	0.5976347
lincRNA:ch	-1.991662	17.121183	-2.168464	0.0397091	0.4342284	-3.820139
ENST00000	-1.992543	7.1626333	-2.712891	0.01182	0.294918	-2.80892
GBP3	-1.992856	28.7945	-3.242894	0.0033078	0.200491	-1.719065
SERPINA1	-1.997545	8.5791417	-2.580601	0.0160316	0.3234917	-3.066405
A_33_P323	-1.99781	7.3578167	-2.643904	0.0138664	0.3088707	-2.944038
GNG11	-1.997827	36.886862	-2.143541	0.0418518	0.4421393	-3.863066
lincRNA:ch	-2.000434	12.31265	-3.657143	0.0011699	0.1429549	-0.820057
A_33_P340	-2.001334	8.4428167	-2.487729	0.0197828	0.3487739	-3.242974
BCDIN3D	-2.002098	27.698767	-3.93574	0.0005732	0.116472	-0.201056
lincRNA:ch	-2.008109	25.025583	-2.473701	0.0204154	0.3540935	-3.269325
ATP6V0A4	-2.011252	7.1054083	-4.101147	0.0003739	0.1057281	0.1698622
lincRNA:ch	-2.015961	18.040167	-3.797969	0.0008166	0.1315436	-0.508246
MEG8	-2.016959	15.429517	-2.420295	0.0229988	0.3695591	-3.368861
MYOZ3	-2.016979	18.382233	-2.400864	0.0240111	0.3756903	-3.404758
lincRNA:ch	-2.017703	8.82875	-2.841856	0.0087334	0.2636111	-2.551712
CFLP1	-2.020406	8.71885	-2.645337	0.0138207	0.3083784	-2.94125
lincRNA:ch	-2.021463	13.52155	-2.992844	0.0060893	0.2367259	-2.243593
HIST1H4L	-2.021894	24.375517	-2.328437	0.0281552	0.3953385	-3.537028
ZNF433	-2.023049	14.5572	-2.123024	0.043693	0.4463484	-3.898158
PCDHA12	-2.02346	8.70625	-2.590736	0.0156649	0.3202106	-3.04692
A_33_P322	-2.026143	6.3912333	-2.239075	0.0341641	0.4160284	-3.69676
EBF2	-2.031978	11.4436	-2.381239	0.0250749	0.3823116	-3.44084
ENST00000	-2.033035	8.0815	-2.26647	0.0322086	0.4080885	-3.64821
ZNF613	-2.033427	24.335033	-3.733046	0.0009641	0.1367135	-0.652303
BBS10	-2.033681	23.64635	-2.620544	0.0146315	0.3149228	-2.989377
lincRNA:ch	-2.034014	14.345333	-3.432923	0.0020611	0.1644633	-1.31059

LOC100131	-2.037303	16.126133	-2.7846	0.0099957	0.276697	-2.666621
lincRNA:ch	-2.040201	11.6746	-2.606676	0.0151041	0.3175738	-3.016192
lincRNA:ch	-2.040652	14.097883	-2.431603	0.0224279	0.3664461	-3.347891
C6orf170	-2.043324	10.967308	-2.436816	0.0221691	0.3646635	-3.338206
PIH1D2	-2.045038	20.886017	-2.461182	0.0209957	0.3575926	-3.29277
KLKBL4	-2.046976	6.9129667	-2.236977	0.0343182	0.4167431	-3.700462
PARS2	-2.049267	20.071217	-3.191093	0.0037581	0.2065576	-1.829036
lincRNA:ch	-2.049659	11.82195	-2.828335	0.0090171	0.2663904	-2.578948
lincRNA:ch	-2.049992	8.7509333	-2.207216	0.036573	0.4224608	-3.752744
lincRNA:ch	-2.058724	13.282967	-3.195964	0.0037133	0.2061571	-1.818723
ZNF564	-2.058881	24.4727	-3.687682	0.0010824	0.1399536	-0.752653
QSER1	-2.060643	25.48	-2.933163	0.0070276	0.2472862	-2.366231
A_33_P328(	-2.062092	10.920233	-2.767709	0.0103999	0.2811016	-2.700305
PLA2G2E	-2.062131	6.0512667	-2.344647	0.0271746	0.3906966	-3.507638
ZNF675	-2.064207	26.537233	-2.719608	0.0116366	0.2935077	-2.795671
TNC	-2.066566	13.746192	-3.144216	0.004216	0.2139793	-1.927994
MS4A7	-2.067085	34.294983	-3.50889	0.0017028	0.1541747	-1.145323
lincRNA:ch	-2.067692	13.062	-4.50463	0.0001309	0.0677403	1.0797951
C5orf53	-2.068476	20.595867	-2.388742	0.0246632	0.3803859	-3.427067
LOC100289	-2.069494	6.7195333	-2.372923	0.0255386	0.3843614	-3.456075
A_33_P340'	-2.069729	28.331333	-2.694187	0.0123449	0.2979568	-2.845732
ZNF19	-2.071726	18.604833	-4.267474	0.0002428	0.0890075	0.5444222
TMEM140	-2.074252	29.849283	-2.370651	0.0256666	0.3848233	-3.460232
hCG_2045C	-2.074467	5.9455667	-2.38581	0.0248233	0.3810375	-3.432453
ENST0000C	-2.080008	18.260783	-2.409306	0.0235663	0.372736	-3.389184
lincRNA:ch	-2.08181	7.7715167	-2.091656	0.046648	0.455826	-3.951368
OR6P1	-2.082338	11.640067	-3.030612	0.0055587	0.2292946	-2.165443
AK125749	-2.086	9.8096833	-2.497646	0.0193465	0.3465617	-3.224292
C1orf173	-2.087782	7.3661	-2.132719	0.0428141	0.4448582	-3.881604
lincRNA:ch	-2.089055	16.175483	-3.67036	0.0011312	0.1421743	-0.790902
lincRNA:ch	-2.08972	12.56745	-2.968133	0.0064624	0.2397592	-2.2945
lincRNA:ch	-2.098297	9.44615	-2.996694	0.0060331	0.23565	-2.235645
A_24_P335.	-2.100685	42.743983	-3.896678	0.0006338	0.1203925	-0.288339
RP3-398D1	-2.101116	8.21695	-2.705847	0.0120151	0.2962939	-2.822798
HIST1H4B	-2.101547	33.128317	-2.598522	0.0153886	0.319129	-3.031924
PRR5L	-2.102234	24.628112	-3.490352	0.0017842	0.1574654	-1.185747
lincRNA:ch	-2.104562	17.897483	-3.083078	0.0048943	0.2225409	-2.056213
PANK1	-2.105052	14.8722	-2.858914	0.0083875	0.2602147	-2.517265
ENST0000C	-2.110887	18.339767	-2.875289	0.0080677	0.2574548	-2.484106
hCG_16457	-2.113413	26.817817	-4.443945	0.0001534	0.0724439	0.9427429
OR5H2	-2.114255	6.3198333	-2.924043	0.0071826	0.2500247	-2.384875
A_33_P336.	-2.115371	25.989483	-2.105935	0.0452814	0.4518753	-3.927212
lincRNA:ch	-2.117368	18.724183	-3.600011	0.0013525	0.1482829	-0.945803

FAM81A	-2.125964	9.366	-2.286029	0.0308753	0.4033224	-3.61332
lincRNA:ch	-2.126179	9.7214833	-2.963977	0.0065272	0.2411412	-2.303046
THAP2	-2.127667	16.27395	-2.727815	0.0114163	0.2932362	-2.779458
AWAT1	-2.128803	7.8275167	-2.869326	0.0081828	0.2581722	-2.496191
KCNE3	-2.130467	32.001433	-2.62171	0.0145924	0.3149228	-2.987119
lincRNA:ch	-2.13129	8.1629333	-2.27734	0.0314613	0.4050872	-3.628842
lincRNA:ch	-2.131838	11.9294	-3.579632	0.0014241	0.1491954	-0.99054
ENST0000C	-2.132641	8.0267833	-2.399203	0.0240995	0.3760059	-3.407819
LOC645978	-2.132738	11.553967	-2.412763	0.0233864	0.3722428	-3.382797
CCDC124	-2.133306	24.08315	-2.886252	0.0078601	0.2567402	-2.46186
lincRNA:ch	-2.140708	11.81005	-2.362737	0.0261173	0.3873866	-3.474693
SPRR4	-2.144193	7.9080167	-2.360273	0.0262591	0.3875594	-3.479191
lincRNA:ch	-2.145074	17.087467	-3.482439	0.0018201	0.1594252	-1.202985
ENST0000C	-2.146562	8.5955333	-2.702232	0.0121165	0.2962939	-2.829914
LOC10013C	-2.146954	19.699867	-2.219701	0.0356113	0.4210607	-3.730868
ENST0000C	-2.149323	15.602183	-2.566359	0.0165603	0.3279122	-3.093713
lincRNA:ch	-2.149617	9.6861333	-3.098643	0.0047123	0.2225409	-2.023664
A_33_P338	-2.151712	7.0316167	-2.427947	0.022611	0.3676756	-3.354677
LOC728317	-2.155628	11.08555	-2.264699	0.0323319	0.4084176	-3.65136
KIR3DL3	-2.156999	16.476717	-3.081066	0.0049183	0.2225409	-2.060414
LOC100129	-2.160699	12.602567	-3.69225	0.0010698	0.1399536	-0.742561
A_32_P556	-2.160738	8.0444	-3.031912	0.0055412	0.2292353	-2.162746
A_33_P322	-2.16162	6.95065	-2.479048	0.0201721	0.3519698	-3.259291
HILS1	-2.16209	9.73945	-3.02692	0.0056085	0.2300777	-2.1731
lincRNA:ch	-2.163421	10.539783	-2.857339	0.0084189	0.2602147	-2.520449
A_33_P340	-2.165712	12.022033	-2.766196	0.0104368	0.2818819	-2.703316
lincRNA:ch	-2.170646	21.330633	-2.979168	0.0062932	0.2375467	-2.271789
THAP10	-2.177985	9.36656	-3.077567	0.0049604	0.2225409	-2.067723
SNAR-B2	-2.17889	7.15995	-2.111346	0.0447729	0.4501553	-3.918029
ANKRD56	-2.179379	7.9482667	-2.11272	0.0446446	0.4499092	-3.915695
LOC10013C	-2.181083	6.4926167	-3.206713	0.0036164	0.2061571	-1.795942
C17orf64	-2.183334	8.0894333	-3.147219	0.0041851	0.2139793	-1.921671
LOC651868	-2.186115	11.4968	-2.129456	0.0431081	0.4454091	-3.88718
LOC100131	-2.186134	11.246317	-2.769637	0.010353	0.2806665	-2.696465
lincRNA:ch	-2.19612	7.1954167	-2.378665	0.0252176	0.3829079	-3.445558
C6orf203	-2.196492	27.067833	-3.764218	0.0008903	0.1345828	-0.583197
MMAA	-2.196571	19.6945	-3.608319	0.0013243	0.1478675	-0.927547
lincRNA:ch	-2.196708	13.204917	-2.242135	0.0339404	0.4150494	-3.691355
ZNF69	-2.198333	11.0432	-2.77997	0.010105	0.2776412	-2.675863
lincRNA:ch	-2.199958	18.530283	-2.122973	0.0436976	0.4463484	-3.898244
A_33_P338	-2.205832	7.6480833	-2.266581	0.032201	0.4080885	-3.648014
LOC100131	-2.20685	18.23255	-2.255699	0.032965	0.4111299	-3.667343
LOC729986	-2.209278	6.8006167	-3.021525	0.0056822	0.2306829	-2.184284

lincRNA:ch	-2.211315	21.67235	-2.205754	0.0366872	0.4227429	-3.755302
SH2D1B	-2.211334	29.133067	-2.314609	0.0290172	0.398859	-3.562001
A_33_P323	-2.214663	11.1769	-3.223581	0.0034692	0.2045187	-1.76014
lincRNA:ch	-2.214976	20.936767	-3.563271	0.0014843	0.1515078	-1.026409
LOC643008	-2.225119	22.2684	-3.292536	0.0029253	0.1893274	-1.613094
lincRNA:ch	-2.225765	26.52965	-2.839939	0.0087731	0.2637443	-2.555577
OR1K1	-2.229172	15.63835	-2.598317	0.0153958	0.319129	-3.032318
lincRNA:ch	-2.229564	8.1926833	-2.514117	0.0186416	0.3419981	-3.193173
lincRNA:ch	-2.231776	7.0256667	-2.13037	0.0430255	0.4452977	-3.885619
ZNF616	-2.232971	20.745783	-4.035747	0.0004428	0.1111414	0.0229753
CDRT15L1	-2.233284	9.57285	-2.916942	0.0073055	0.2505573	-2.399376
LOC100133	-2.233832	13.487717	-2.134947	0.0426143	0.4436477	-3.877791
DKFZp547	-2.235712	8.1733167	-2.109697	0.0449273	0.4506787	-3.920829
LOC100130	-2.240078	6.9407333	-2.306607	0.0295269	0.399347	-3.57641
AIM2	-2.241008	29.628375	-2.181349	0.0386404	0.4304893	-3.797816
lincRNA:ch	-2.241821	9.6321167	-3.031817	0.0055425	0.2292353	-2.162943
lincRNA:ch	-2.24513	9.4936333	-2.54649	0.017325	0.3342535	-3.131674
DCDC1	-2.245404	9.6268667	-2.878597	0.0080045	0.2571214	-2.477397
A_33_P335	-2.245815	6.7887167	-2.28292	0.0310838	0.4044488	-3.618879
lincRNA:ch	-2.247401	17.078367	-3.079298	0.0049395	0.2225409	-2.064107
lincRNA:ch	-2.249673	7.4263	-2.61994	0.0146518	0.3149228	-2.990547
A_33_P334	-2.251298	6.3705833	-3.516263	0.0016714	0.1530728	-1.12923
ENST00000	-2.254548	19.45195	-2.781146	0.0100771	0.2773488	-2.673516
ENST00000	-2.258406	10.499533	-2.426672	0.0226752	0.3678645	-3.357042
AADACL4	-2.261676	7.1436167	-2.095121	0.046313	0.4551037	-3.945515
lincRNA:ch	-2.262322	14.571667	-2.841725	0.0087361	0.2636111	-2.551975
A_33_P328	-2.265885	14.2233	-2.30223	0.0298091	0.399347	-3.584278
PTGDS	-2.267413	33.8142	-2.248845	0.0334547	0.4131477	-3.679488
ZNF91	-2.267432	20.298717	-3.590708	0.0013847	0.1487994	-0.966233
LCMT2	-2.269711	21.532537	-3.835329	0.000742	0.1288463	-0.42513
LOC647309	-2.274638	9.42445	-2.270999	0.0318953	0.4060157	-3.640148
ANKRD46	-2.279337	22.30445	-3.836092	0.0007406	0.1288463	-0.423431
WNT9A	-2.282411	8.0525667	-2.650127	0.013669	0.3072165	-2.931923
lincRNA:ch	-2.282959	8.1706333	-2.703378	0.0120843	0.2962939	-2.827659
lincRNA:ch	-2.290987	11.852867	-2.664072	0.0132362	0.3050874	-2.904724
ENST00000	-2.291379	8.0556	-2.367375	0.0258523	0.3856219	-3.466222
ADAM20	-2.292985	8.7427667	-2.32047	0.0286489	0.3971357	-3.551427
lincRNA:ch	-2.293239	16.636083	-4.865445	5.11E-05	0.064665	1.8934573
ENST00000	-2.295373	10.339	-3.355701	0.0025001	0.1751027	-1.477482
LOC100130	-2.296411	16.862183	-2.665666	0.0131875	0.3044196	-2.901609
lincRNA:ch	-2.29649	7.53305	-2.502649	0.0191299	0.3456829	-3.214852
THNSL1	-2.297136	16.0055	-3.223466	0.0034702	0.2045187	-1.760383
LOC100130	-2.298917	10.131917	-2.280189	0.0312681	0.4049891	-3.623757

RLN1	-2.300386	13.021867	-3.189724	0.0037707	0.2065576	-1.831933
TFAP2D	-2.305497	6.5227167	-3.614963	0.0013022	0.1469235	-0.91294
lincRNA:ch	-2.310313	15.136217	-2.243095	0.0338706	0.4150494	-3.68966
PEX12	-2.311116	21.3584	-3.320482	0.0027292	0.1848508	-1.553201
ENST0000C	-2.311116	10.304	-2.482532	0.020015	0.3507776	-3.252746
A_33_P334	-2.311997	9.29985	-2.298193	0.0300716	0.4000235	-3.591527
lincRNA:ch	-2.313309	13.808667	-2.838798	0.0087968	0.2638971	-2.557876
ENST0000C	-2.320965	13.819283	-2.127161	0.043316	0.445974	-3.8911
A_33_P334	-2.32167	7.4254833	-2.367534	0.0258433	0.3856219	-3.465931
ENST0000C	-2.328131	8.4271833	-3.142793	0.0042307	0.2139793	-1.930989
BQ879989	-2.329717	20.777633	-3.137832	0.0042824	0.215105	-1.941427
ZNF683	-2.346909	32.169667	-2.738199	0.0111431	0.2889307	-2.758911
LOC10013C	-2.347418	19.5055	-2.153675	0.0409683	0.4392951	-3.845651
DGKK	-2.347457	8.0481333	-3.1491	0.0041659	0.2139793	-1.91771
ZNF816A	-2.351354	25.10235	-3.758532	0.0009033	0.1345828	-0.595811
NP450512	-2.35245	17.081283	-3.609203	0.0013213	0.1478675	-0.925604
A_33_P325	-2.353175	15.8942	-2.870758	0.008155	0.2581722	-2.493291
LOC152225	-2.356934	19.2654	-2.230582	0.0347918	0.4181246	-3.711734
LOC100129	-2.358227	7.9975	-2.518168	0.0184719	0.3403137	-3.1855
PRG1	-2.358364	7.2017167	-2.377797	0.0252659	0.3829881	-3.44715
C10orf114	-2.362534	14.230767	-2.858035	0.008405	0.2602147	-2.519042
lincRNA:ch	-2.362769	10.432567	-2.263269	0.0324318	0.4086958	-3.653903
A_33_P339	-2.364336	12.7519	-2.771423	0.0103097	0.2801815	-2.692907
GPR18	-2.365354	27.561567	-3.586391	0.0014	0.1491954	-0.975708
A_33_P336	-2.365687	24.93855	-2.341294	0.0273749	0.3917302	-3.513727
lincRNA:ch	-2.366509	13.91705	-2.125592	0.0434586	0.4460421	-3.893777
A_33_P328	-2.370406	9.0528667	-3.226508	0.0034443	0.2045187	-1.753921
lincRNA:ch	-2.374126	17.377033	-2.6582	0.0134169	0.3055039	-2.916185
SENP8	-2.374312	12.193242	-3.049469	0.0053105	0.2257876	-2.126272
lincRNA:ch	-2.375497	7.4116	-2.299575	0.0299815	0.3995686	-3.589046
TMPRSS11	-2.378492	7.85365	-2.163249	0.0401491	0.4356864	-3.829147
lincRNA:ch	-2.39214	13.883567	-3.673989	0.0011208	0.1414069	-0.782892
FREM2	-2.39261	9.6079667	-2.653463	0.0135643	0.3064408	-2.925423
A_33_P325	-2.397759	10.48565	-2.699223	0.0122014	0.2963524	-2.835832
lincRNA:ch	-2.39913	20.554217	-3.59804	0.0013593	0.1482829	-0.950132
CD226	-2.404201	23.110033	-3.777776	0.0008599	0.1329829	-0.553104
ENST0000C	-2.40522	12.1751	-2.062571	0.0495459	0.4634338	-4.000223
A_33_P324	-2.406355	36.407467	-3.380657	0.0023492	0.1705453	-1.423676
FLJ43585	-2.406982	7.5348	-2.782224	0.0100517	0.2772659	-2.671365
ENST0000C	-2.40755	8.5027833	-2.874326	0.0080862	0.2575354	-2.486058
lincRNA:ch	-2.411192	30.594083	-2.970212	0.0064302	0.2393269	-2.290226
lincRNA:ch	-2.412778	15.708933	-2.217624	0.0357697	0.4210607	-3.734512
CDKN1C	-2.414941	36.060325	-2.594526	0.0155299	0.3196428	-3.039624

LOC284805	-2.418495	7.4522	-2.574445	0.0162582	0.3259932	-3.078219
NAPEPLD	-2.418985	25.368117	-4.181316	0.0003037	0.0992932	0.350246
TPPP3	-2.421941	22.536733	-2.996121	0.0060414	0.23565	-2.236828
AKR1C3	-2.429724	27.721808	-3.082512	0.0049011	0.2225409	-2.057395
A_33_P322	-2.434473	13.0578	-2.245912	0.0336662	0.4142947	-3.684678
LOC100132	-2.437841	9.4742667	-2.099761	0.0458679	0.4545041	-3.93767
UBE2DNL	-2.445594	21.302867	-3.124152	0.004428	0.2176909	-1.97018
ENST0000C	-2.446201	7.4086833	-2.772511	0.0102834	0.2801815	-2.690737
LOC100128	-2.447278	7.8309	-2.172654	0.0393587	0.432426	-3.812889
FLJ41455	-2.451899	14.108033	-2.208112	0.0365032	0.4224608	-3.751176
PROCR	-2.452007	21.163975	-3.714453	0.001011	0.1387497	-0.693463
ENST0000C	-2.453994	10.795517	-2.813732	0.0093333	0.2695793	-2.608294
lincRNA:ch	-2.461161	18.801417	-2.719687	0.0116345	0.2935077	-2.795515
lincRNA:ch	-2.464235	7.5651333	-3.616523	0.001297	0.1469235	-0.90951
KANK4	-2.47009	12.222817	-2.954948	0.0066702	0.2412766	-2.321589
lincRNA:ch	-2.470344	14.089133	-3.09099	0.004801	0.2225409	-2.039675
L1TD1	-2.47612	16.50215	-2.360913	0.0262222	0.3875594	-3.478023
ENST0000C	-2.47849	8.0756667	-3.286537	0.0029692	0.1914558	-1.62593
KBTBD3	-2.48918	15.315767	-2.998745	0.0060034	0.2352738	-2.23141
lincRNA:ch	-2.489905	10.799483	-2.702686	0.0121037	0.2962939	-2.829021
ENST0000C	-2.494056	8.9986167	-2.647965	0.0137373	0.30776	-2.936134
lincRNA:ch	-2.496308	8.0446333	-2.882454	0.0079314	0.2570639	-2.469572
C3orf36	-2.500752	7.7953167	-2.452056	0.0214283	0.360102	-3.309818
GZMB	-2.50591	39.762567	-3.569166	0.0014624	0.151208	-1.01349
lincRNA:ch	-2.506803	10.764367	-2.9763	0.0063367	0.2381317	-2.277695
A2LD1	-2.510817	25.080883	-2.284951	0.0309474	0.4033837	-3.615247
BBS12	-2.512755	18.421433	-3.163509	0.0040214	0.2107	-1.887334
SLC1A7	-2.515203	28.514617	-2.784477	0.0099986	0.276697	-2.666866
C11orf64	-2.517357	12.70465	-2.721994	0.0115722	0.2935077	-2.790961
GPR182	-2.52047	8.6604	-2.561687	0.0167372	0.3294068	-3.102654
LOC100129	-2.541597	22.052683	-2.543652	0.0174369	0.3346187	-3.137083
SIP1	-2.543898	13.105692	-3.084751	0.0048744	0.2225409	-2.052716
BX106347	-2.559885	8.80565	-2.374506	0.0254498	0.3836446	-3.453179
FLJ10088	-2.561138	12.450317	-2.2314	0.0347309	0.417973	-3.710294
ZNF572	-2.565936	20.5191	-3.013126	0.0057986	0.2321647	-2.201676
GTSF1	-2.57645	22.86795	-4.645555	9.07E-05	0.064665	1.3979683
LOC100132	-2.576959	14.927383	-2.555943	0.0169572	0.3316377	-3.113634
LOC253805	-2.576999	13.743217	-3.768212	0.0008812	0.1345828	-0.574333
A_24_P194	-2.577175	40.804867	-3.449924	0.0019751	0.1624754	-1.273695
LINGO2	-2.577253	19.088533	-2.463013	0.02091	0.3574747	-3.289347
UNQ5815	-2.57925	9.1604333	-2.444385	0.0217983	0.3617426	-3.32412
A_33_P342	-2.581424	8.1863833	-2.344071	0.0272089	0.3907322	-3.508684
LOC730139	-2.58301	9.3340333	-2.424306	0.0227948	0.3684802	-3.361429

LOC729047	-2.583049	7.2846667	-2.139344	0.0422226	0.442736	-3.870262
ENST00000	-2.585183	15.575583	-2.562529	0.0167052	0.3294068	-3.101044
lincRNA:ch	-2.588434	8.98695	-2.316707	0.0288849	0.3980667	-3.558217
XKR4	-2.588786	10.65785	-2.469106	0.0206267	0.3554645	-3.277939
OR6C76	-2.591547	8.4105	-2.494715	0.0194745	0.3470759	-3.229818
KIAA1586	-2.591566	8.1040167	-2.864882	0.0082696	0.2587405	-2.505189
LOC100130	-2.595052	7.7439833	-2.400795	0.0240148	0.3756903	-3.404886
ZFP30	-2.600515	21.394333	-3.703044	0.0010408	0.1394762	-0.7187
lincRNA:ch	-2.604059	18.26405	-3.665208	0.0011461	0.1429549	-0.802269
A_33_P331	-2.605234	9.20185	-3.441382	0.0020179	0.1628702	-1.29224
ENST00000	-2.607681	8.1258333	-2.413865	0.0233294	0.3720518	-3.38076
A_33_P328	-2.618255	9.2920333	-2.272202	0.0318126	0.4058114	-3.638006
LOC646976	-2.627105	11.842367	-3.340155	0.0025988	0.1784419	-1.510938
lincRNA:ch	-2.632607	9.73175	-2.558621	0.0168543	0.3303652	-3.108517
LOC732435	-2.650836	10.303767	-2.652709	0.0135879	0.3065743	-2.926893
ENST00000	-2.655418	7.4442667	-2.660894	0.0133337	0.3051802	-2.910929
lincRNA:ch	-2.656436	8.4583333	-2.83903	0.008792	0.2638971	-2.557409
lincRNA:ch	-2.658531	9.7238167	-4.110224	0.0003652	0.1057281	0.1902673
ENST00000	-2.67911	13.714633	-2.457492	0.0211697	0.359043	-3.299668
lincRNA:ch	-2.682185	9.17875	-2.165742	0.0399382	0.4352415	-3.824842
lincRNA:ch	-2.687001	17.96585	-2.932833	0.0070332	0.2472862	-2.366905
TIFAB	-2.688157	19.595333	-4.2612	0.0002468	0.0890075	0.5302733
A_33_P334	-2.695656	15.038217	-3.067941	0.0050777	0.2240985	-2.087805
ZNF547	-2.70295	12.839575	-3.710682	0.0010207	0.1387497	-0.701807
ENST00000	-2.703253	6.8038833	-2.352592	0.0267056	0.3884394	-3.493189
lincRNA:ch	-2.703684	9.02125	-3.301907	0.0028581	0.1888228	-1.59303
lincRNA:ch	-2.705055	7.8954167	-2.97154	0.0064097	0.2393269	-2.287493
C9orf73	-2.711027	8.876	-3.159984	0.0040563	0.2109581	-1.89477
NUDCD1	-2.713572	13.751967	-3.443271	0.0020083	0.1624754	-1.28814
KBTBD6	-2.73233	23.462133	-3.137701	0.0042838	0.215105	-1.941703
P2RY6	-2.735003	18.801358	-4.023765	0.0004568	0.1111414	-0.003905
ENST00000	-2.735737	9.1884333	-3.033466	0.0055204	0.229136	-2.159521
lincRNA:ch	-2.737656	19.590667	-2.139508	0.0422081	0.442736	-3.869982
NRXN2	-2.738439	13.527733	-3.946047	0.0005582	0.1164353	-0.178004
lincRNA:ch	-2.739066	20.677067	-2.214945	0.0359749	0.4210607	-3.739211
ANKRD58	-2.759801	32.391217	-3.556713	0.0015092	0.1515078	-1.040775
FLJ37543	-2.760604	8.715	-3.022729	0.0056656	0.2306829	-2.181788
lincRNA:ch	-2.769533	9.1182	-2.503485	0.0190938	0.3456829	-3.213272
ENST00000	-2.776856	11.457833	-2.843666	0.0086961	0.2636111	-2.548061
LOC441528	-2.780586	18.070558	-2.164331	0.0400575	0.4356864	-3.82728
lincRNA:ch	-2.790719	6.5564333	-2.448709	0.0215891	0.3614175	-3.316062
lincRNA:ch	-2.805894	8.20785	-2.269439	0.032003	0.4068648	-3.642927
INS-IGF2	-2.809242	6.3	-2.254705	0.0330357	0.4111299	-3.669107

ZMYND12	-2.810965	14.211867	-3.549214	0.001538	0.1515078	-1.057193
A_33_P327	-2.812649	7.7791	-2.739835	0.0111006	0.2886562	-2.755669
lincRNA:ch	-2.812708	10.28685	-3.990208	0.0004981	0.1160556	-0.079134
LOC100131	-2.81868	7.6274333	-2.613913	0.0148557	0.3167176	-3.002208
RIMBP3	-2.826277	26.1079	-4.0149	0.0004673	0.1111414	-0.023788
GSDMA	-2.840688	12.804167	-5.054028	3.13E-05	0.0638678	2.3166559
lincRNA:ch	-2.845622	15.521567	-3.680393	0.0011026	0.1401317	-0.768753
GSTA5	-2.845897	11.3512	-2.581637	0.0159937	0.3231011	-3.064415
lincRNA:ch	-2.846719	19.3389	-2.856346	0.0084387	0.2605348	-2.522457
GNG13	-2.852182	24.43245	-2.516795	0.0185293	0.3410209	-3.188102
lincRNA:ch	-2.853415	18.7516	-2.137638	0.0423743	0.4431046	-3.873186
ENST0000C	-2.855765	12.2052	-3.116639	0.00451	0.2201392	-1.98595
ARHGEF1(	-2.859838	21.279067	-2.629559	0.0143317	0.3122677	-2.971905
LOC100128	-2.866867	17.83075	-2.323805	0.0284413	0.3971217	-3.545403
lincRNA:ch	-2.870333	8.9208	-2.347522	0.027004	0.390281	-3.502413
lincRNA:ch	-2.873916	8.70275	-2.522654	0.0182857	0.3388496	-3.176998
ITLN1	-2.88725	20.1656	-2.888934	0.0078101	0.2563428	-2.456411
ACVRL1	-2.915133	17.528933	-2.374389	0.0254563	0.3836446	-3.453392
FPR3	-2.920664	23.977392	-3.186357	0.0038021	0.2066715	-1.839059
AQP10	-2.921281	25.856367	-2.333399	0.0278517	0.3931293	-3.528044
OR7E85P	-2.935144	9.9621667	-2.362589	0.0261258	0.3873866	-3.474964
OR4N3P	-2.939961	7.0592667	-2.874972	0.0080738	0.2574548	-2.48475
OR4E2	-2.954098	6.4547	-3.105615	0.0046329	0.2220927	-2.009062
lincRNA:ch	-2.95821	20.454	-2.076041	0.0481845	0.4599078	-3.977655
lincRNA:ch	-3.006808	21.701167	-2.457734	0.0211582	0.359043	-3.299216
ZSWIM3	-3.032126	21.212217	-3.415515	0.002153	0.1687148	-1.348314
lincRNA:ch	-3.037452	10.65715	-2.385041	0.0248655	0.3810375	-3.433864
RPS12	-3.038881	42.122267	-3.636266	0.0012336	0.1452138	-0.866062
DIRC1	-3.065843	28.438317	-3.036243	0.0054834	0.228143	-2.153757
BCL8	-3.073166	10.74395	-2.41229	0.023411	0.3722428	-3.38367
ENST0000C	-3.077709	9.4410167	-3.645909	0.0012037	0.14461	-0.844821
lincRNA:ch	-3.077964	21.786333	-2.392049	0.0244837	0.3787871	-3.420987
lincRNA:ch	-3.080881	13.528317	-3.97241	0.0005215	0.1164353	-0.118999
lincRNA:ch	-3.081762	11.530167	-2.206882	0.036599	0.4224608	-3.753328
lincRNA:ch	-3.085913	15.5561	-2.635792	0.0141278	0.3108157	-2.959806
PRB3	-3.089927	22.862817	-3.876702	0.0006672	0.1233446	-0.332921
ENST0000C	-3.09635	6.9452833	-3.813318	0.0007851	0.1300441	-0.474116
lincRNA:ch	-3.100716	11.2119	-3.437025	0.0020401	0.1635269	-1.301693
LOC100128	-3.103869	9.5044833	-3.330365	0.0026629	0.1817721	-1.53198
ENST0000C	-3.132241	13.967333	-3.389584	0.0022974	0.1688885	-1.4044
lincRNA:ch	-3.135138	10.2606	-3.424173	0.0021069	0.1665891	-1.329559
FAM43A	-3.161494	26.755633	-3.250329	0.0032475	0.1994989	-1.703229
LOC253044	-3.165606	8.0733333	-3.068935	0.0050655	0.2240985	-2.085733

ZNF540	-3.169913	14.595	-3.386309	0.0023162	0.1697064	-1.411472
FASLG	-3.173849	26.38041	-3.221629	0.0034859	0.2049084	-1.764286
MUC5AC	-3.178548	21.08505	-2.40472	0.023807	0.3747166	-3.397648
HSPB3	-3.181387	13.190567	-2.769141	0.010365	0.2806665	-2.697452
DDIT4L	-3.1906	14.296158	-3.041518	0.0054138	0.2266983	-2.142801
RTP4	-3.192705	32.0488	-3.194477	0.0037269	0.2061786	-1.821873
ANXA13	-3.196053	8.60335	-4.5409	0.0001191	0.0677403	1.1617044
RFPL4A	-3.23025	16.535808	-2.232542	0.034646	0.4176118	-3.708282
LOC728073	-3.230789	9.1099167	-2.999526	0.0059921	0.2352738	-2.229797
LOC286087	-3.235253	20.860117	-3.376394	0.0023743	0.1706151	-1.432876
lincRNA:ch	-3.242087	13.135033	-2.21341	0.036093	0.4212464	-3.741901
SPANXN4	-3.274629	10.943333	-2.865727	0.008253	0.2587405	-2.503479
hCG_17760	-3.291469	8.3640667	-2.519602	0.0184122	0.3398028	-3.182784
RAX2	-3.311303	11.36205	-3.644579	0.0012078	0.14461	-0.847751
IFNG	-3.327551	19.86586	-3.163774	0.0040188	0.2107	-1.886773
POM121L8	-3.336278	11.172758	-3.08446	0.0048779	0.2225409	-2.053325
lincRNA:ch	-3.34042	8.7117333	-4.986936	3.72E-05	0.064665	2.1663302
CR745709	-3.350386	8.9125167	-3.162214	0.0040342	0.2107	-1.890066
A_33_P321	-3.384554	14.2583	-3.078663	0.0049471	0.2225409	-2.065434
DKFZp434	-3.400708	12.89435	-2.746426	0.010931	0.2862016	-2.742603
LOC645249	-3.414571	8.18895	-3.10554	0.0046338	0.2220927	-2.009219
lincRNA:ch	-3.44261	35.504817	-3.218553	0.0035125	0.2056835	-1.770819
SNX31	-3.446722	8.5793167	-3.115829	0.0045189	0.2202668	-1.987648
LOC440300	-3.469924	13.498567	-5.686892	6.08E-06	0.0416449	3.7164072
HOXA2	-3.489368	10.636617	-3.51741	0.0016666	0.1530728	-1.126727
lincRNA:ch	-3.497337	14.7567	-3.787788	0.0008382	0.1319538	-0.530868
ENST00000	-3.501938	7.3851167	-2.960206	0.0065866	0.2412766	-2.310793
lincRNA:ch	-3.510476	8.1555833	-4.335802	0.0002033	0.0875525	0.6985805
LOC283761	-3.511533	14.302283	-5.206988	2.10E-05	0.0564492	2.6582231
CTTNBP2	-3.512453	9.4875667	-2.346537	0.0270624	0.390281	-3.504203
ENST00000	-3.548618	9.93685	-2.611026	0.0149543	0.3172273	-3.007789
lincRNA:ch	-3.561228	14.839183	-5.487386	1.02E-05	0.0416449	3.2792943
lincRNA:ch	-3.593653	14.657183	-2.12609	0.0434133	0.4460421	-3.892928
ENST00000	-3.610688	12.233083	-2.701747	0.0121301	0.2962939	-2.830868
A_33_P340	-3.653687	9.1236833	-2.843815	0.008693	0.2636111	-2.54776
A_33_P330	-3.692103	18.581383	-2.200139	0.0371285	0.4242006	-3.765111
ADARB2	-3.706358	16.822517	-3.241899	0.0033159	0.200491	-1.721182
LOC100133	-3.751041	12.87055	-3.041046	0.00542	0.2266983	-2.143782
lincRNA:ch	-3.753997	21.001167	-2.221101	0.0355048	0.4209153	-3.728408
A_33_P321	-3.818201	19.251517	-2.915656	0.007328	0.2505573	-2.401999
RNU11	-3.874201	8.7659833	-2.854056	0.0084847	0.2606871	-2.527083
TRGV7	-3.891099	24.937267	-3.513462	0.0016833	0.1536017	-1.135344
A_33_P332	-3.973317	10.45135	-3.7653	0.0008878	0.1345828	-0.580796

TMEM213	-3.981757	8.5402333	-2.915712	0.007327	0.2505573	-2.401885
VMO1	-4.029317	22.321017	-3.094547	0.0047596	0.2225409	-2.032234
HIST1H2AI	-4.178657	29.475833	-3.463222	0.0019102	0.1617143	-1.244798
lincRNA:ch	-5.285695	11.445933	-2.751159	0.0108107	0.2849344	-2.73321
MYOM2	-6.596957	30.176067	-2.860312	0.0083597	0.260164	-2.514436
