# Supplementary material for: Comprehensive Analysis Based on Genes Associated With Cuproptosis, Ferroptosis, and Pyroptosis for the Prediction of Diagnosis and Therapies in Coronary Artery Disease
Source: Cardiovasc Ther. 2025 Mar 15;2025:9106621. doi: 10.1155/cdr/9106621 (PMC11929595; doi:10.1155/cdr/9106621)
Supplement: Supporting Information 5 — Table S4: The small molecules drugs targeting in genes from Connective Map (CMap) and DGIdb database. [file 9106621.f5.pdf]

Supplementary Table 4. The small molecules drugs targeting in genes from Connective map (CMap) and DGIdb database

Symbol	Drugs	Scores
STK17B	quercetin	81
JUNB	dexamethasone	76
NLRP1	perhexiline maleate	3.98
MARCKS	BIO-11006	63.65
